# eQTL analysis of laying hens divergently selected for feather pecking identifies KLF14 as a potential key regulator for this behavioral disorder

**DOI:** 10.3389/fgene.2022.969752

**Published:** 2022-08-17

**Authors:** Alexander Charles Mott, Andrea Mott, Siegfried Preuß, Jörn Bennewitz, Jens Tetens, Clemens Falker-Gieske

**Affiliations:** ^1^ Department of Animal Sciences, Georg-August-University, Göttingen, Germany; ^2^ Institute of Animal Science, University of Hohenheim, Stuttgart, Germany; ^3^ Center for Integrated Breeding Research, Georg-August-University, Göttingen, Germany

**Keywords:** feather pecking, eQTL, AWM, genomics, transcriptomics, gene regulation, behavioral disorder

## Abstract

Feather pecking in chickens is a damaging behavior, seriously impacting animal welfare and leading to economic losses. Feather pecking is a complex trait, which is partly under genetic control. Different hypotheses have been proposed to explain the etiology of feather pecking and notably, several studies have identified similarities between feather pecking and human mental disorders such as obsessive-compulsive disorder and schizophrenia. This study uses transcriptomic and phenotypic data from 167 chickens to map expression quantitative trait loci and to identify regulatory genes with a significant effect on this behavioral disorder using an association weight matrix approach. From 70 of the analyzed differentially expressed genes, 11,790 genome wide significantly associated variants were detected, of which 23 showed multiple associations (≥15). These were located in proximity to a number of genes, which are transcription regulators involved in chromatin binding, nucleic acid metabolism, protein translation and putative regulatory RNAs. The association weight matrix identified 36 genes and the two transcription factors: *SP6* (synonym: *KLF14*) and *ENSGALG00000042129* (synonym: *CHTOP*) as the most significant, with an enrichment of KLF14 binding sites being detectable in 40 differentially expressed genes. This indicates that differential expression between animals showing high and low levels of feather pecking was significantly associated with a genetic variant in proximity to *KLF14*. This multiallelic variant was located 652 bp downstream of *KLF14* and is a deletion of 1-3 bp. We propose that a deletion downstream of the transcription factor *KLF14* has a negative impact on the level of T cells in the developing brain of high feather pecking chickens, which leads to developmental and behavioral abnormalities. The lack of CD4 T cells and gamma-Aminobutyric acid (GABA) receptors are important factors for the increased propensity of laying hens to perform feather pecking. As such, *KLF14* is a clear candidate regulator for the expression of genes involved in the pathogenic development. By further elucidating the regulatory pathways involved in feather pecking we hope to take significant steps forward in explaining and understanding other mental disorders, not just in chickens.

## Introduction

Feather pecking (FP) behavior in chickens is a serious issue, which has an impact on animal welfare, where damage to the feathers, bodily injuries and even death can occur (Hughes and Duncan, 1972; Allen and Perry, 1975). As well as this negative impact on animal welfare, there is also a financial impact for the chicken producer, through the increased animal care costs, and ultimately with the loss of injured animals (Huber-eicher and Wechsler, 1997). FP is a trait observed in chickens that are kept in all types of housing (Appleby and Hughes, 1991). Previous attempts to manage this disorder have seen producers utilizing beak trimming, a process that is now being banned in more and more countries due to its impact on the welfare of individual animals (Duncan et al., 1989; Gentle et al., 1990).

Some studies have hypothesized that pecking at the plumage of other birds is related to foraging behavior and occurs due to the low incentive value of floors without litter, (Hoffmeyer, 1969; Blokhuis, 1986; Huber-eicher and Wechsler, 1997), while others have indicated links with behavioral traits such as general locomotor activity (GLA), and fearfulness (Vestergaard et al., 1993; Kjaer, 2017). This suggests that the reasons underpinning FP are associated with both environmental and genetic factors, with a number of studies indicating the possibility of reducing FP by means of breeding, to select for low FP (Kjaer and Sørensen, 1997; Rodenburg et al., 2003; Bennewitz et al., 2014). Quantitative trait loci (QTL) mapping studies and genome wide association studies (GWAS) confirmed the assumption that this trait is polygenically determined with some trait-associated chromosomal regions (Buitenhuis et al., 2003; Lutz et al., 2017; Falker-Gieske et al., 2020a).

There have been a number of studies undertaken to attempt to describe the underlying processes of FP, using gene expression analysis and mapping studies to link FP to immune response and multiple signaling pathways (Wysocki et al., 2010; Haas and van der Eijk, 2018), with some studies linking FP to schizophrenia and obsessive-compulsive disorders, indicating its use as a potential model for these diseases (Falker-Gieske et al., 2021). However, these studies have so far been unable to elucidate the exact mechanism for this feather pecking.

Here the transcriptomic and phenotypic data from 167 chickens was analyzed through a comprehensive strategy combining expression quantitative trait loci (eQTL) mapping and the network analysis of gene-gene interactions to predict SNPs and regulatory pathways that had significant impacts on these abnormal behavior patterns. It is also hoped that the data presented here can be used as a possible model system for both obsessive-compulsive disorder and schizophrenia, due to the similarities of the disease (Falker-Gieske et al., 2021). As such, further elucidating the pathways involved in FP could prove significant in explaining and understanding other mental disorders, not just in chickens.

## Methods

### Sample collection

The samples used in the following study were collected as previously described in Falker-Gieske et al*.* (2020a); Falker-Gieske et al., 2020b). Briefly, White Leghorn strains were selected for more than ten generations based on estimated breeding values for feather pecking (Grams et al., 2015). These lines were created and are maintained at the Hohenheim University and neither commercially obtained nor from a private source, with rearing and husbandry conditions being previously described (Bennewitz et al., 2014; Falker-Gieske et al., 2020b). A total of 492 birds from two experimental runs were separated into 7 groups of 39–42 birds (ratio of 1:1 LFP-HFP), and 6 groups of 39–42 birds (ratio of 1:2 LFP-HFP) (Iffland et al., 2020b). At 31–33 weeks, the hens, were phenotyped according to established ethograms. In short, feather pecking [feather pecks delivered (FPD), and feather pecks received (FPR)], and aggressive behavior [aggressive pecks delivered (APD) and aggressive pecks received (APR)], were recorded and hens were marked with numbered plastic batches on their backs to aid in identification (Lutz et al., 2017; Falker-Gieske et al., 2020a; Iffland et al., 2020a). Observation of FP behavior was done in 20-min sessions on four consecutive days by a minimum of six different trained observers. To prevent FP, birds were kept under low light conditions. One bird from each full-sib pair kept under dark conditions was sacrificed, and due to time constraints, the whole brains were immediately collected for RNA isolation in order to preserve expression levels. Chickens were CO_2_-stunned and sacrificed by ventral neck cutting. For light stimulation, the remaining birds were kept under increased light intensity (≥100 lux) for several hours. Upon initiation of FP behavior these birds were then sacrificed and brains were collected for RNA isolation. 48 birds were utilized for the transcriptomic analysis described previously (Falker-Gieske et al., 2020b), with 167 animals in total (the previously mentioned 48 animals plus a further 119 animals) being collected and utilized in this study.

### Generation of cDNA

cDNA needed for the HT-qPCR analysis was generated from selected high feather pecking (HFP) and low feather pecking (LFP) animals. RNA was extracted using an RNeasy kit (Qiagen, Hilden, Germany) as per the provided protocol. cDNA preparation was then performed using the Fluidigm Reverse Transcription assay kit (Fluidigm Corporation, San Francisco, CA, United States).

### Quantitative gene expression analysis

In order to quantitatively analyze the gene expression in feather pecking, HT-qPCR analysis was performed for 86 differentially expressed genes (DEGs) highlighted by our previous transcriptomic study (Falker-Gieske et al., 2020b). A Fluidigm BioMark system, using a 96.96 Dynamic Array integrated fluidic circuit for gene expression (IFC; Fluidigm Corporation, San Francisco, CA, United States), and Delta Gene Assays (Fluidigm Corporation, San Francisco, CA, United States) were used, and delta Ct values were obtained by normalization against *GAPDH*. The primers utilised in this study can be found in the Supplementary Material S1

### Detection of expression quantitative trait loci

Whole genome sequencing genotypes mapped to chicken reference assembly GRCg6a (GCF_000002315.5 RefSeq assembly) from our previous study (Falker-Gieske et al., 2020a) were used for expression genome wide associations studies (eGWAS). Imputation from 60k chip genotypes to whole genome sequence level was performed with Beagle 5.2 (Browning et al., 2018) to make use of the latest imputation algorithm and with the setting ne = 1000 to factor in the small effective population size. Since the default setting is ne = 1000000 we adjusted the setting according to conclusions of Pook *et al.* who showed that this improves imputation accuracy in small populations (Pook et al., 2020). eGWAS were performed with gcta v. 1.92.3 beta3 (Yang et al., 2011) with the setting maf 0.01 and hatch as covariate. Population structure was accounted for in the linear mixed model based on the GRM, which is included in the analysis.

### Association weight matrix construction

An association weight matrix (AWM) utilizes the results of multiple GWAS as the basis for calculation of gene-gene or gene-variant associations. Followed by the application of network inference algorithms it generates gene networks with regulatory and functional significance. Input variants for the AWM were selected to contain only highly associated signals, either with the main phenotype (feather pecks delivered box-cox-transformed, FPD_BC) or multiple of the gene expression phenotypes. Since the AWM analysis was designed for SNP chip data, stringent pre-filtering of whole genome sequencing data is necessary to produce a compatible input dataset. Therefore, variants with a *p*-value < 1 × 10^–5^ for the main phenotype or variants that were associated (*p* < 1 × 10^–5^) with at least ten of the gene expression phenotypes were retained. This resulted in the selection of 2,753 variants, which represent 0.042% of all genome wide variants that were studied. Distances up to 100 kb to the closest genes for each variant were predicted with the Ensembl Variant Effect Predictor (Assembly: GRCg6a, accessed Jan. 13th 2022). The AWM was constructed with an established method by Reverter and Fortes (Reverter and Fortes, 2013). The analysis was performed with the following settings: the *p*-value thresholds for primary SNP selection and selection of non-key phenotype SNPs were set to 5 × 10^–6^, the *p*-value threshold for secondary SNP selection was set to 1 × 10^–9^. SNP based variants with a distance ≤ 2,500 bp to the closest gene were considered close and variants with a distance ≥ 20,000 bp were considered far. Associations between SNP based variants and phenotypes, the gene expression values for DEGs between high feather peckers (HFP) and low feather peckers (LFP), were analyzed with the partial correlation and information (PCIT) algorithm (Reverter and Chan, 2008) to retrieve significant associations between the closest genes to SNP based variants and DEGs. The analysis with the PCIT algorithm results in pairs of genes, which are predicted to interact. The gene-gene interaction network was visualized with Cytoscape (Shannon et al., 2003) and gene ontology classifications were assigned with PANTHER v. 16.0 (Mi et al., 2017). Genes, which were not annotated by PANTHER were manually annotated using information provided on the UniProt website (accessed March 2022) (UniProt: the universal protein knowledgebase in 2021., 2021).

### Transcription factor enrichment

Transcription factor enrichment analysis was performed with CiiiDER (build 15 May 2020) (Gearing et al., 2019). Genes that reached genome wide significance (*p*-value < 7.6 × 10^–9^) for at least one variant associated with the transcription factor were used as input. Background genes with an absolute *log*_*2*_ fold-change (abs. LFC) < 0.5 were selected from the differential expression analysis results from our previous study (Falker-Gieske et al., 2020b), which resulted in a set of 19,088 genes. The *p*-value threshold for gene coverage enrichment was set to 0.05, base position upstream scan limit to 1,500 bp, and base position downstream scan limit to 500 bp. To predict transcription factor binding to a target DNA sequence, a position frequency matrix (PFM) is required, which assigns nucleotide frequencies to each position in the binding motif. For the analysis of SP6 (synonym: KLF14) the PFM MA0740.1 (https://testjaspar.uio.no/matrix/MA0740.1/) was used. Additionally the PFMs of all KLF transcriptions factors available on the Jaspar homepage have been included in the analysis, namely: KLF1 (MA0493.2), KLF10 (MA1511.2), KLF11 (MA1512.1), KLF12 (MA0742.2), KLF13 (MA0657.1), KLF15 (MA1513.1), Klf15 (MA 1963.1), KLF16 (MA0741.1), KLF2 (MA1515.1), KLF3 (MA1516.1), Klf3/8/12 (MA 1964.1), KLF4 (MA0039.4), KLF5 (MA0599.1), Klf5-like (MA 1965.1), KLF6 (MA1517.1), Klf6-7-like (MA 1966.1), KLF7 (MA 1870.1), and KLF9 (MA1107.2). Binding sites of *ENSGALG00000042129* (synonym: *CHTOP*) were screened with PFM MA1153.1 (https://jaspar.genereg.net/matrix/MA1153.1/). All PFMs were acquired in JASPAR format (Castro-Mondragon et al., 2022). The transcription factor enrichment plot was created with CiiiDER.

## Results

**FIGURE 1:**
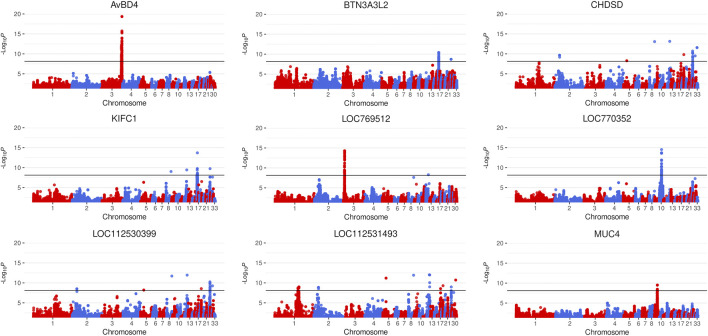
Manhattan plots of expression genome wide association studies (eGWAS) that yielded highly significant QTL, conducted on genes differentially expressed between high and low feather pecking hens (Bonferroni threshold $\left(\frac{number\;of\;variants}{0.05}\right)$ : *p*-value = 7.6 × 10^–9^).

Conducting an eGWAS on 86 genes that were differentially expressed between 167 chickens (84 HFP and 83 LFP, normalized gene expression results in Supplementary Material S2) yielded Manhattan plots with prominent peaks for the genes *AvBD4*, *BTN3A3L2*, *CHDSD*, *KIFC1*, *LOC769512*, *LOC770352*, *LOC112530399*, *LOC112531493*, and *MUC4* (Figure 1, Manhattan plots for all differentially expressed genes (DEGs) in Supplementary Material S3). QTL for these eGWAS are shown in Supplementary Material S4. We discovered 41 genome wide significant variants in proximity to (<1 kb distance to gene start or gene end) the DEGs *AvBD4*, *LOC769512*, and *MUC4* (Supplementary Material S5), which are candidates for being cis-regulatory elements. Although QTL regions were detected through the eGWAS, the use of an associated weight matrix allowed for the further recovery of associated variants from this data set. In total 11,790 genome wide significant associations (Bonferroni threshold *p*-value = 7.6 × 10^–9^) for 70 of the analyzed DEGs were detected, of which 9,677 were unique. Stringent filtering (*p*-value < 1 × 10^–8^ in more than 15 eGWAS) was applied, which yielded 23 highly associated variants (Table 1). Due to the nature of an eQTL study one would expect to also discover trans-regulatory elements and regulators of transcription to be associated with DEGs (Gilad et al., 2008). Unsurprisingly, the PANTHER Classification System revealed *KLF14* and *CHTOP* belong to the protein class “gene-specific transcriptional regulator (PC00264)”, *ASH1L* to the class “chromatin/chromatin-binding, or -regulatory protein (PC00077)”, *ENSGALG00000042129* to the class “nucleic acid metabolism protein (PC00171)”, and *DAP3* to the class “translational protein (PC00263)”. Furthermore, as in previous studies we found putative regulatory RNAs, namely long non-coding RNAs (lncRNAs) among the top associated genes: *ENSGALG00000050482*, *ENSGALG00000048366*, *ENSGALG00000046959*, and *ENSGALG00000054815*.

**TABLE 1:** Genomic variants with a *p*-value < 1 × 10^–8^ in more than 15 expression genome wide association studies (eGWAS). Only one variant per gene is shown since they represent the respective associated haplotype block. Closest genes up to a distance of 100 kb were considered.

Position	Variant ID	Consequence	Closest gene	Distance	Gene product	No. of associations
12:15059746	rs318185887	downstream_gene_variant	ENSGALG00000050482	1179	lncRNA	56
8:28987015	8_28987015	upstream_gene_variant	SLC35D1	11161	UDP-glucuronic acid/UDP-N-acetylgalactosamine transporter	54
19:842041	19_842041	intron_variant	MTMR4	0	Myotubularin-related protein 4	42
25:3562220	rs1060122522	downstream_gene_variant	ASH1L	2176	Histone-lysine N-methyltransferase ASH1L	40
27:6521246	27_6521246	downstream_gene_variant	SP6 /KLF14	652	Transcription factor Sp6 /Krueppel-like factor 14	40
17:6424769	17_6424769	intron_variant	NUP214	0	Nuclear pore complex protein Nup214	33
25:3026965	rs737629884	missense_variant	ENSGALG00000042129 /CHTOP	0	Chromatin target of PRMT1	31
5:16304718	rs314040024	intron_variant	ENSGALG00000039221	0	USP6 N-terminal like	25
2:23739061	2_23739061	intron_variant	PPP1R9A	0	Neurabin-1	25
25:3312173	25_3312173	downstream_gene_variant	ENSGALG00000037599	177	S100 calcium binding protein A4	24
2:23413545	rs734790703	—	—	—		22
2:23413552	rs15074227	—	—	—		22
2:23414512	rs14152082	upstream_gene_variant	ENSGALG00000048366	10205	lncRNA	22
25:3040753	25_3040753	downstream_gene_variant	ENSGALG00000046959	276	lncRNA	19
25:3026965	25_3026965	upstream_gene_variant	ENSGALG00000053285	60	Mothers against decapentaplegic homolog 6-like	17
25:3033290	25_3033290	downstream_gene_variant	ENSGALG00000054815	2351	lncRNA	17
25:3602675	rs1058400187	intron_variant	DAP3	0	28S ribosomal protein S29, mitochondrial	16

**FIGURE 2:**
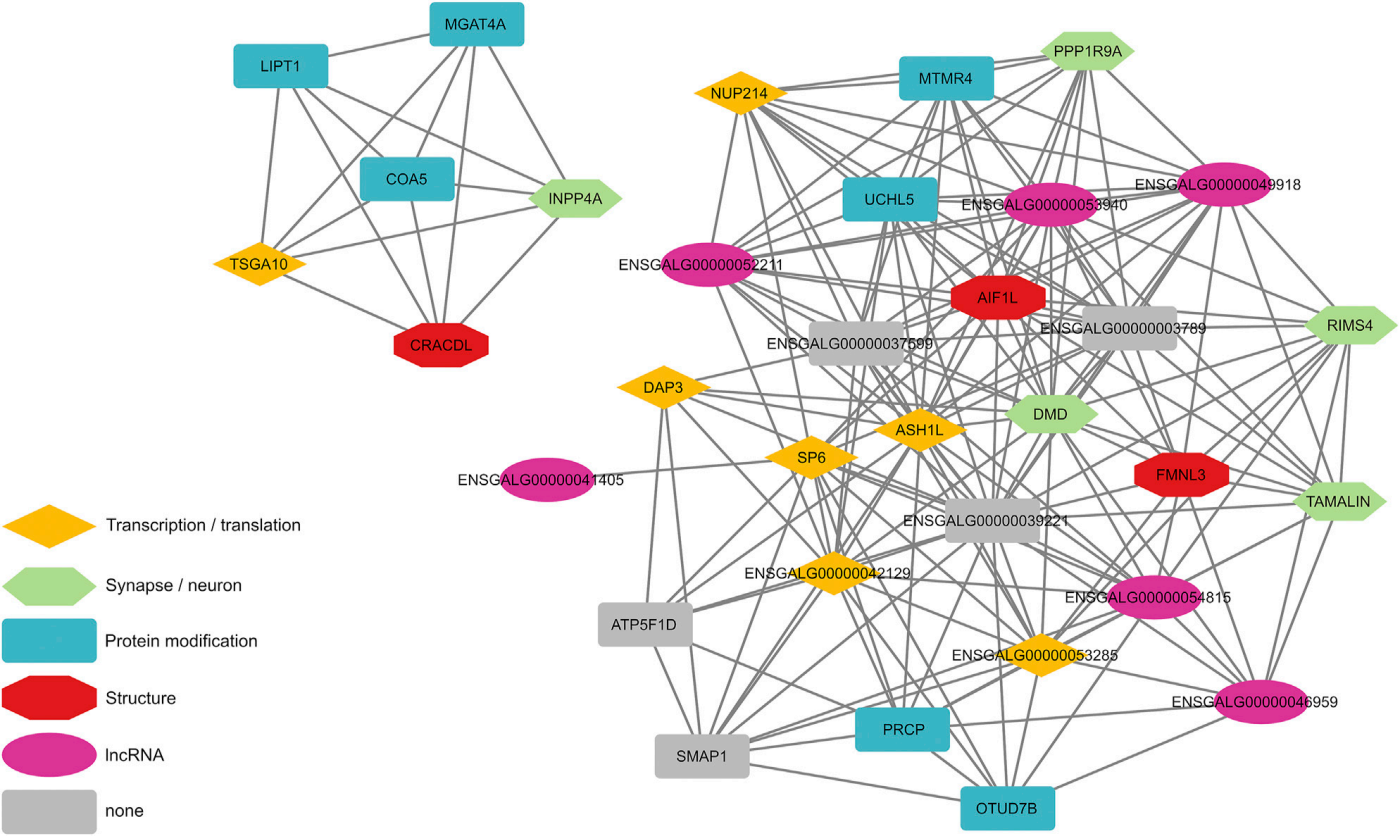
Significant gene-gene interactions, which were discovered with an association weight matrix followed by analysis with the PCIT algorithm. Input genes were derived from expression genome wide association studies with differentially expressed genes between high and low feather pecking chickens as phenotypes.

To further analyze the dataset at hand we created an AWM using highly associated eGWAS signals (*p*-value < 1 × 10^–5^ for the main phenotype (FPD_BC) or *p*-value < 1 × 10^–5^ in at least 10 eGWAS) from the 86 DEGs that were analyzed in this study. Significant associations between genes that are close to variants that indicated significant association to DEGs between HFP and LFP were identified with the PCIT algorithm (Reverter and Chan, 2008), which detects meaningful gene-gene associations in co-expression networks. This led to the discovery of 34 genes based on 2,753 input variants, which we visualized in a gene association interaction map, where nodes located at the center of the network indicates their centrality in the interaction cluster (Figure 2). Among these were four genes, which were associated with at least 30 DEGs (Table 1): *ASH1L*, *KLF14*, *NUP214*, and *CHTOP*, all of which are involved in gene transcription or translation.

**FIGURE 3:**
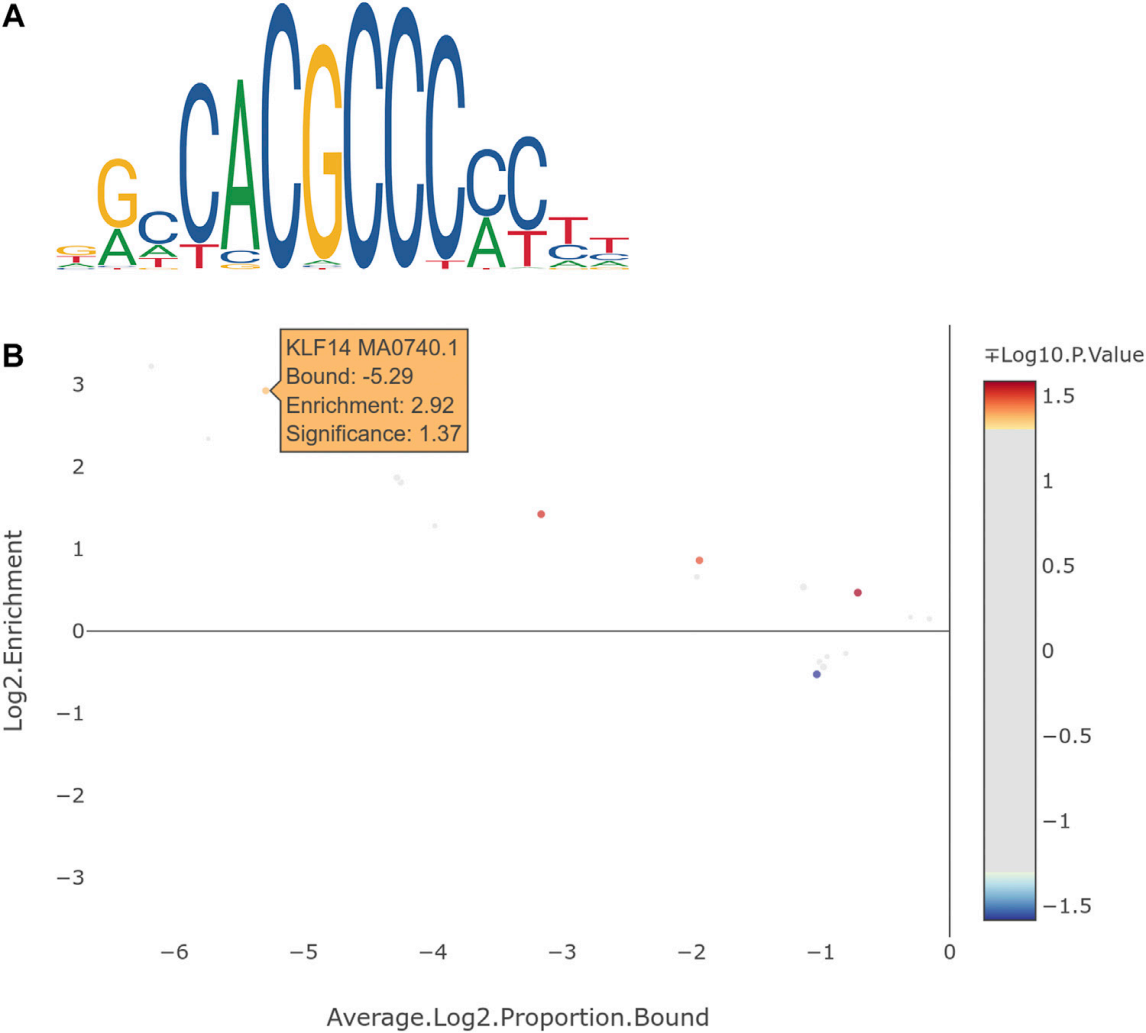
**(A)** Graphical representation of the position frequency matrix of the *KLF14* transcription factor. **(B)** Result of transcription factor binding site enrichment analysis for *KLF14* with genes from expression genome wide associations studies with genome wide significant association. All available KLF transcription factors were included as controls for the specificity of *KLF14* binding site enrichment.

Since *KLF14* and *CHTOP* are transcription factors, we decided to conduct transcription factor binding site enrichment analysis to clarify if DEGs, which were significantly associated with *KLF14* or *CHTOP*, are potential targets of these transcription factors. NCBI Conserved Domain Search (Marchler-Bauer et al., 2015) revealed that *CHTOP* contains an N-terminal Mad Homology 1 (MH1) domain as in the transcription factor *SMAD4*, which recognizes the palindromic DNA sequence GTCTAGAC. Transcription factor binding site enrichment analysis of putative target genes that were associated with genome wide significance (*p*-value < 7.6 × 10^–9^) yielded no significant result for *CHTOP* with the *SMAD4* recognition site. eGWAS revealed that 40 of the DEGs (Supplementary Material S6) between HFP and LFP were significantly associated with a multiallelic variant located 652 bp downstream of *KLF14* (Chromosome: 27 Position: 6521246 bp) and which is a deletion of 1–3 bp. An enrichment of *KLF14* binding sites (Figure 3A) was detectable in those 40 genes (Figure 3B). To ensure, that this enrichment is significant in relation to other transcription factor binding sites, all available PFMs for KLF transcription factors were included in the analyses with the complete results summarized in Supplementary Material S7. Although significant enrichment of binding sites for other KLFs were detected, *KLF14* resulted in the highest *log*_*2*_ enrichment value, while being statistically significant.

Out of those 40 putative *KLF14* regulated target genes 10 are involved in the immune system with the majority being leukocyte-immunoglobulin receptors (Table 2).

**TABLE 2:** Differentially expressed genes between high and low feather peckers significantly associated with *KLF14*, which are involved in the immune system. Obsolete gene Symbols, due to the recent release of new genome assemblies, are shown in parentheses.

Symbol	Description	Functions or other designations
BLEC2	C-type lectin-like receptor 2	lectin-like natural killer cell surface protein
CHIR-IG1-5	immunoglobulin-like receptor CHIR-AB1-like	immunoglobulin-like receptor CHIR-Ig1-5
CSF2RB	colony stimulating factor 2 receptor beta common subunit	cytokine receptor common subunit beta
LOC100857964	platelet glycoprotein VI-like	leukocyte immunoglobulin-like receptor subfamily B member 5
LOC107049819	leukocyte immunoglobulin-like receptor subfamily B member 3	leukocyte immunoglsobulin-like receptor subfamily A member 2
LOC107050473	platelet glycoprotein VI-like	leukocyte immunoglobulin-like receptor subfamily A member 2
LOC112531100	leukocyte immunoglobulin-like receptor subfamily A member 2	leukocyte immunoglobulin-like receptor subfamily A member 2
CLEC2D2L	C-type lectin domain family 2 member D2-like	natural killer cell lectin-like receptor binding
MHCY2B3P (LOC112533562)	major histocompatibility complex Y, class II beta 3 pseudogene	leukocyte receptor cluster member 9-like
MHCY35 (HLA.F10AL3)	major histocompatibility complex Y, class I heavy chain 35	major histocompatibility complex Y, class I heavy chain 35

## Discussion

To untangle the regulatory network behind feather pecking behavior we conducted an eQTL study on 86 genes that were differentially expressed between divergently selected HFP and LFP (Falker-Gieske et al., 2020b) in 167 chickens. The main result of this study is a gene-gene interaction map based on an AWM, which revealed two clusters of interacting genes (Figure 2). A number of those genes have previously been described to be involved with brain-related functions and have generally not been detected by simple filtering based on eGWAS *p*-values (Table 1). The *DMD* gene encodes the protein dystrophin, which colocalizes with GABA_A_ receptors in postsynaptic densities of neurons in the cerebral cortex of mice. Dystrophin deficient mice show an altered clustering of GABA_A_ receptors (Knuesel et al., 1999). We previously reported that mutations within or close to GABA receptors as well as differential expression of *GABRA2*, *GABRB2*, *GABRE*, and *GABRG3* are associated with feather pecking (Falker-Gieske et al., 2021). The central position of *DMD* in the gene-gene interaction network provides further evidence for our theory that feather pecking is a GABAergic dysfunction disorder [already discussed here (Falker-Gieske et al., 2021)]. A lack of GABA receptor expression has been linked to schizophrenia in humans [reviewed here (Chiapponi et al., 2016)]. In previous studies we found a considerable amount of genes involved in schizophrenia to be associated with feather pecking (Falker-Gieske et al., 2020a; Falker-Gieske et al., 2020b). The results of the AWM extend this list with *PPP1R9A,* which encodes the protein Neurabin-1 and has been linked to dendritic spine loss in schizophrenia (Konopaske et al., 2015). *TAMALIN*, a trafficking molecule of Metabotropic glutamate receptor 5 (mGluR5), showed increased expression in the hippocampal region of individuals with schizophrenia (Matosin et al., 2015). Furthermore, we discovered *INPP4A*, a gene linked to schizophrenia (Föcking et al., 2015), epilepsy (Wang et al., 2012), and intellectual disability (Banihashemi et al., 2020) as well as *RIMS4*, which is involved in synaptic plasticity and the development of autism (Leblond et al., 2019). Genes that have been classified as protein modifiers in the gene-gene interaction map have also been implicated in neurological disorders. A *LIPT1* deficiency has been reported in a case of early infantile epileptic encephalopathy (Stowe et al., 2018). Low expression of *MGAT4A* in the dorsolateral prefrontal cortex was reported in cases of schizophrenia (Kippe et al., 2015). We previously proposed that chickens selected for FP behavior could serve as a model for human psychiatric disorders (Falker-Gieske et al., 2021). In this respect Johnsson *et al.* conducted a study on an intercross between domestic chickens and Red Junglefowl focusing on the suitability of chickens as a model for anxiety behavior. Here, two genes also discovered in our study *LOC770352* and *GABRB2,* were identified as top candidate genes affecting stress and anxiety behavior (Johnsson et al., 2016). As this study did not use selection lines, their findings indicate that results derived from selection lines are clearly relevant in the general production populations. Furthermore, these common findings of homologous genes such as SP6/KLF14, *GABRB2* and *LOC770352* underlines the suitability of chickens as model organisms for behavioral disorders of the brain.

With *AvBD4*, *LOC769512*, and *MUC4* we discovered three potential cis-regulated DEGs in eGWAS. However, from a regulatory point of view those genes identified by the PCIT algorithm that are involved in transcription and translation are the most relevant. Variants in the *NUP214* gene cause acute febrile encephalopathy (Fichtman et al., 2019) and rare mutations in *ASH1L* are connected to numerous neurodevelopmental disorders [reviewed in (Zhang et al., 2021)]. Furthermore, among those genes were two transcription factors - *KLF14* and *CHTOP.* Whilst we found the presence of a missense mutation in *CHTOP*, leading to an alanine being replaced with a valine, no significance in the enrichment analysis was observed. Dysregulation at the cellular level of *CHTOP* has been shown however, to play an important role in the tumorigenicity of glioblastoma cells (van Dijk et al., 2010b), as well as being a critical regulator of γ-globin gene expression (van Dijk et al., 2010a). Although very little is still known about the molecular mechanism of transcriptional control that it mediates, it has also been associated with the methylosome complex containing PRMT1, PRMT5, MEP50, and ERH, which are critical for mammalian development through transcription regulation, DNA repair, RNA splicing, and signal transduction (Fanis et al., 2012; Liang et al., 2021) (Fanis et al., 2012)*.* In fact, Hannon *et al.* identified robust psychosis associated differences derived from DNA methylation, with increased proportions of monocytes and granulocytes and decreased proportions of natural killer cells, CD4^+^ T-cells and CD8^+^ T-cells (Hannon et al., 2021).

Genome-wide significant associations with a novel deletion downstream of *KLF14* were detected in 40 of the 86 DEGs that we screened in the eGWAS. A number of which were associated with natural killer (NK) cells, where major dysfunctions have been linked with functioning impairment correlated with psychotic, manic, and depressive symptoms in subsequently diagnosed patients with schizophrenia and bi-polar disorder (Tarantino et al., 2021). Transcription factor binding sites for KLF14 were significantly enriched in those 40 genes (Figure 3B). Hence, we propose that *KLF14* is a candidate regulator for the expression of genes that are involved in the pathogenic development that leads to feather pecking behavior. 10 of the 40 potential KLF14 targets play a role in the immune system, more specifically in leukocytes. CD4 T cells belong to the generic term leukocytes and have recently been shown to be essential for healthy development from the fetal to the adult brain in mice and humans (Pasciuto et al., 2020). Single-cell sequencing revealed that in the absence of murine CD4 T cells, resident microglia remained suspended between the fetal and adult states. This maturation defect resulted in excess immature neuronal synapses and behavioral abnormalities. The authors proposed that CD4 T cells play a so far neglected role in the development and evolution of the neurological system. An involvement of the immune system in feather pecking behavior has been proposed in numerous studies (Parmentier et al., 2009; Mashimo et al., 2017; Gandal et al., 2018; Sneeboer et al., 2019; van der Eijk et al., 2019; Falker-Gieske et al., 2020b; Falker-Gieske et al., 2021). Interestingly van der Eijk et al. showed that a HFP line expressed significantly lower amounts of CD4, CD4^+^ and CD8α+ T cells in comparison to an LFP line (van der Eijk et al., 2019). This would mirror results in human studies that also showed decreased proportions of natural killer cells, CD4^+^ T-cells and CD8^+^ T-cells in psychosis-associated patients (Hannon et al., 2021).

Although these analyses have been conducted in adult chickens it is fair to assume that lower T cell counts are also present during the embryonic development of HFP. The tissue expression of *KLF14* is by far highest in human placenta, 4-fold higher compared to skin (Fagerberg et al., 2014). As such, we submit a possible model in which a deletion downstream of the transcription factor *KLF14* has a negative impact on the level of T cells in the developing brain of HFP chickens, leading to developmental and behavioral abnormalities. As the evidence of the GABAergic systems involvement mounts, we currently believe that a lack of CD4 T cells and GABA receptors are major contributors of the propensity to perform feather pecking behavior in laying hens. Using transcriptional and computational analysis brings its own limits to the outcome of this study. Here we have put forward a solid hypothesis for the role of *KLF14*. It is however important, to understand that without further practical analysis this cannot be proven. Through further quantification of CD4 T cells and GABA receptors in HFP and LFP animals, throughout multiple stages of development, it could be possible to elucidate the role that *KLF14* plays in FP. Further analysis of other neurological disorder looking at the role *KLF14* plays there, could also open the door for the use of HFP animals as a model to study other neurological disorders.

## Conclusion

We propose that a deletion downstream of the transcription factor *KLF14* has a negative impact on the level of T cells in the developing brain of FP chickens, which leads to developmental and behavioral abnormalities. This reduction of CD4 T cells and GABA receptors are a major cause for the propensity of laying hens to perform feather pecking behavior. As such, *KLF14* is a clear candidate key regulator for the expression of genes involved in the pathogenic development. By further elucidating the regulatory pathways involved in FP we hope to take significant steps forward in explaining and understanding other mental disorders, not just in chickens.

## Data Availability

The datasets presented in this study can be found in online repositories. The names of the repository/repositories and accession number(s) can be found below: https://www.ebi.ac.uk/ena, PRJNA664592 https://www.ebi.ac.uk/ena, PRJNA656654.
