# Supplementary material for: eQTL analysis of laying hens divergently selected for feather pecking identifies KLF14 as a potential key regulator for this behavioral disorder
Source: Front Genet. 2022 Aug 17;13:969752. doi: 10.3389/fgene.2022.969752 (PMC9428588; doi:10.3389/fgene.2022.969752)
Supplement: Supplementary file 4 [file DataSheet1.PDF]

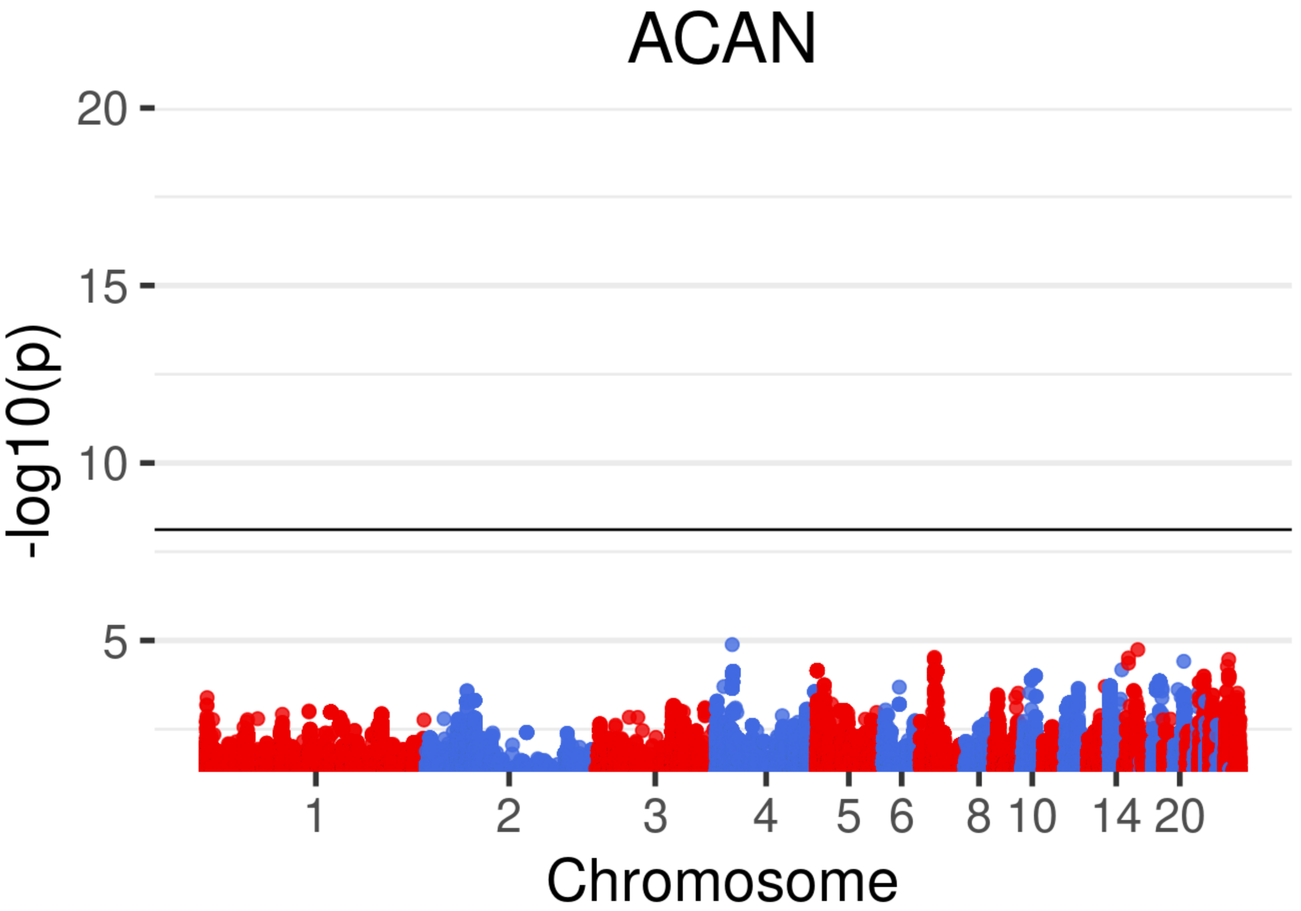
ACAN
20
15
10
5
-log10(p)
1
2
3
4
5
6
8
10
14
20
Chromosome

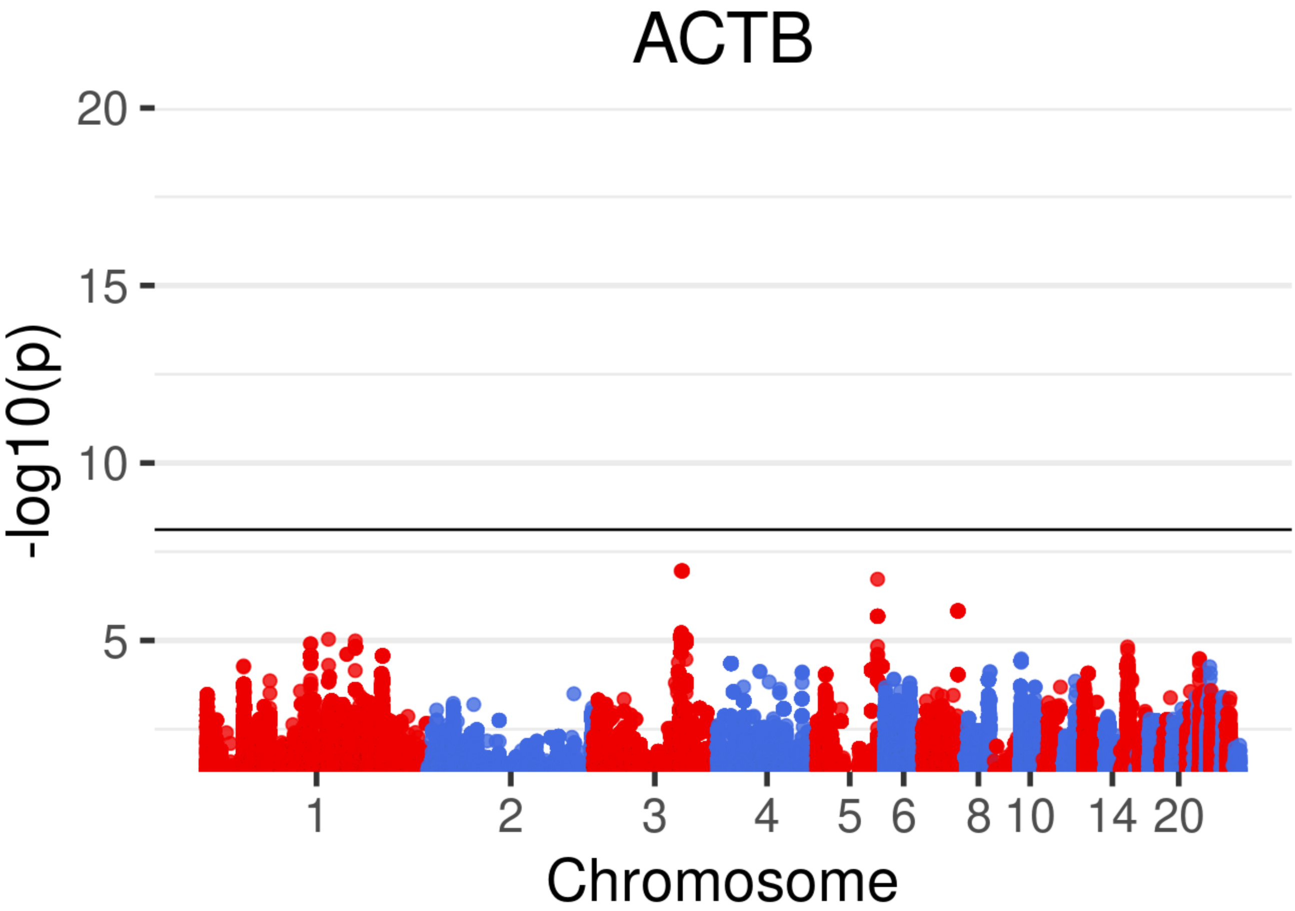
ACTB
20
15
10
5
-log10(p)
1
2
3
4
5
6
8
10
14
20
Chromosome

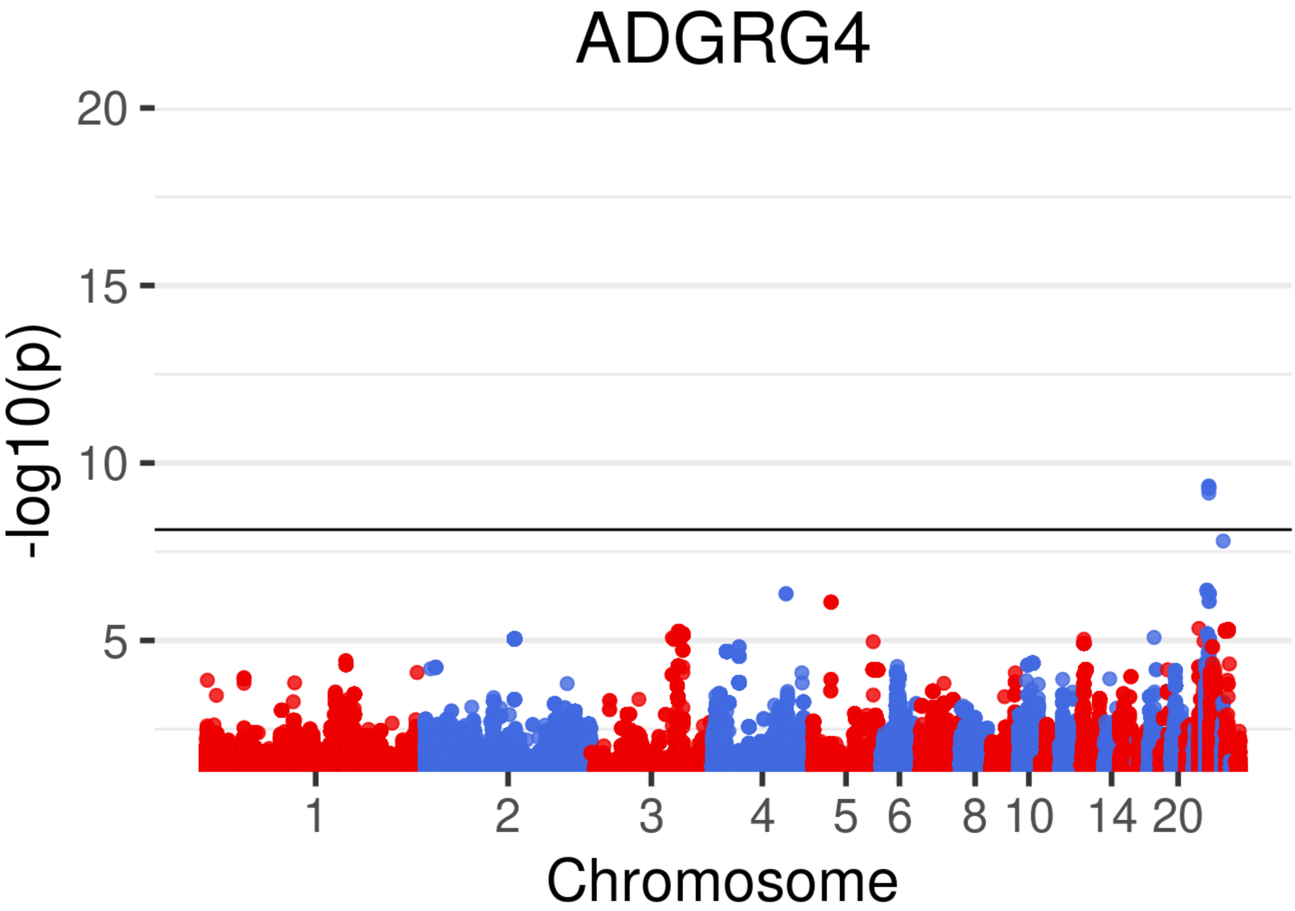

ADGRG4
20
15
10
5
-log10(p)
1
2
3
4
5
6
8
10
14
20
Chromosome

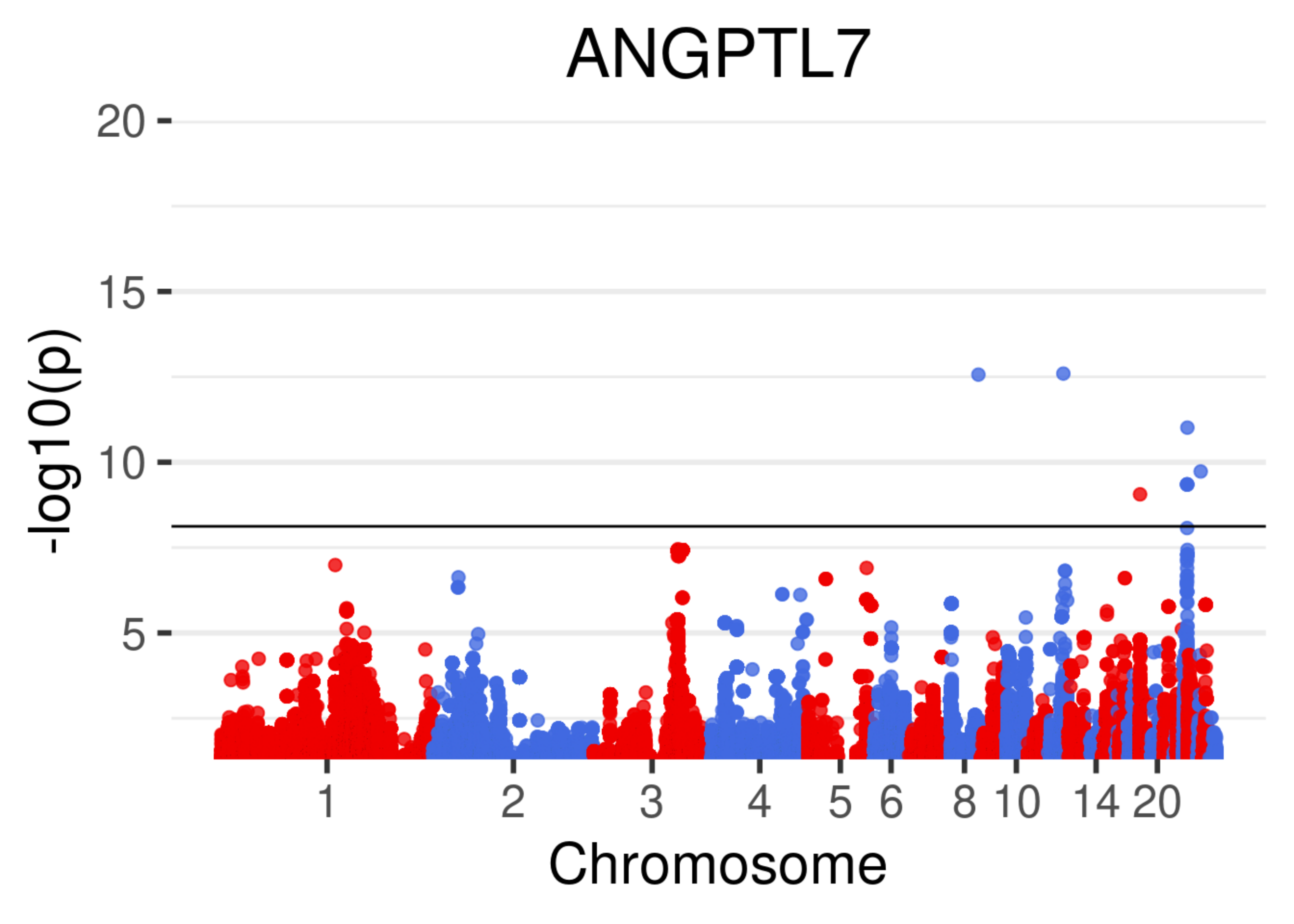
ANGPTL7
-log10(p)
20
15
10
5
1
2
3
4
5
6
8
10
14
20
Chromosome

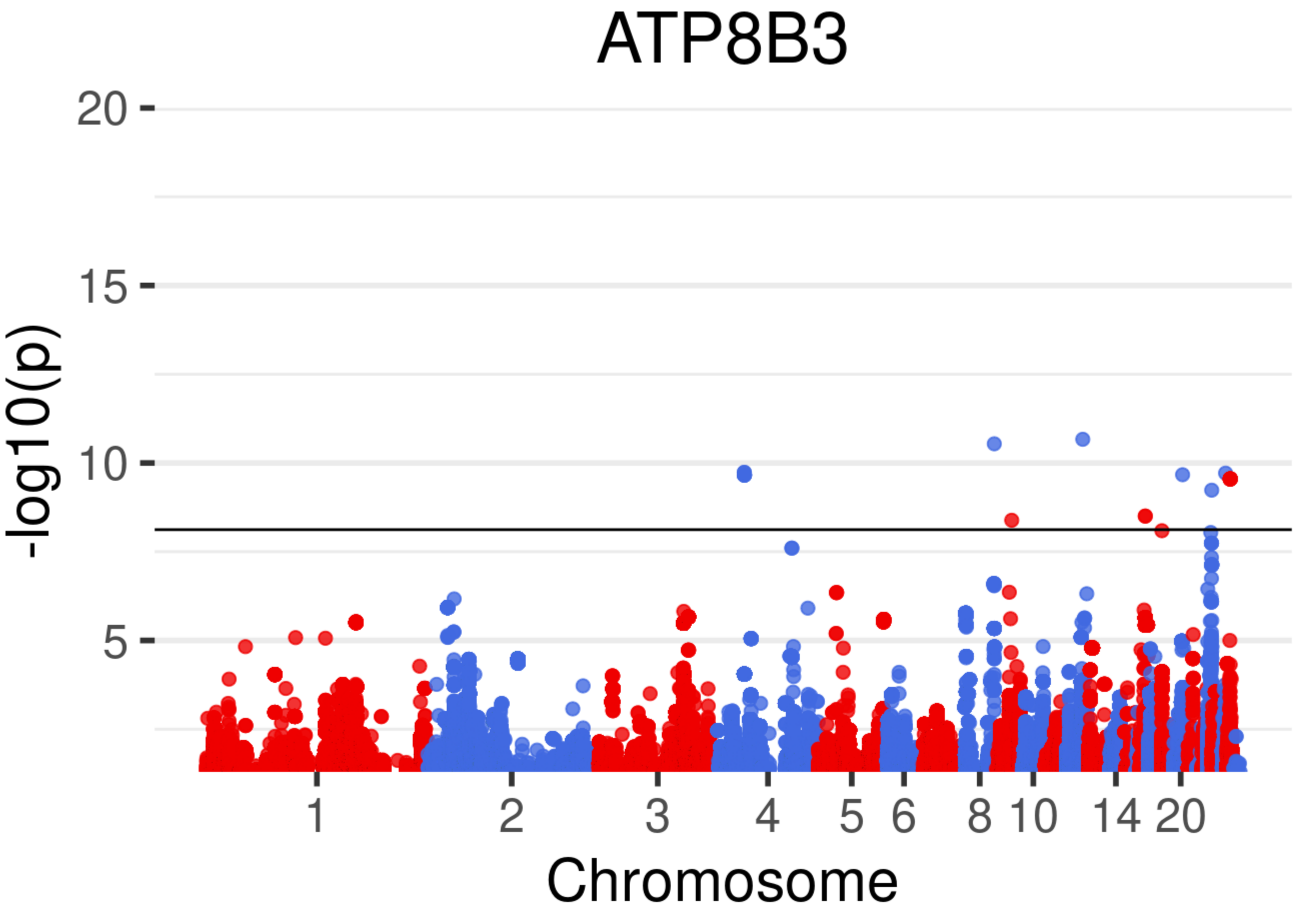
ATP8B3
20
15
10
5
-log10(p)
1
2
3
4
5
6
8
10
14
20
Chromosome

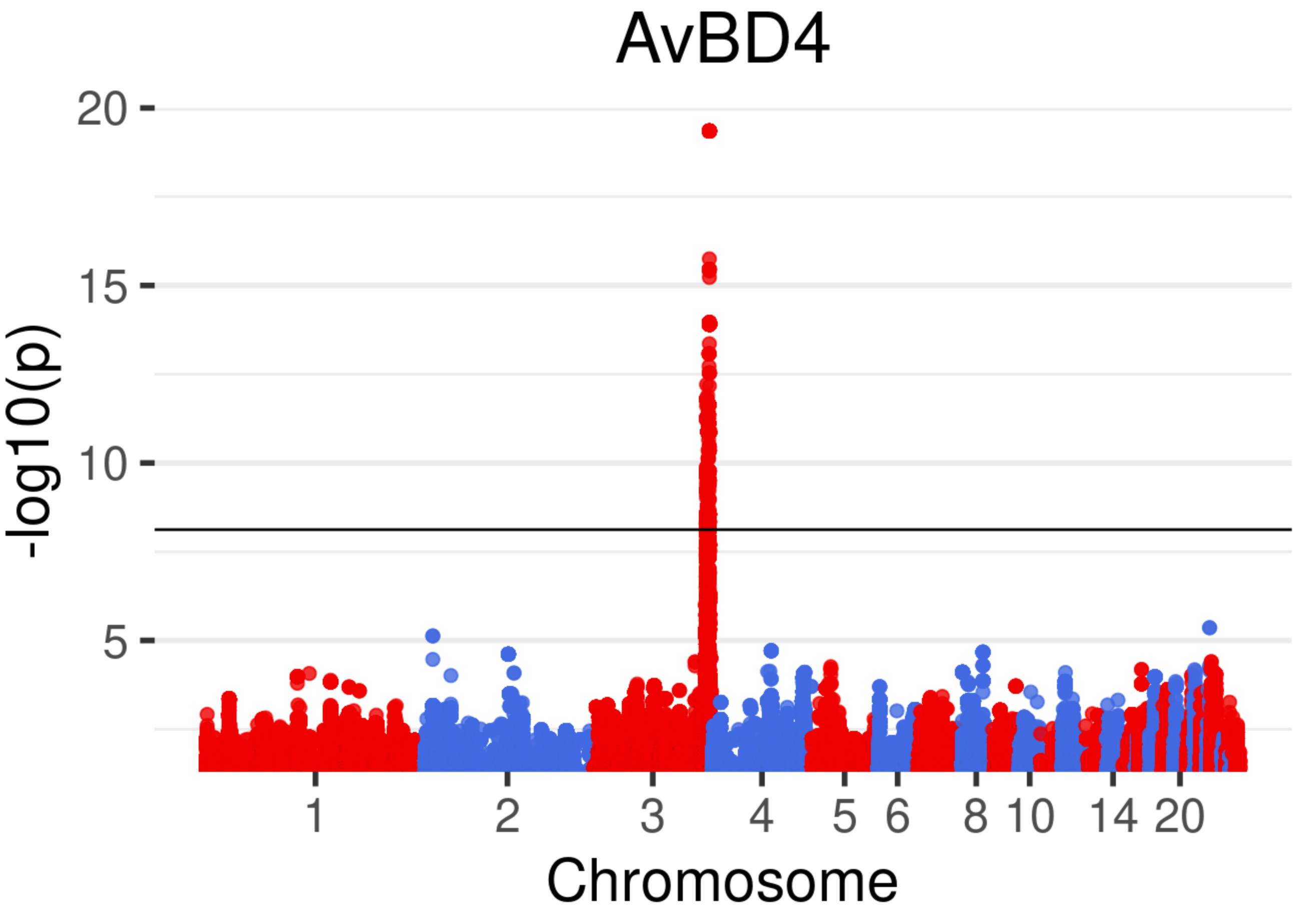

AvBD4
20
15
10
5
-log10(p)
1
2
3
4
5
6
8
10
14
20
Chromosome

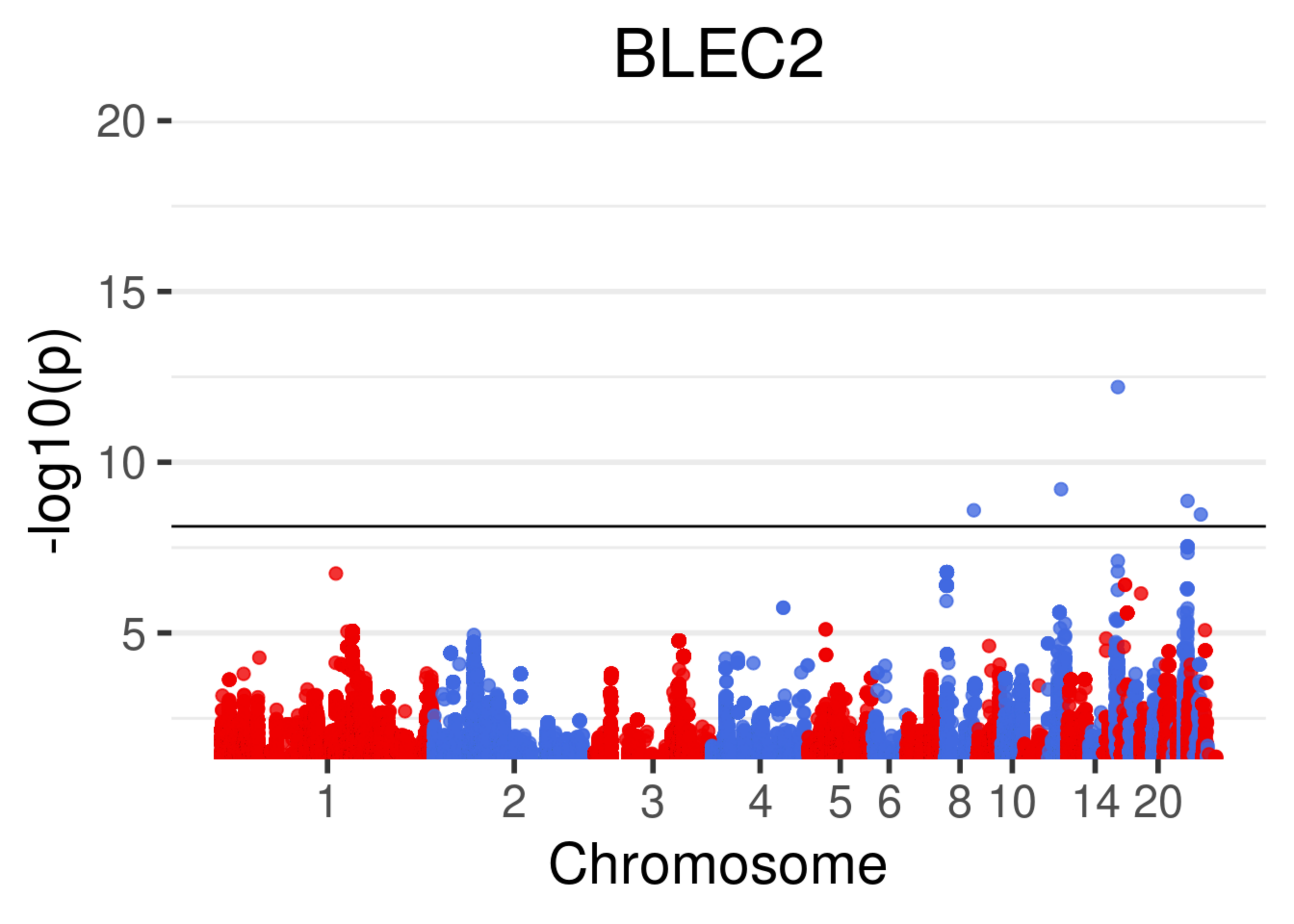
BLEC2
-log10(p)
20
15
10
5
1
2
3
4
5
6
8
10
14
20
Chromosome

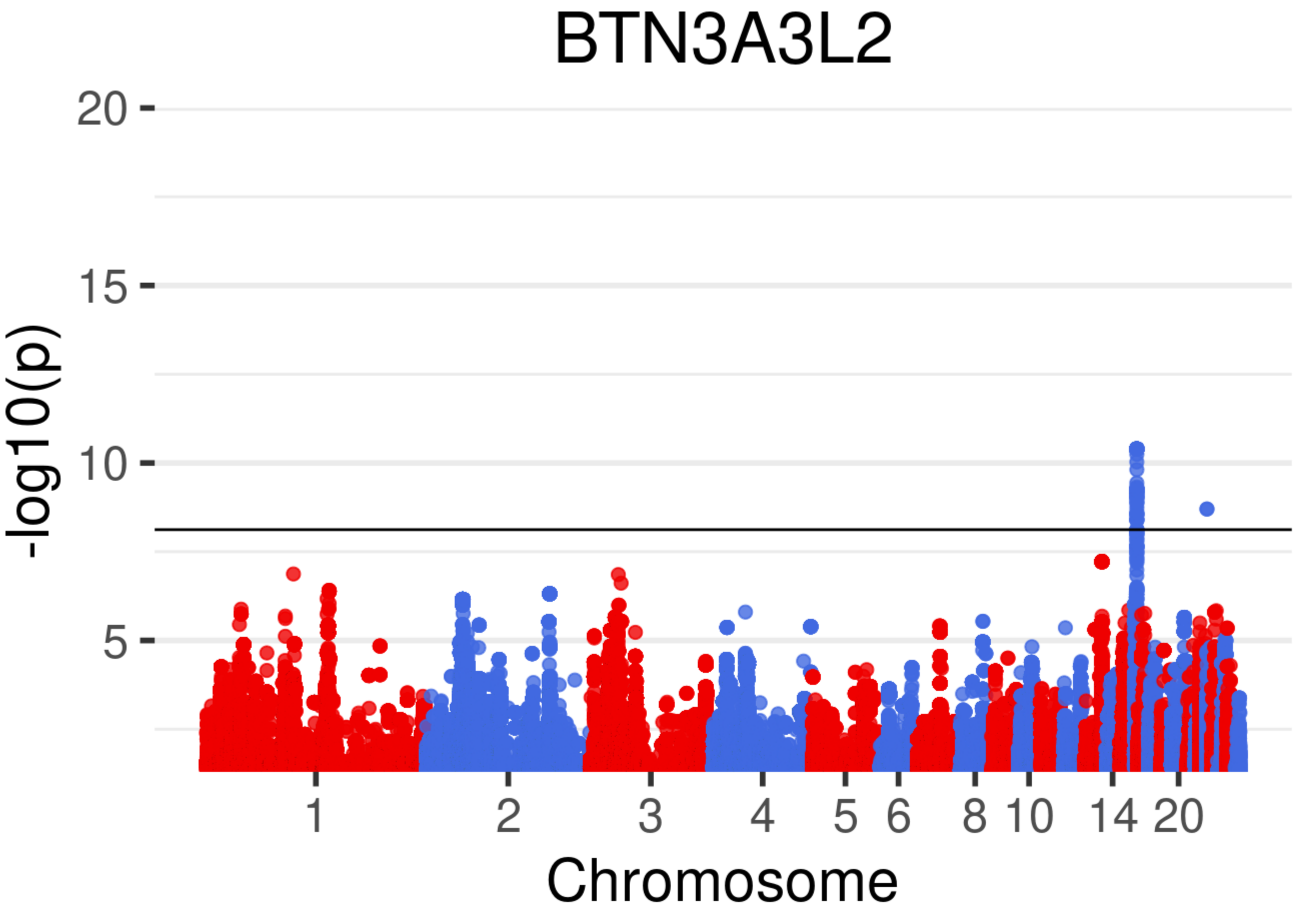

BTN3A3L2
-log10(p)
20
15
10
5
Chromosome
1
2
3
4
5
6
8
10
14
20

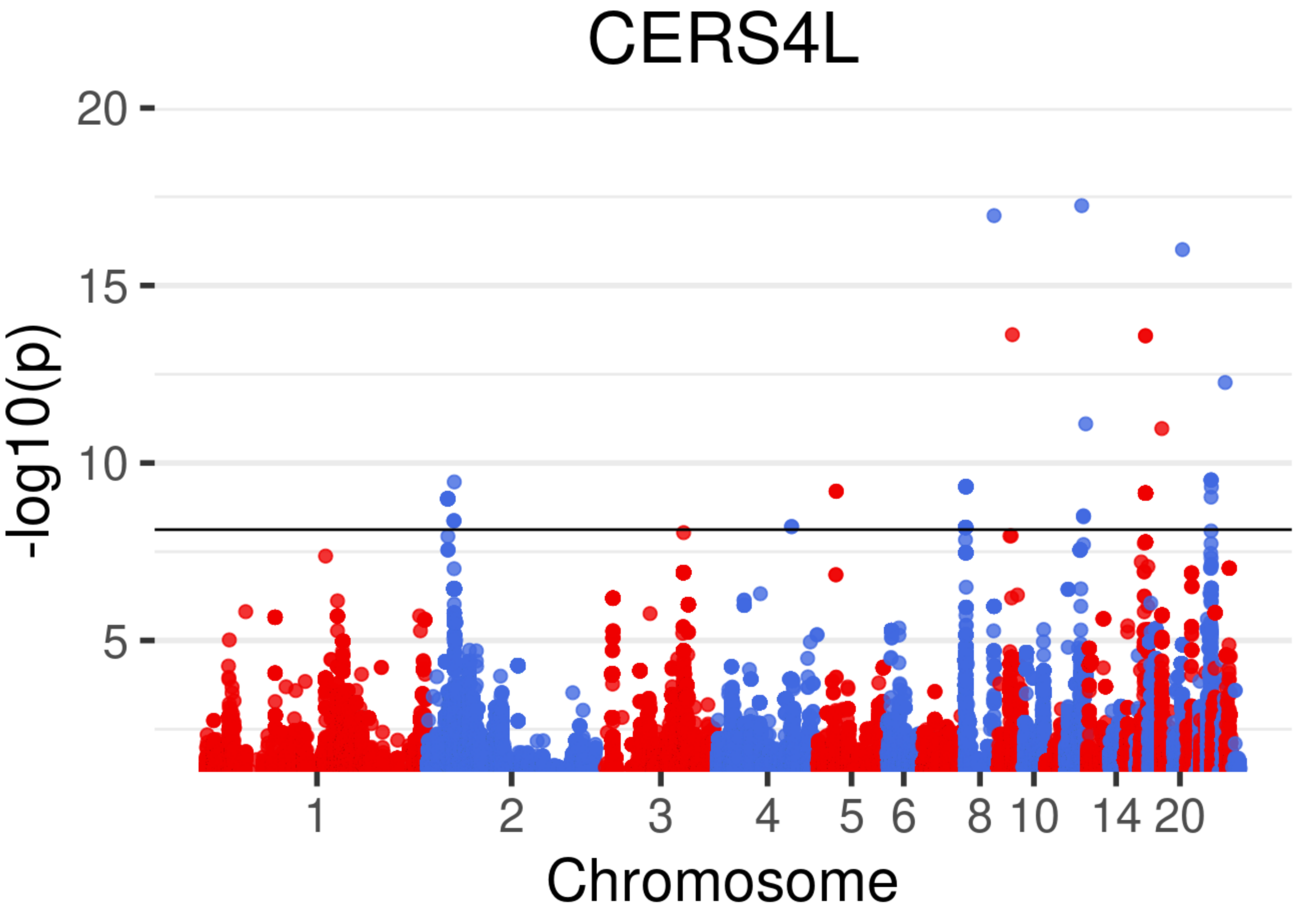
CERS4L
20
15
10
5
-log10(p)
1
2
3
4
5
6
8
10
14
20
Chromosome

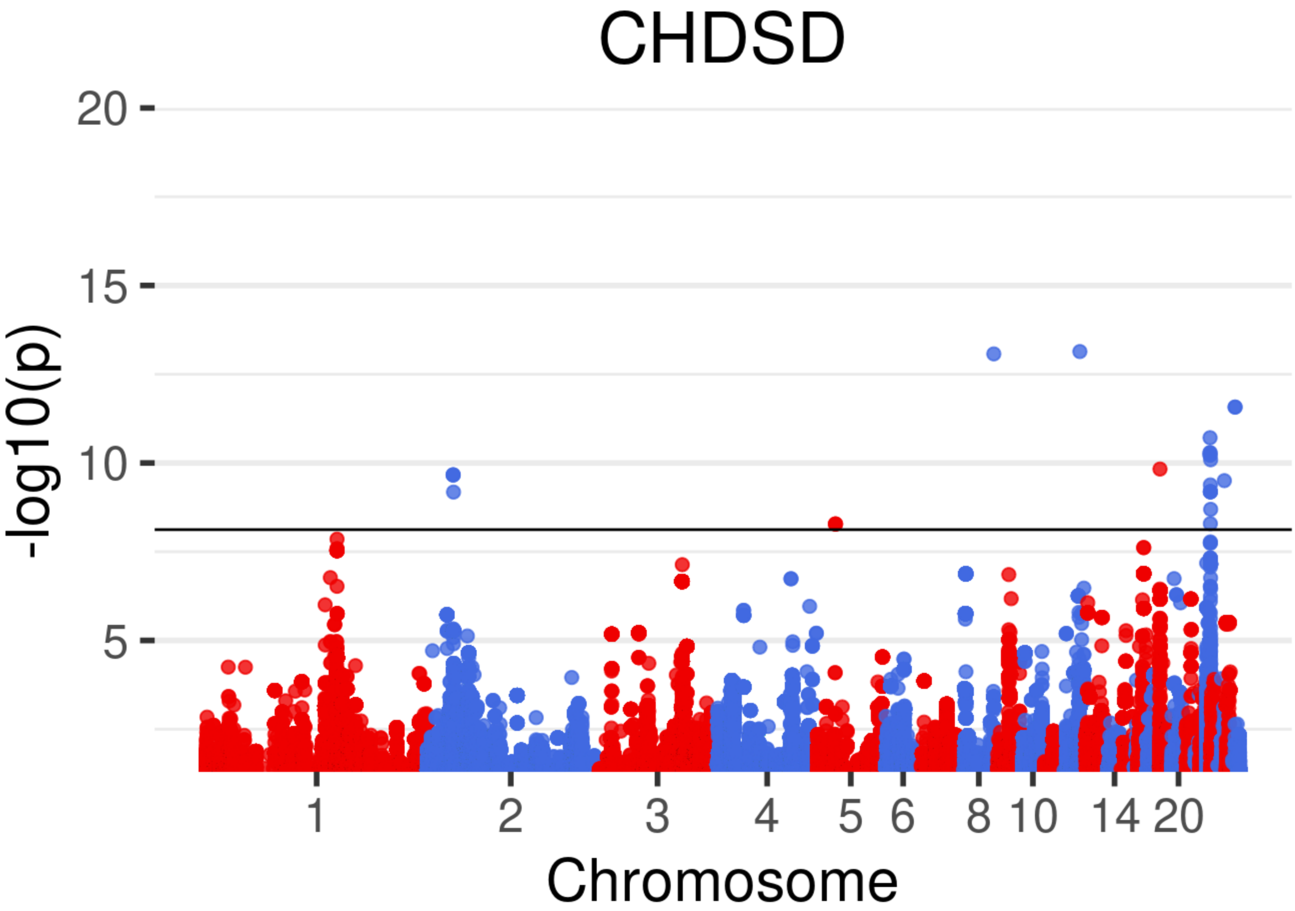
CHDSD
20
15
10
5
-log10(p)
1
2
3
4
5
6
8
10
14
20
Chromosome

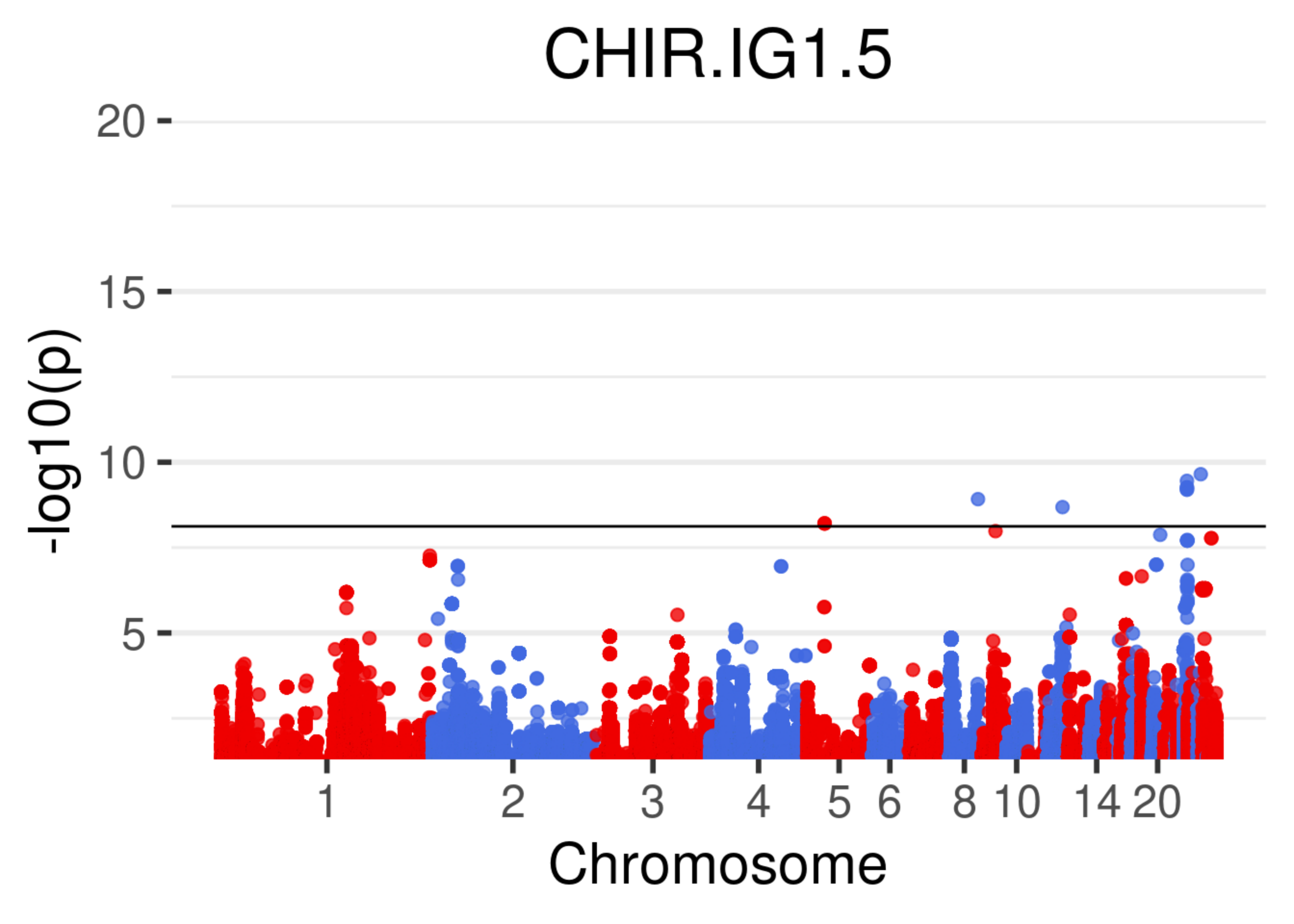
CHIR.IG1.5
20
15
10
5
-log10(p)
1
2
3
4
5
6
8
10
14
20
Chromosome

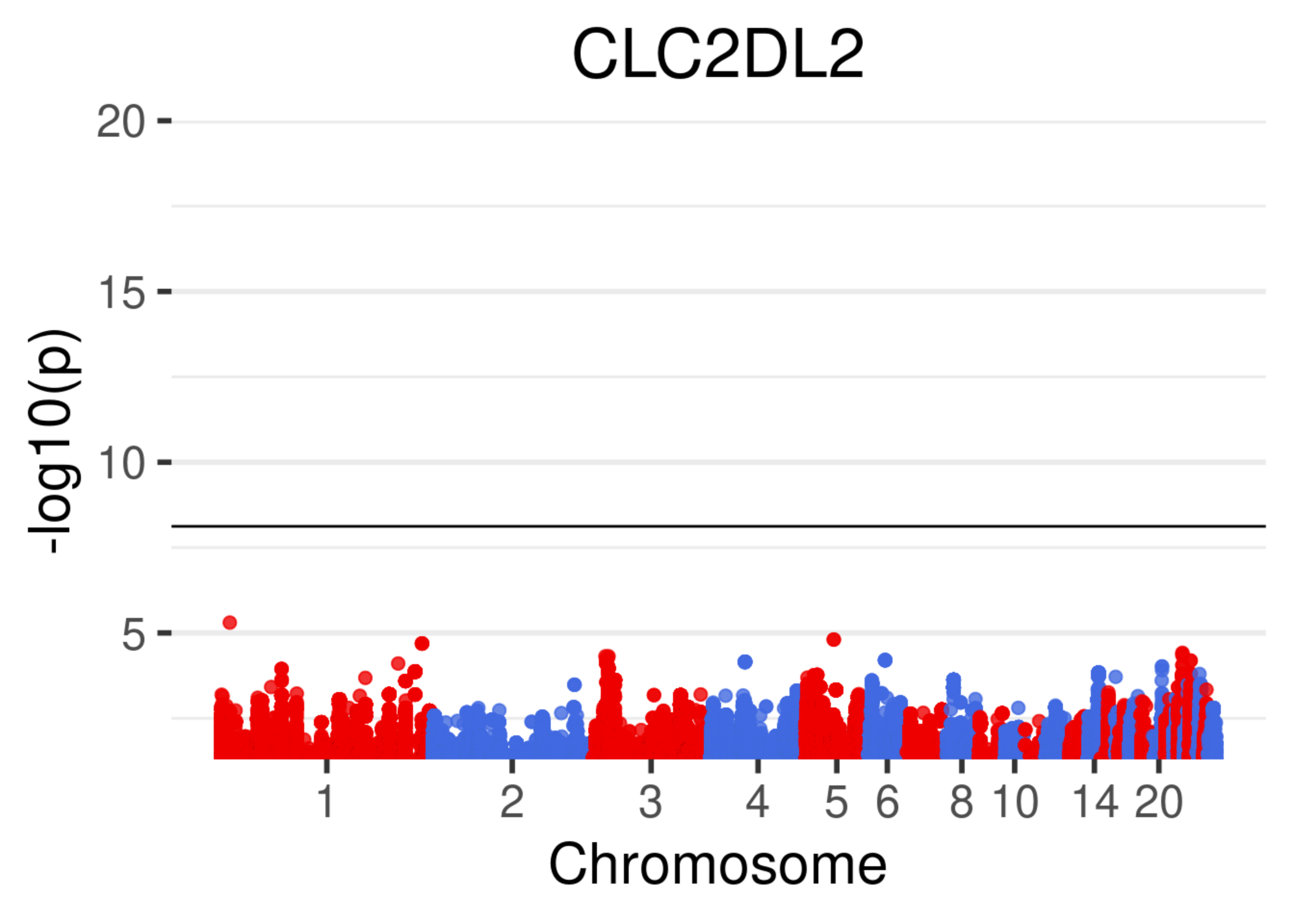

CLC2DL2
20
15
10
5
-log10(p)
1
2
3
4
5
6
8
10
14
20
Chromosome

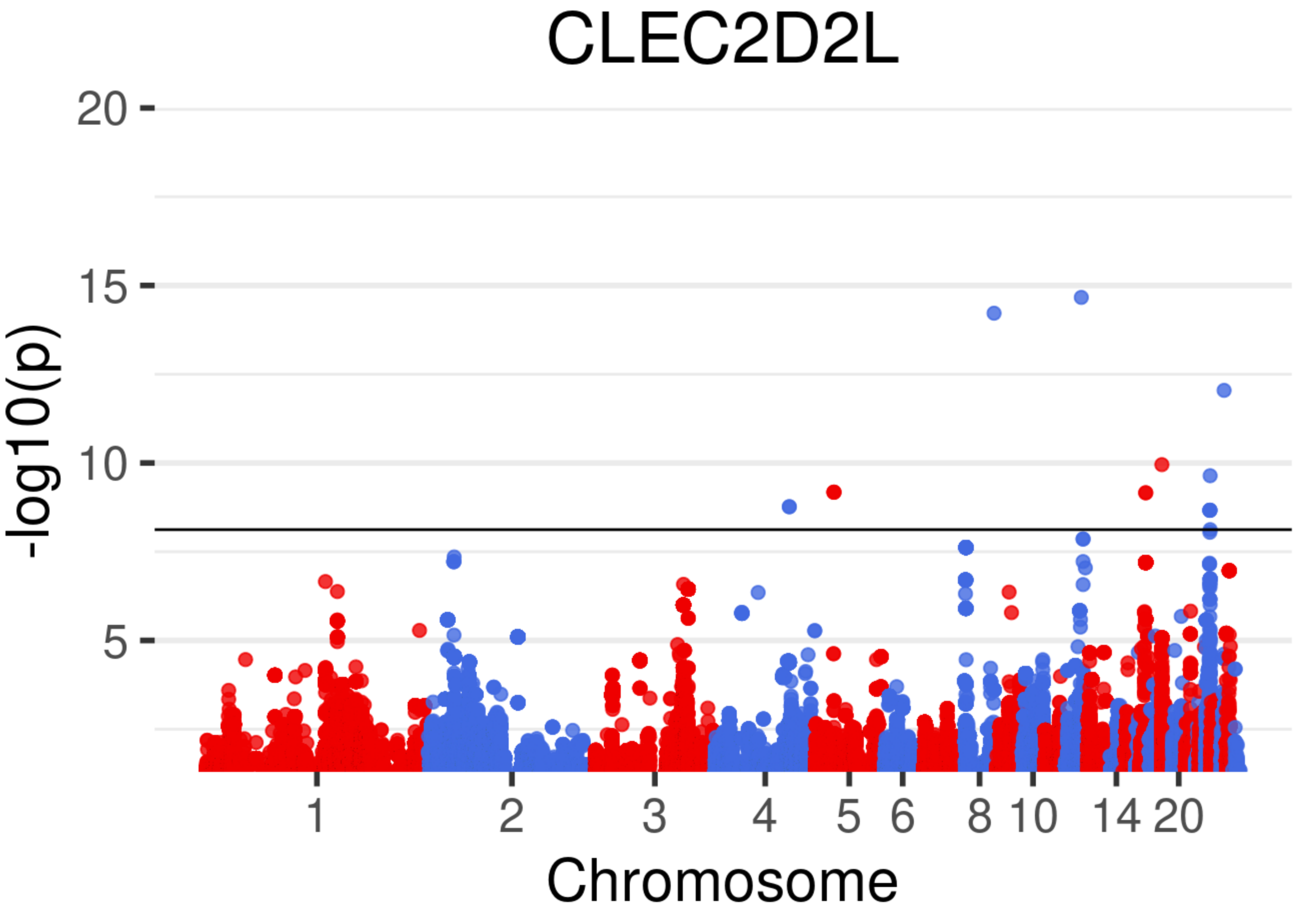
CLEC2D2L
20
15
10
5
-log10(p)
1
2
3
4
5
6
8
10
14
20
Chromosome

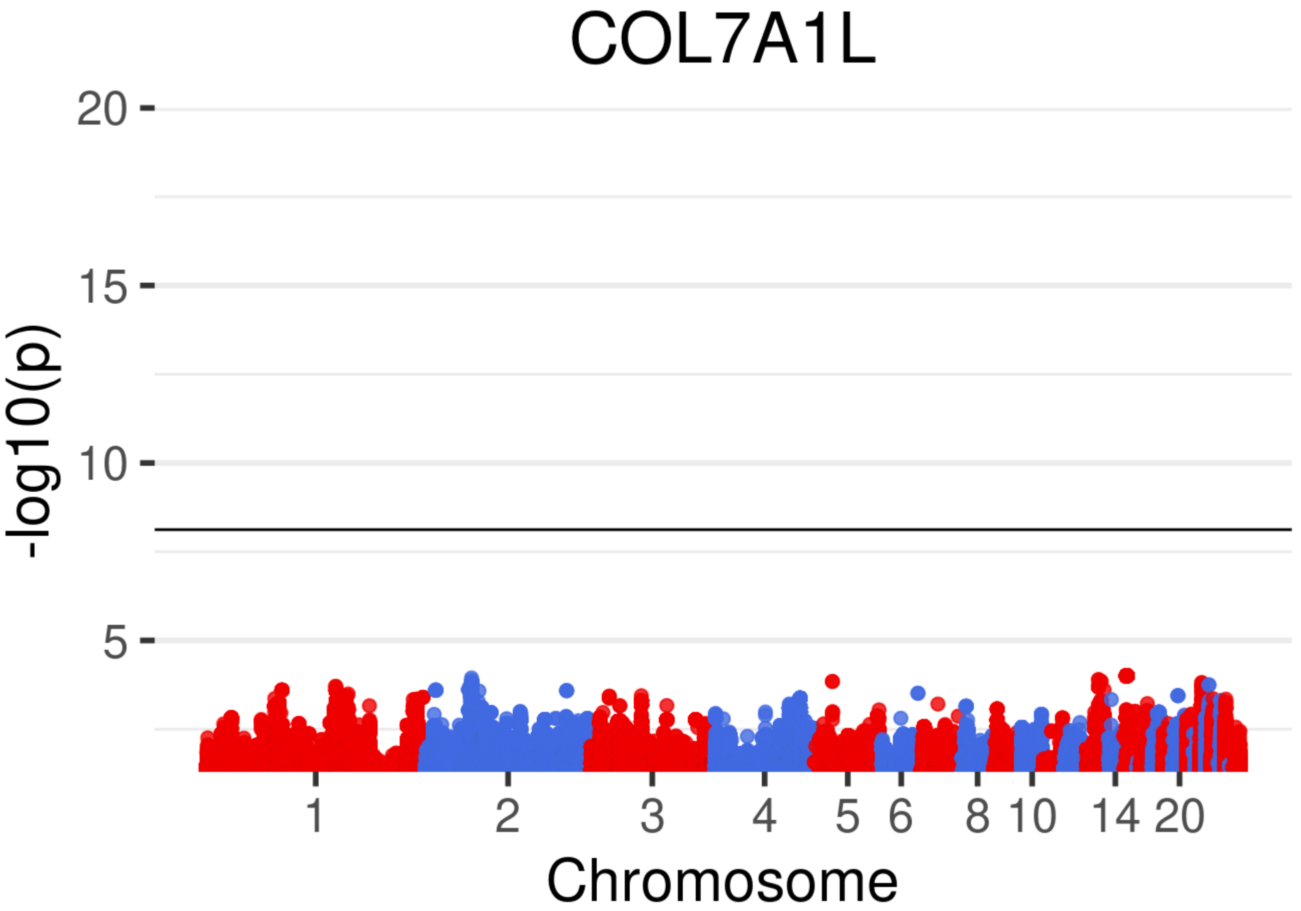

COL7A1L
20
15
10
5
-log10(p)
1
2
3
4
5
6
8
10
14
20
Chromosome

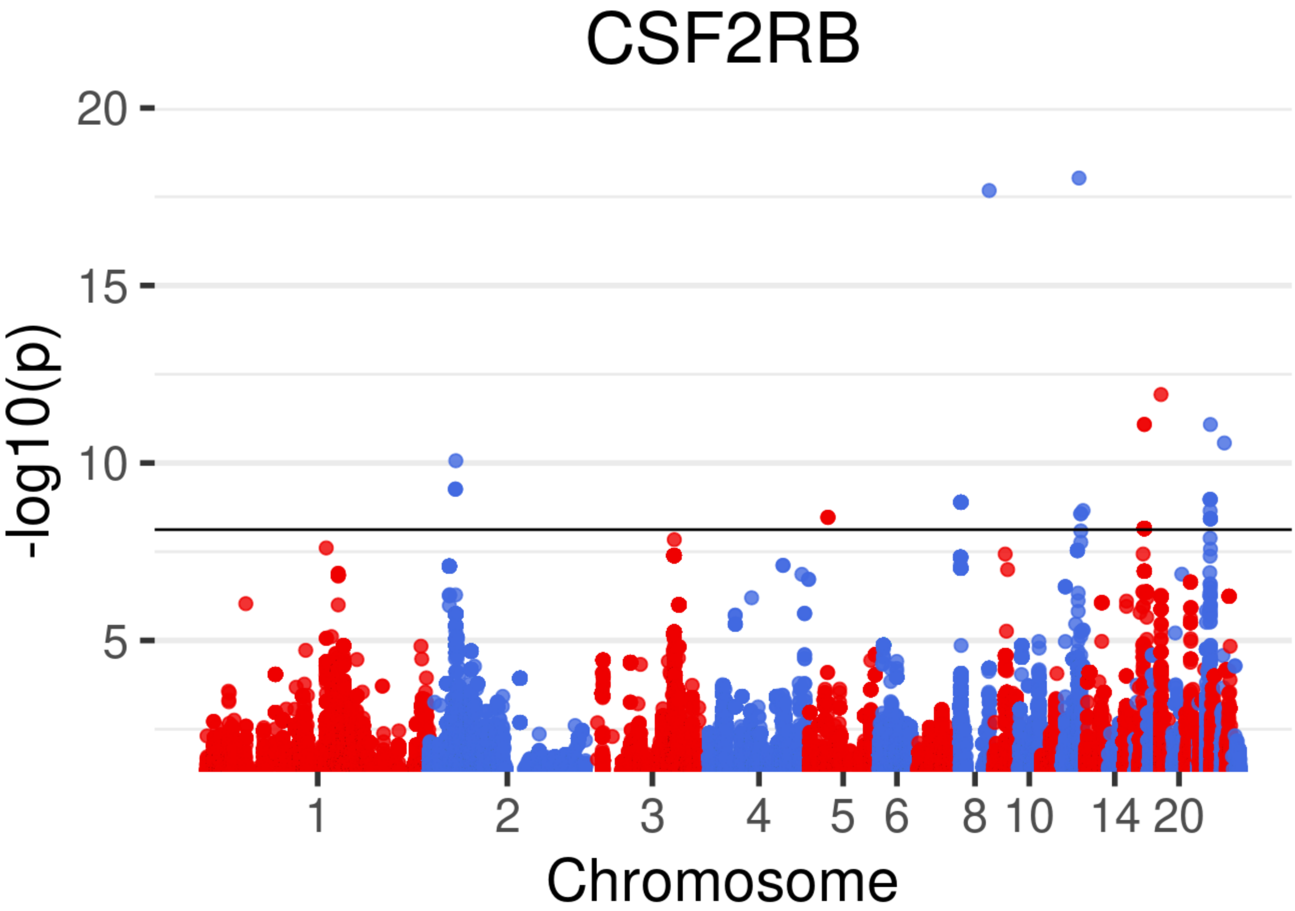

CSF2RB
-log10(p)
20
15
10
5
1
2
3
4
5
6
8
10
14
20
Chromosome

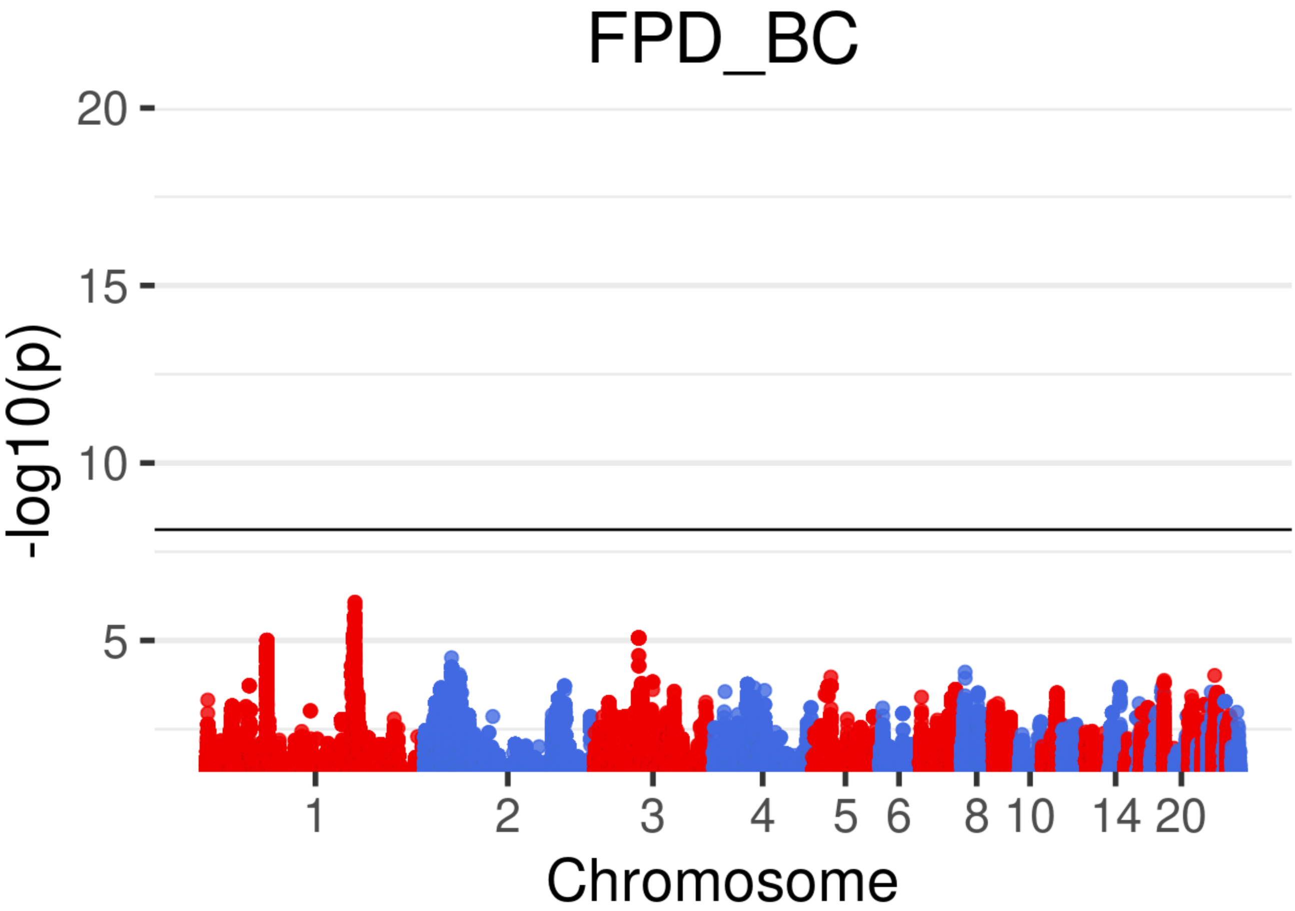

FPD_BC
20
15
10
5
-log10(p)
1
2
3
4
5
6
8
10
14
20
Chromosome

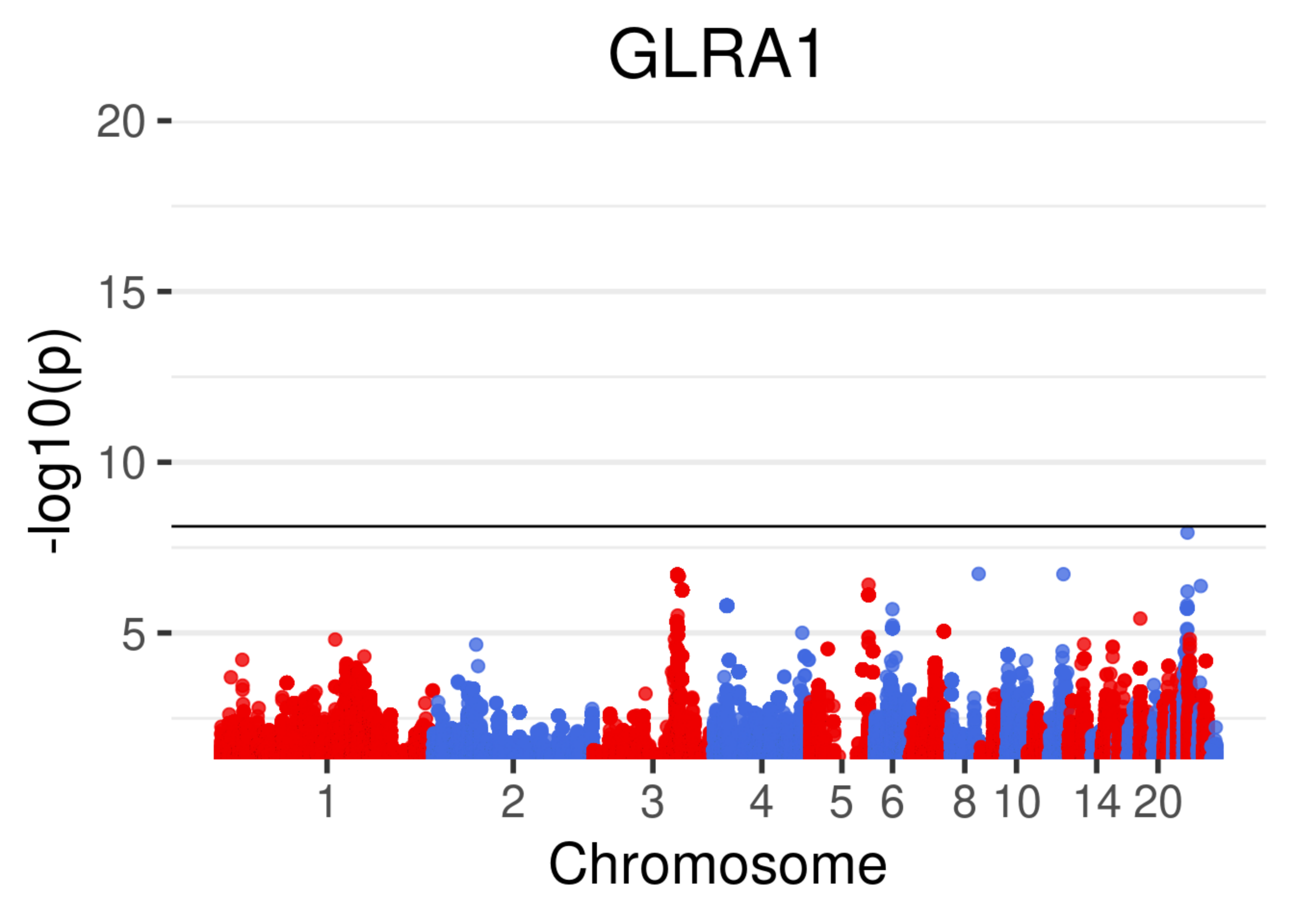
GLRA1
20
15
10
5
-log10(p)
1
2
3
4
5
6
8
10
14
20
Chromosome

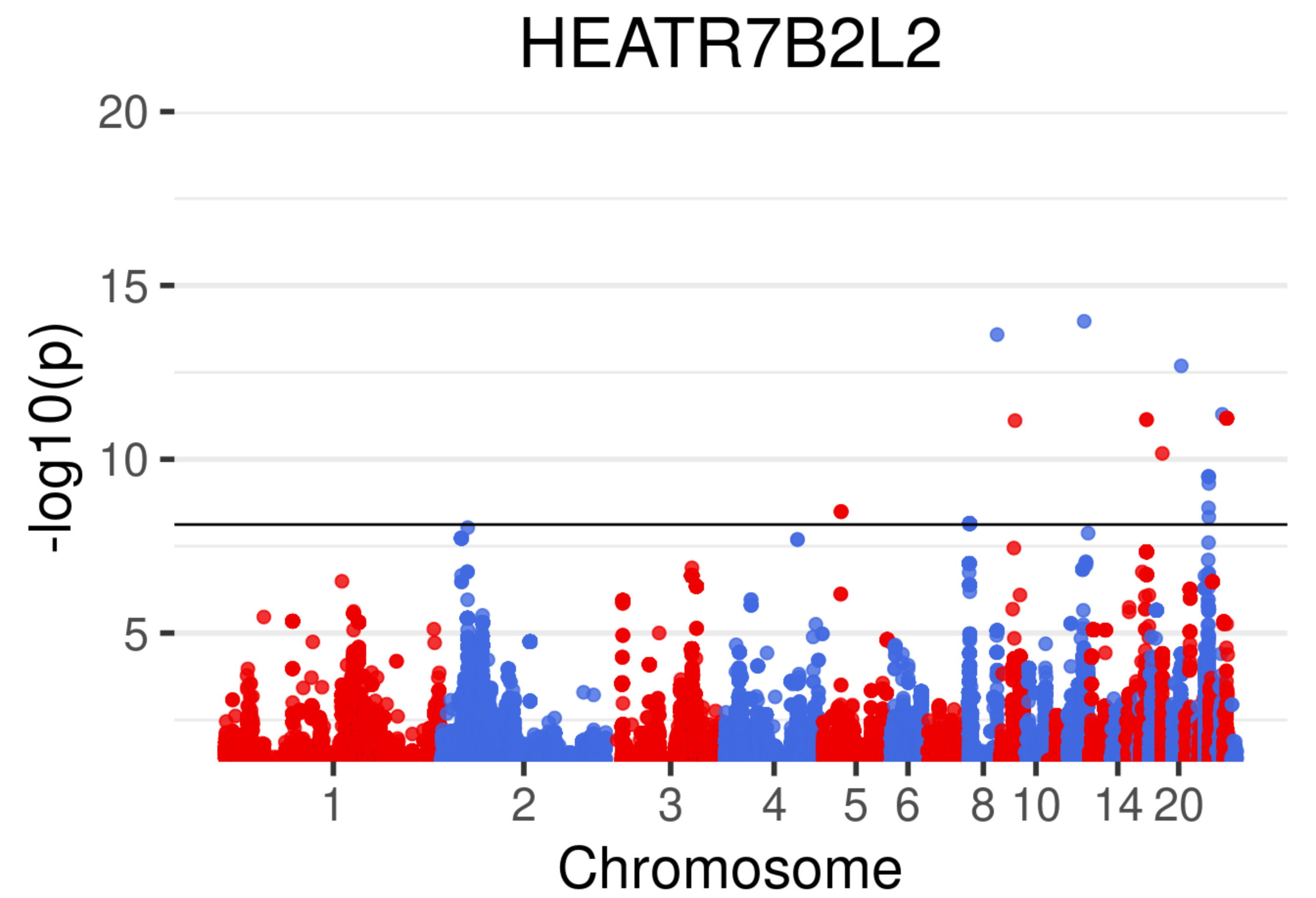
HEATR7B2L2
20
15
10
5
-log10(p)
1
2
3
4
5
6
8
10
14
20
Chromosome

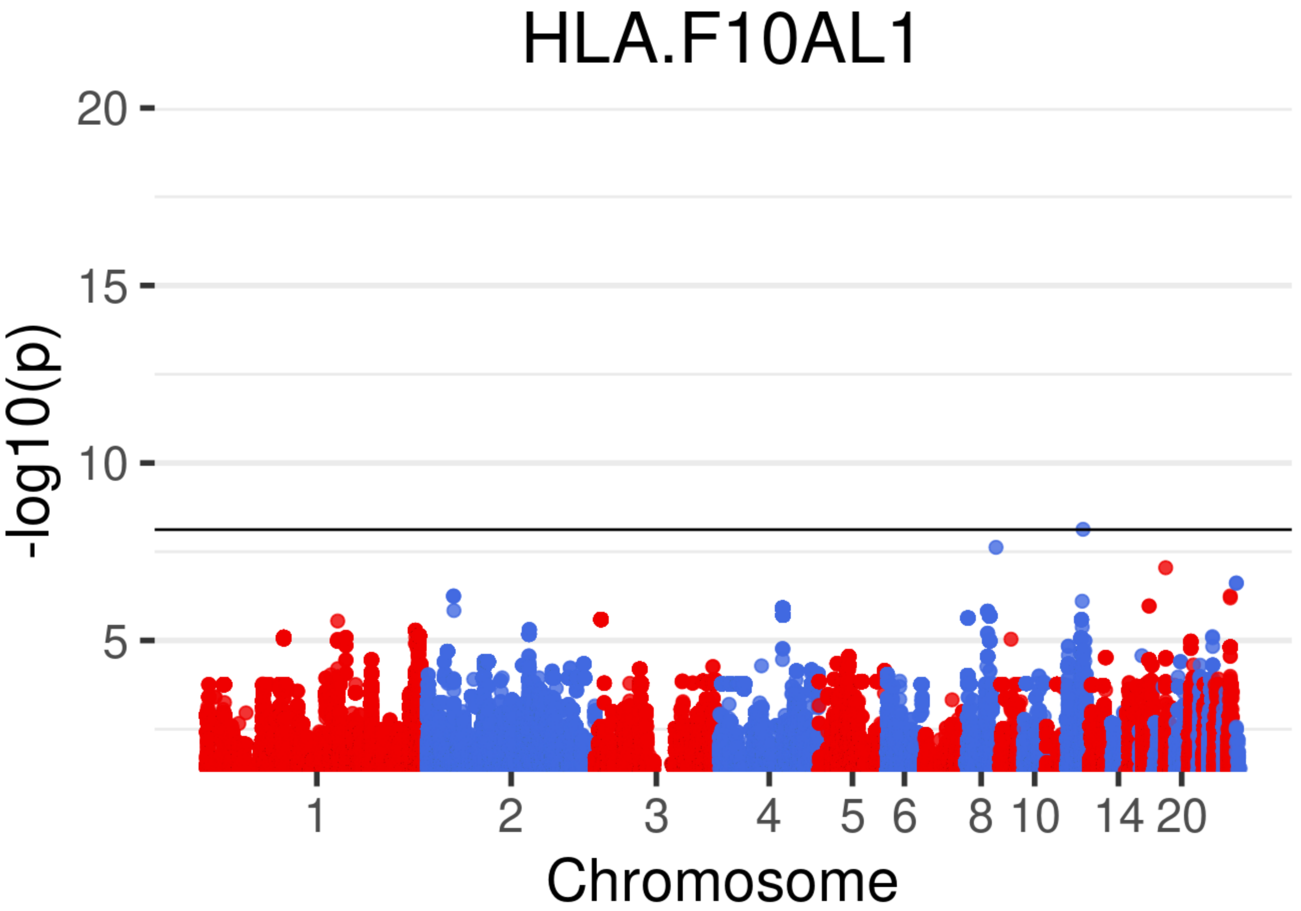
HLA.F10AL1
-log10(p)
20
15
10
5
1
2
3
4
5
6
8
10
14
20
Chromosome

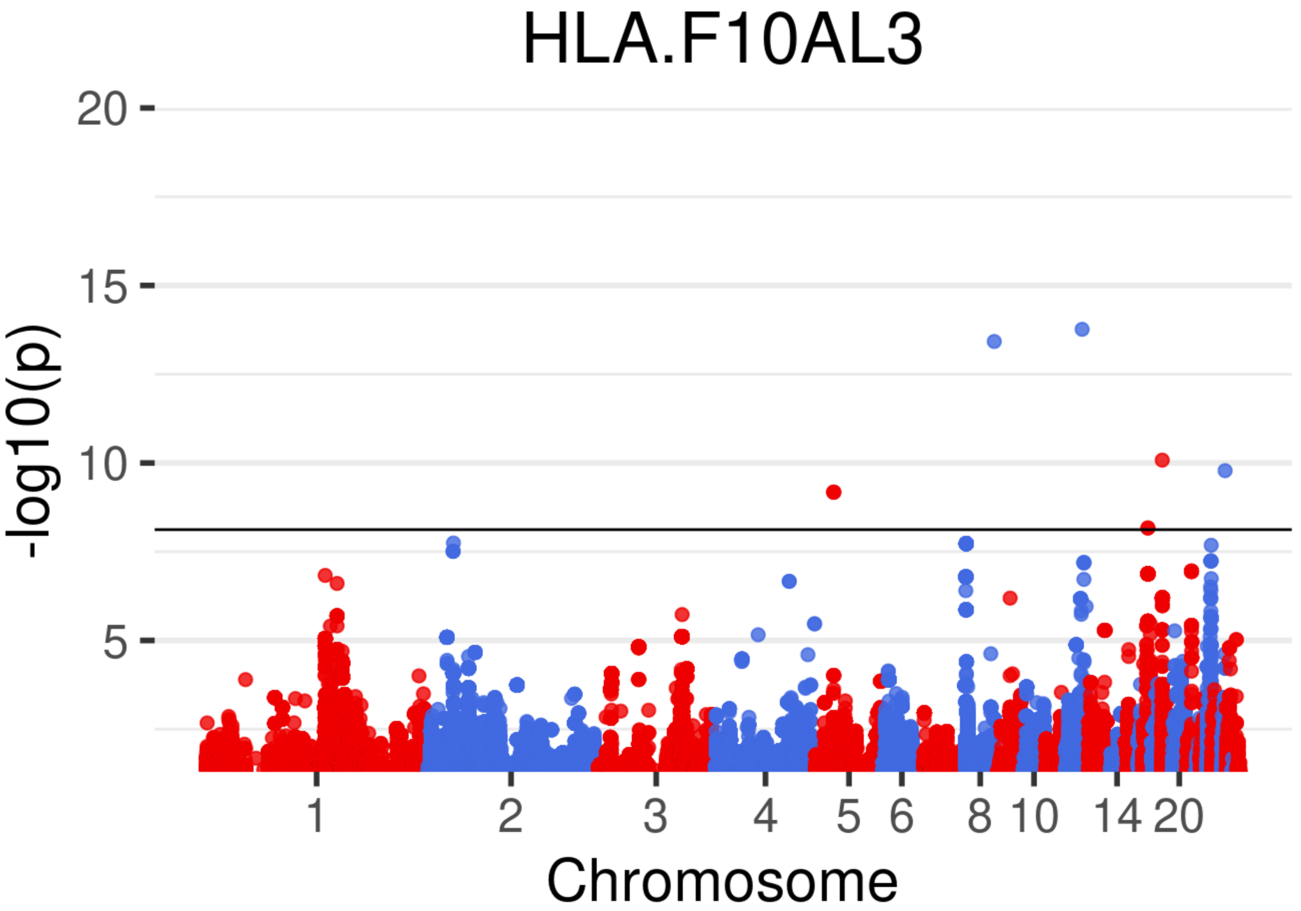
HLA.F10AL3
20
15
10
5
-log10(p)
1
2
3
4
5
6
8
10
14
20
Chromosome

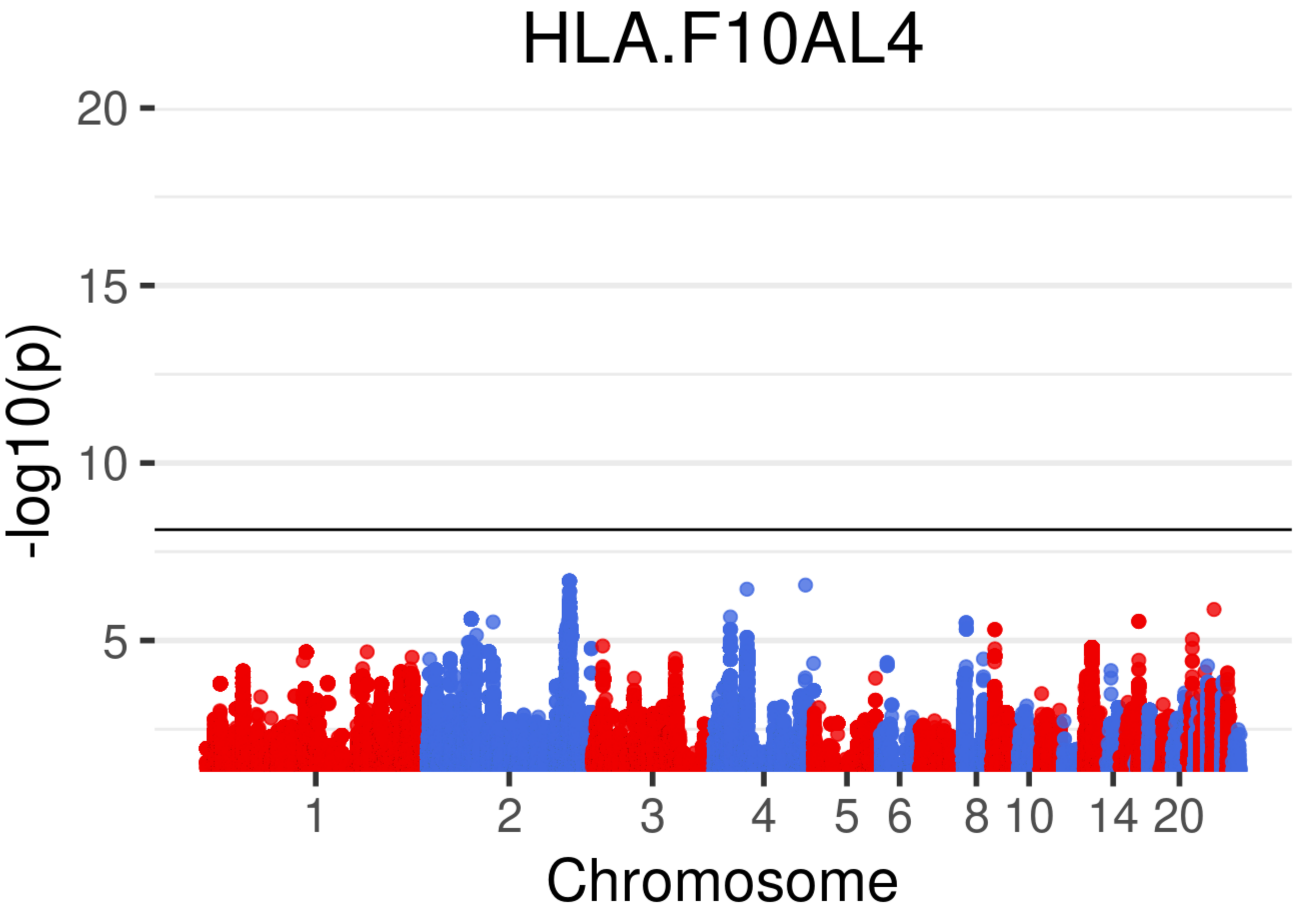

HLA.F10AL4
20
15
10
5
-log10(p)
1
2
3
4
5
6
8
10
14
20
Chromosome

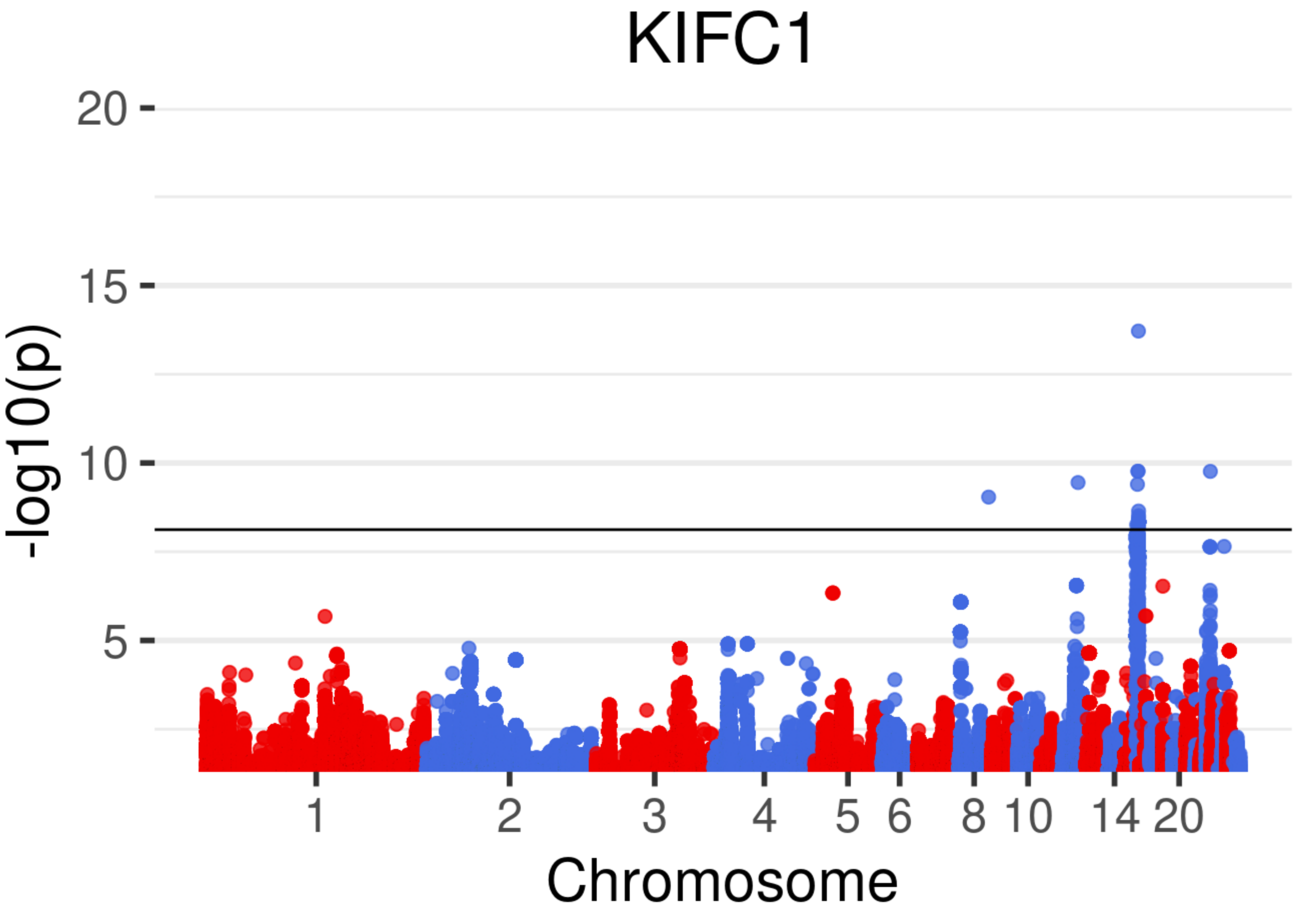
KIFC1
20
15
10
5
-log10(p)
1
2
3
4
5
6
8
10
14
20
Chromosome

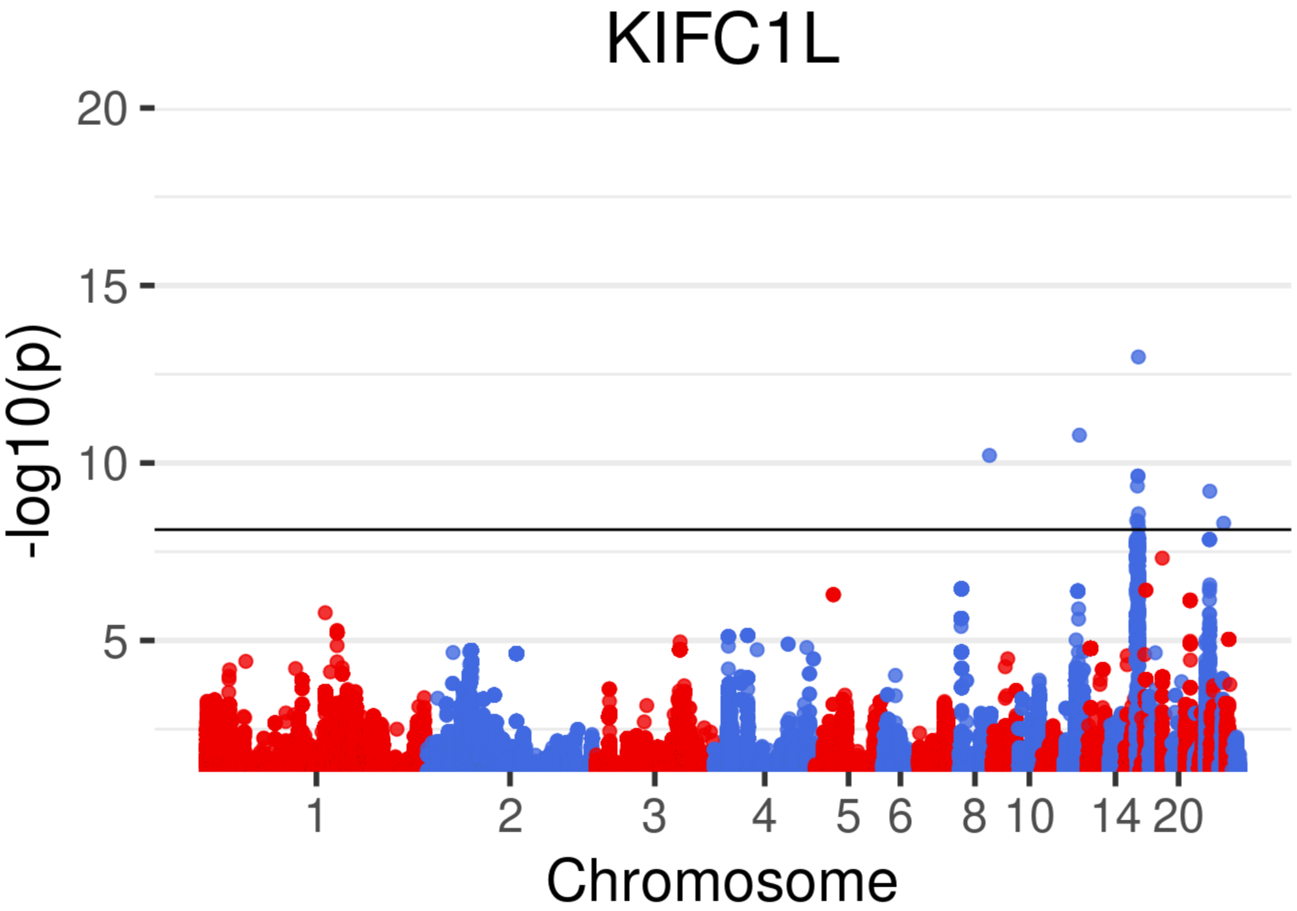

KIFC1L
-log10(p)
20
15
10
5
1
2
3
4
5
6
8
10
14
20
Chromosome

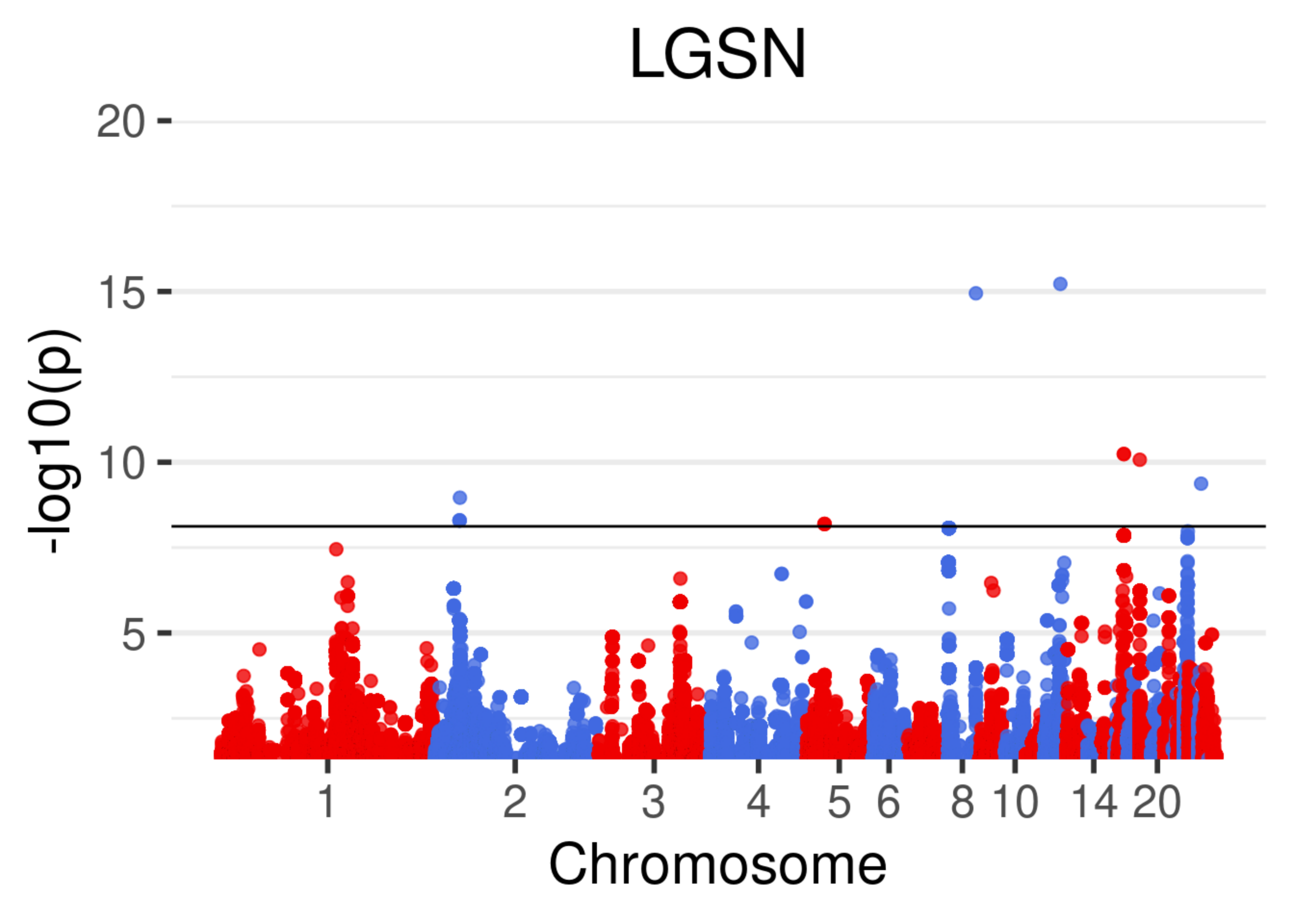

LGSN
20
15
10
5
-log10(p)
1
2
3
4
5
6
8
10
14
20
Chromosome

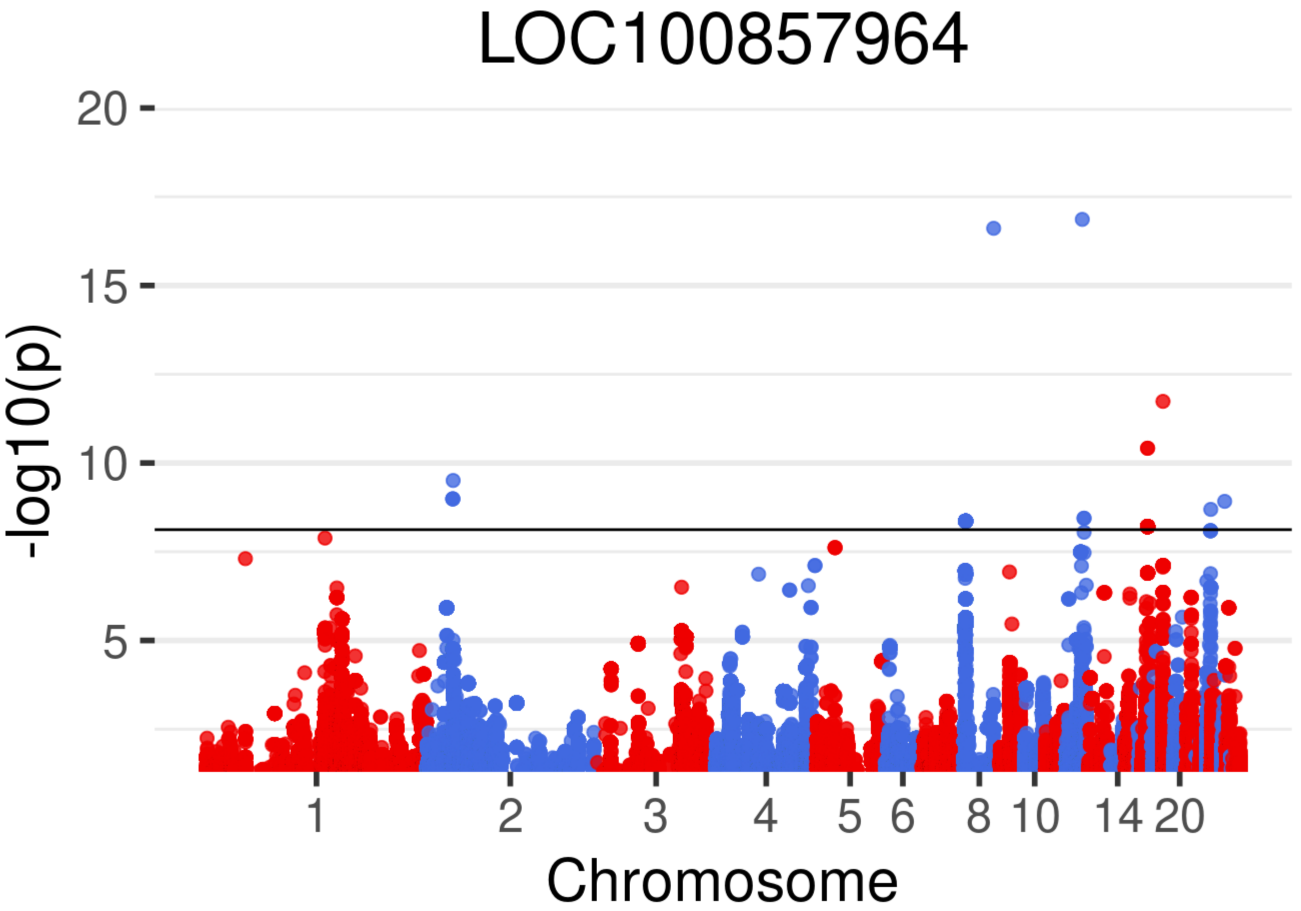
LOC100857964
20
15
10
5
-log10(p)
1
2
3
4
5
6
8
10
14
20
Chromosome

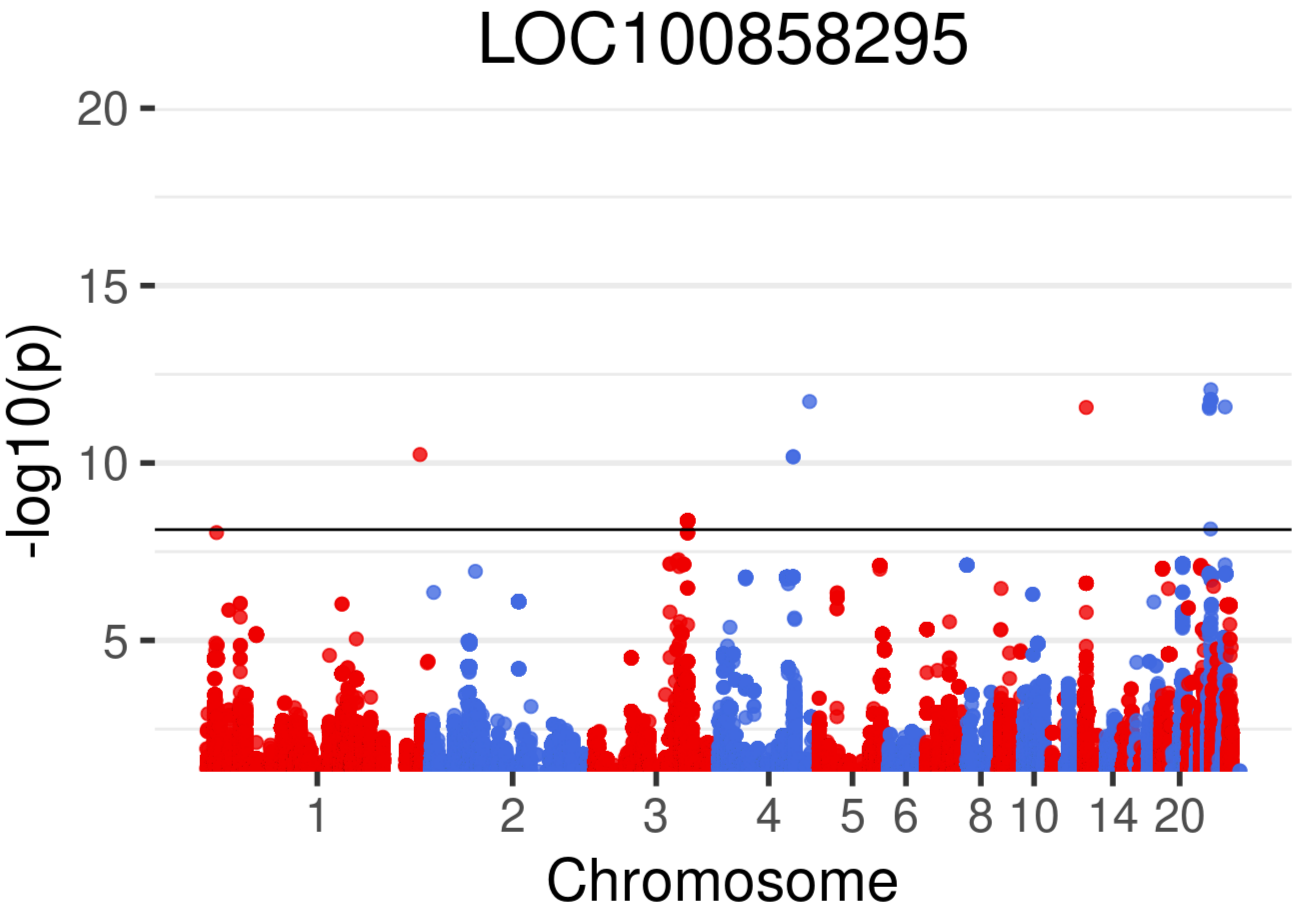
LOC100858295
20
15
10
5
-log10(p)
1
2
3
4
5
6
8
10
14
20
Chromosome

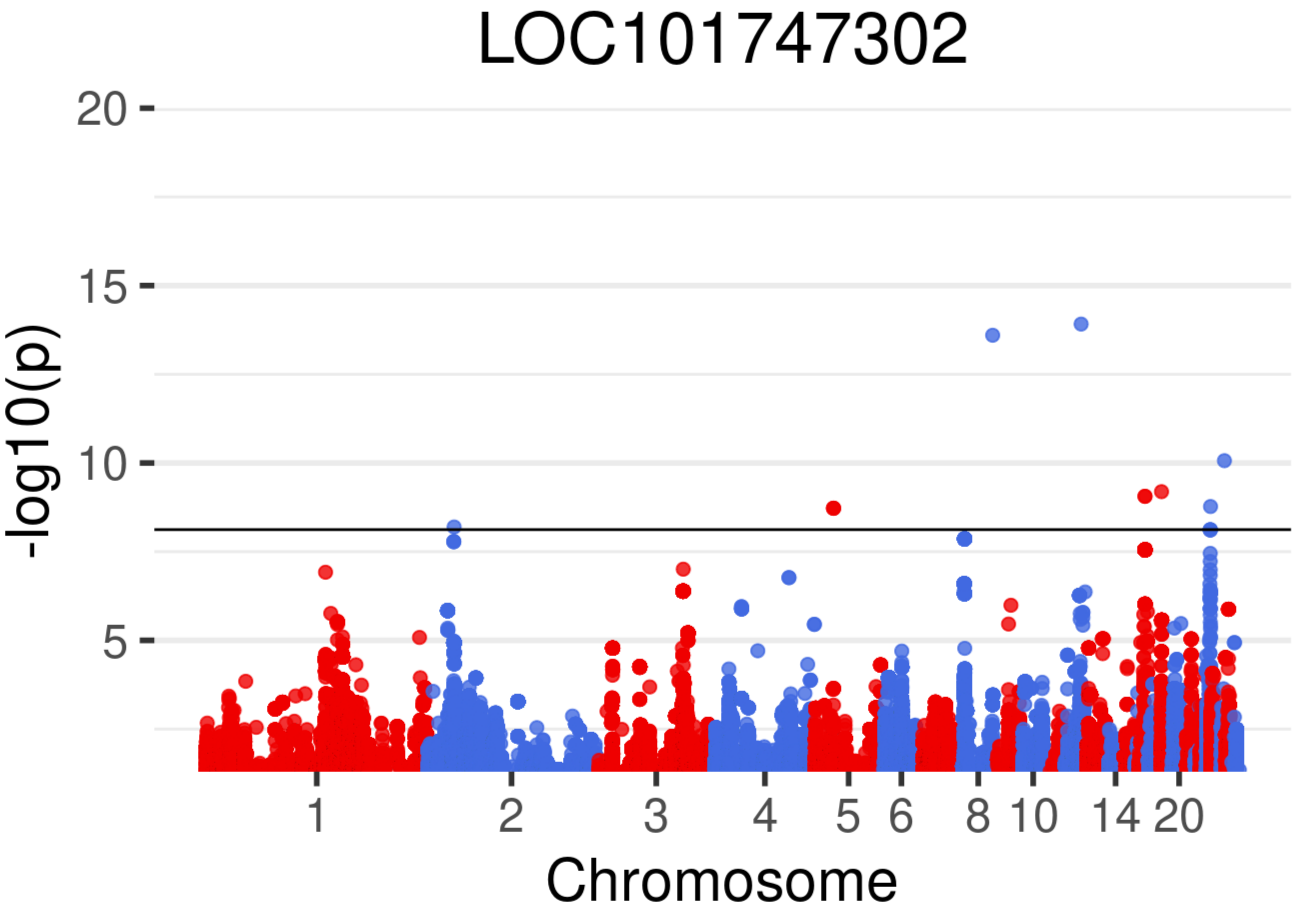
LOC101747302
20
15
10
5
-log10(p)
1
2
3
4
5
6
8
10
14
20
Chromosome

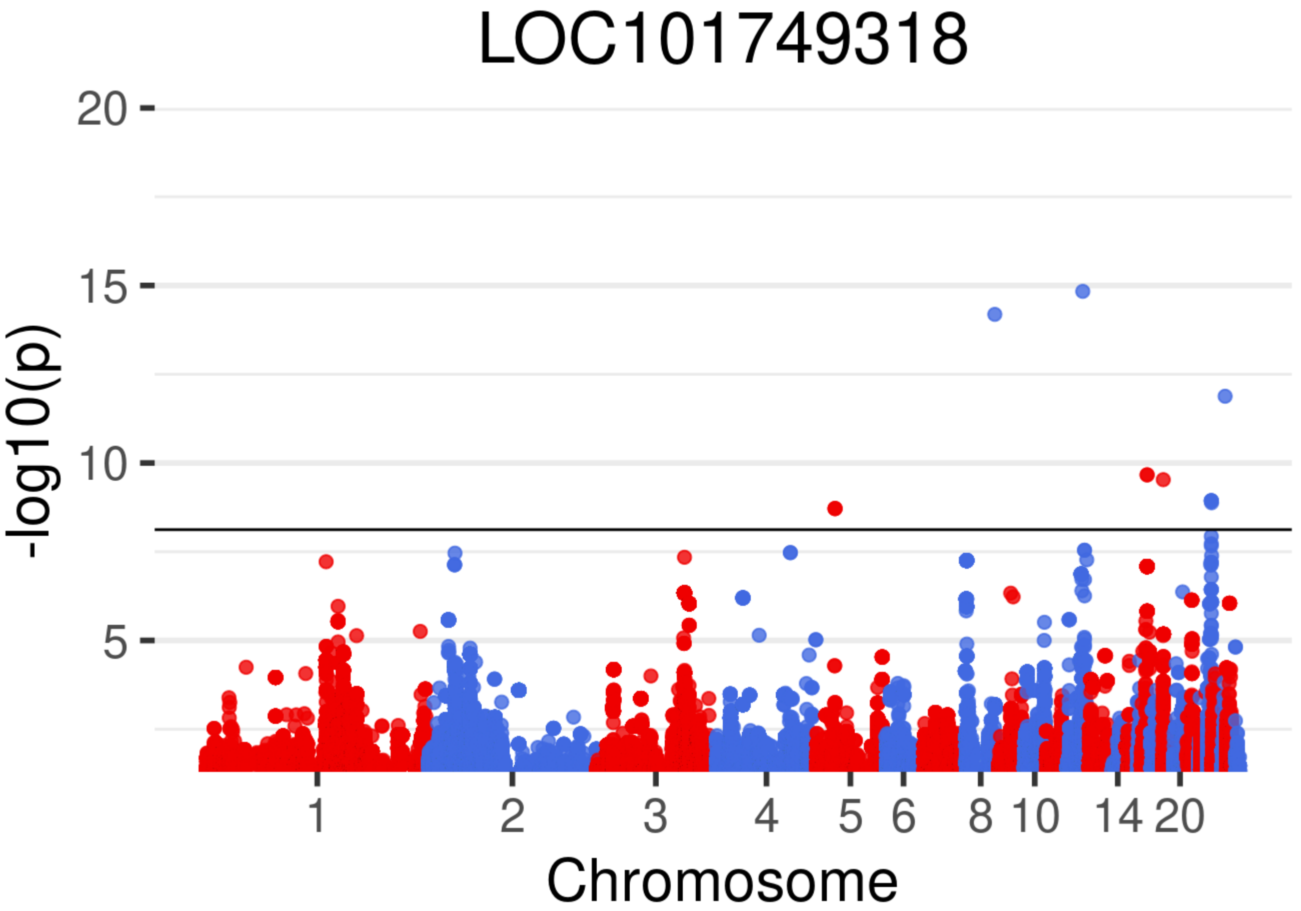
LOC101749318
20
15
10
5
-log10(p)
1
2
3
4
5
6
8
10
14
20
Chromosome

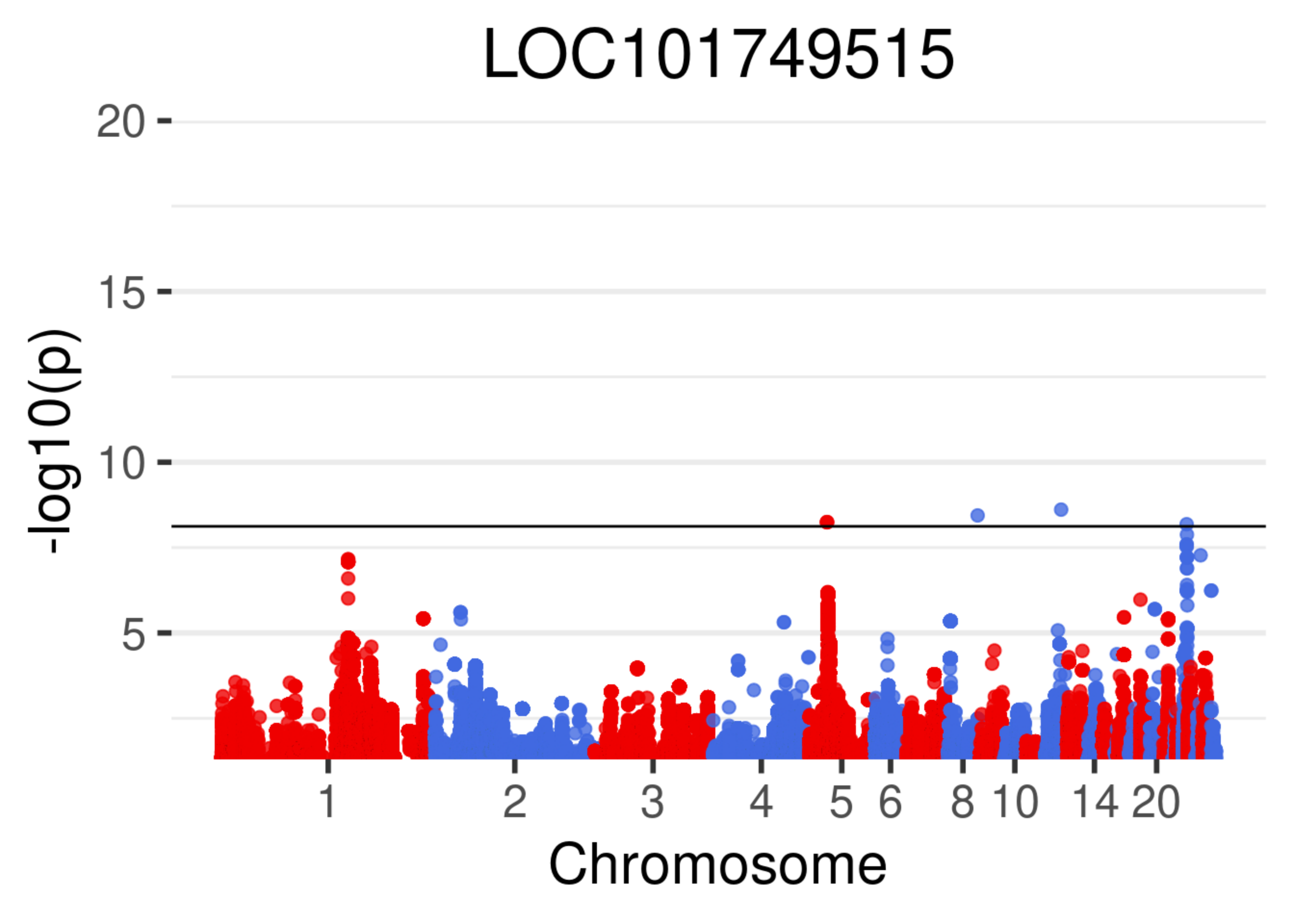

LOC101749515
-log10(p)
20
15
10
5
1
2
3
4
5
6
8
10
14
20
Chromosome

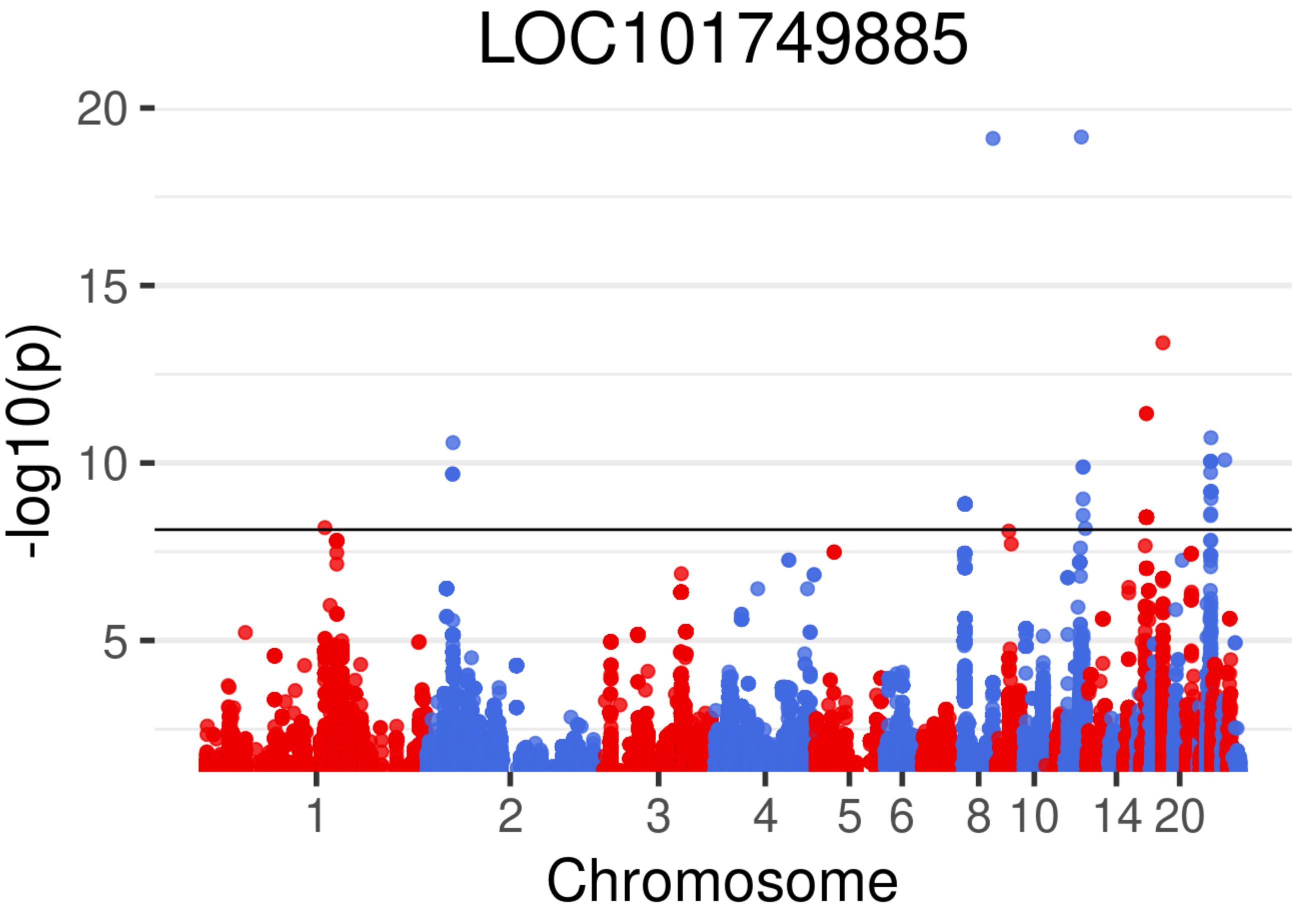

LOC101749885
-log10(p)
20
15
10
5
1
2
3
4
5
6
8
10
14
20
Chromosome

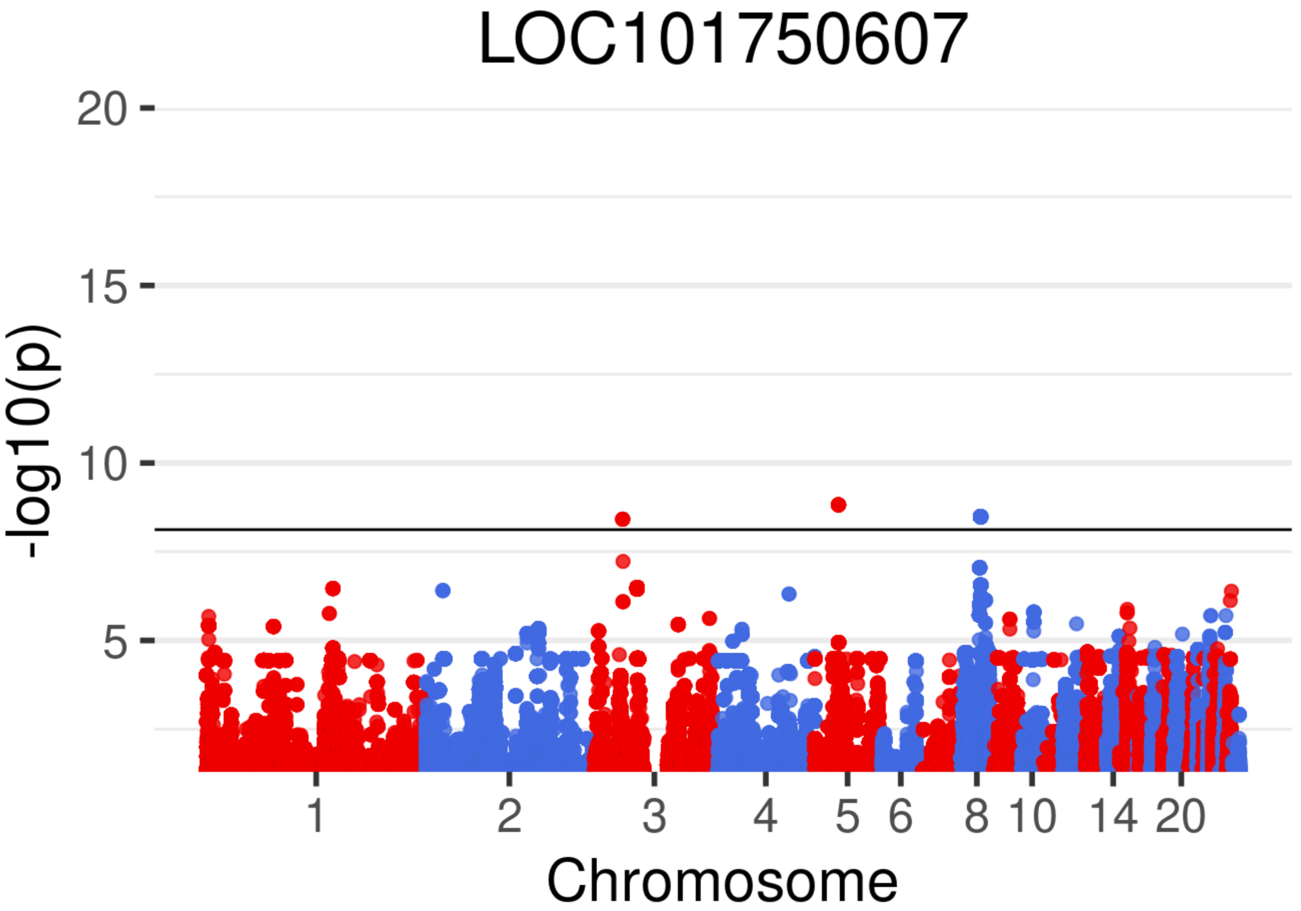

LOC101750607
20
15
10
5
-log10(p)
1
2
3
4
5
6
8
10
14
20
Chromosome

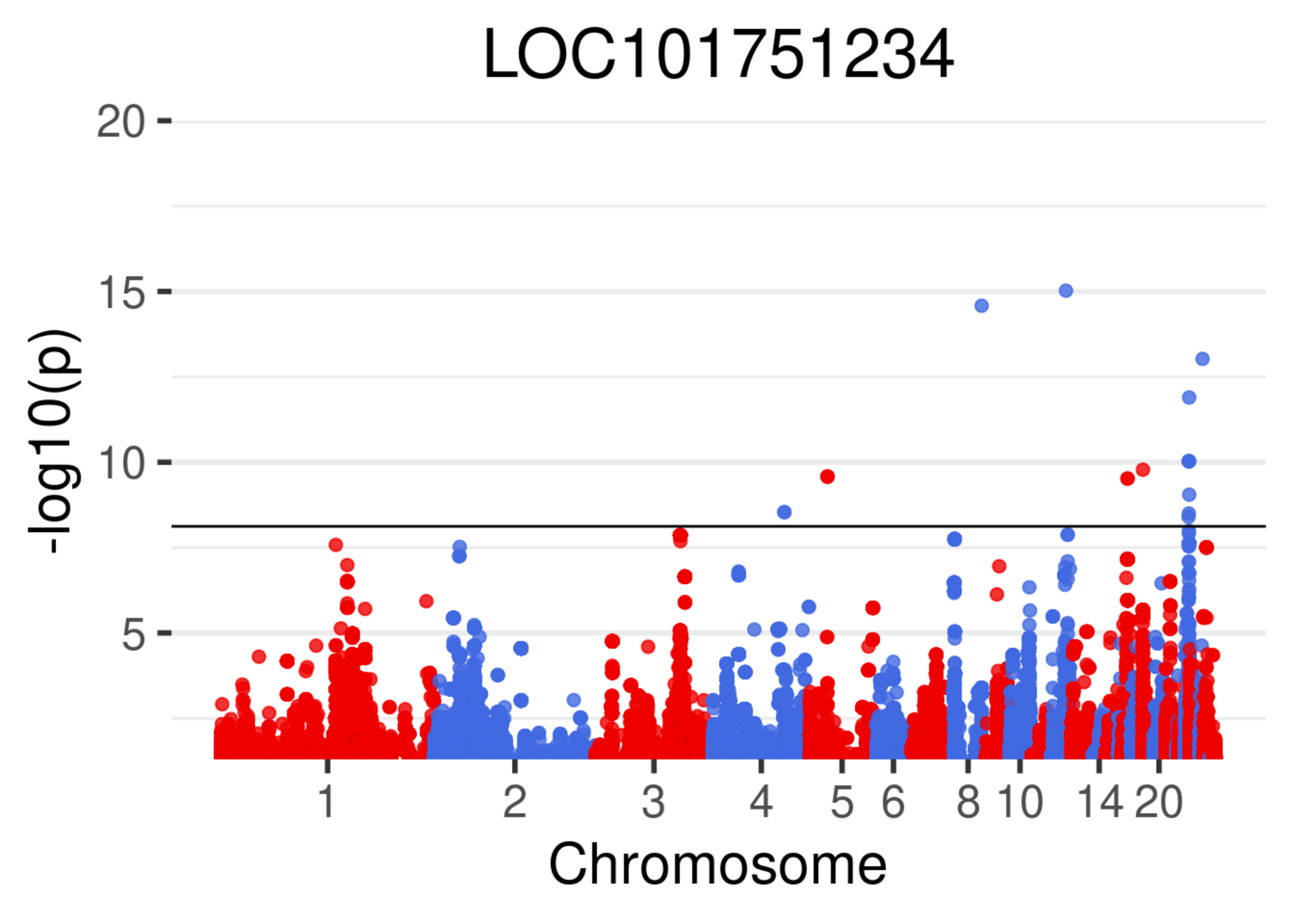
LOC101751234
20
15
10
5
-log10(p)
1
2
3
4
5
6
8
10
14
20
Chromosome

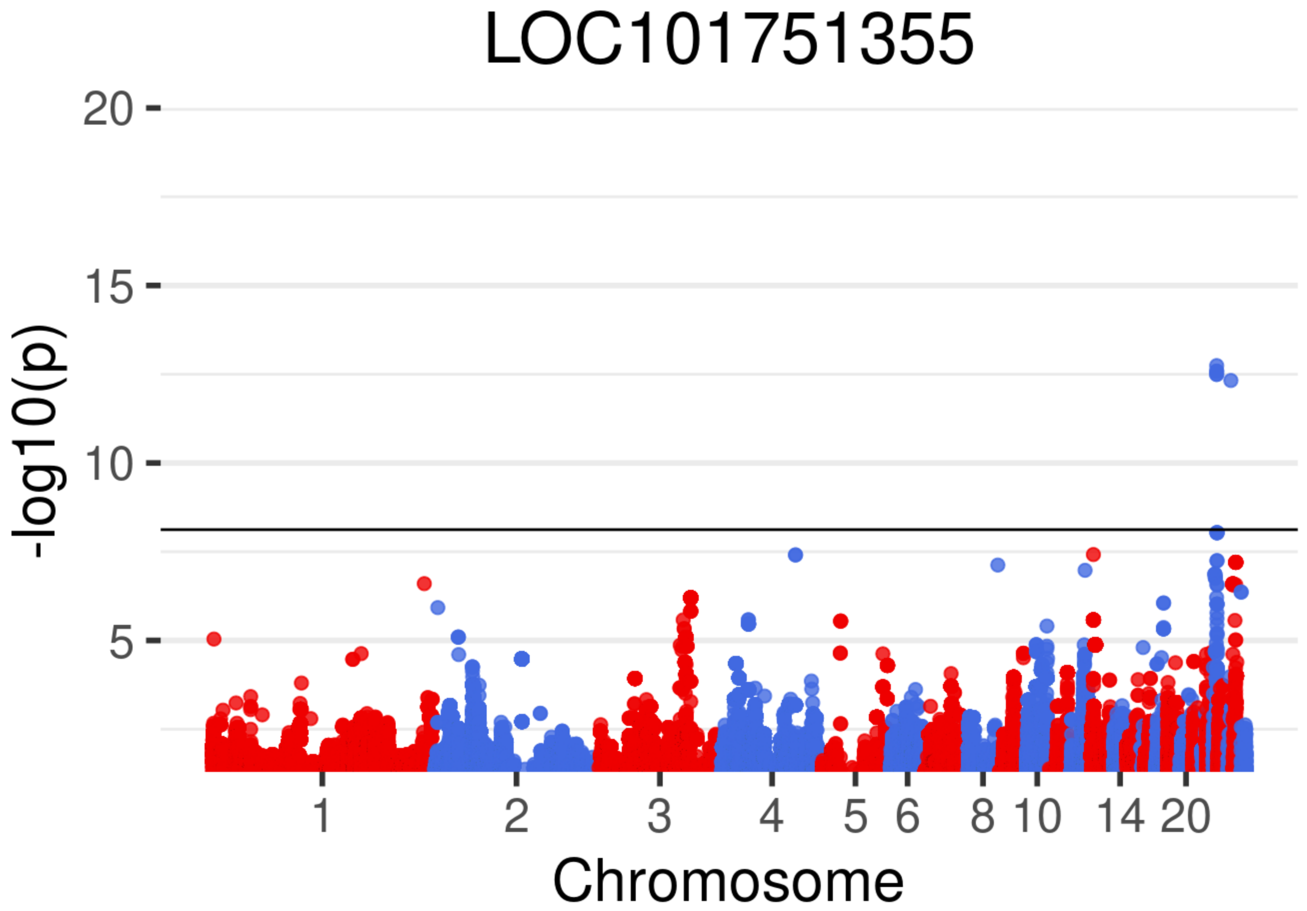
LOC101751355
20
15
10
5
-log10(p)
1
2
3
4
5
6
8
10
14
20
Chromosome

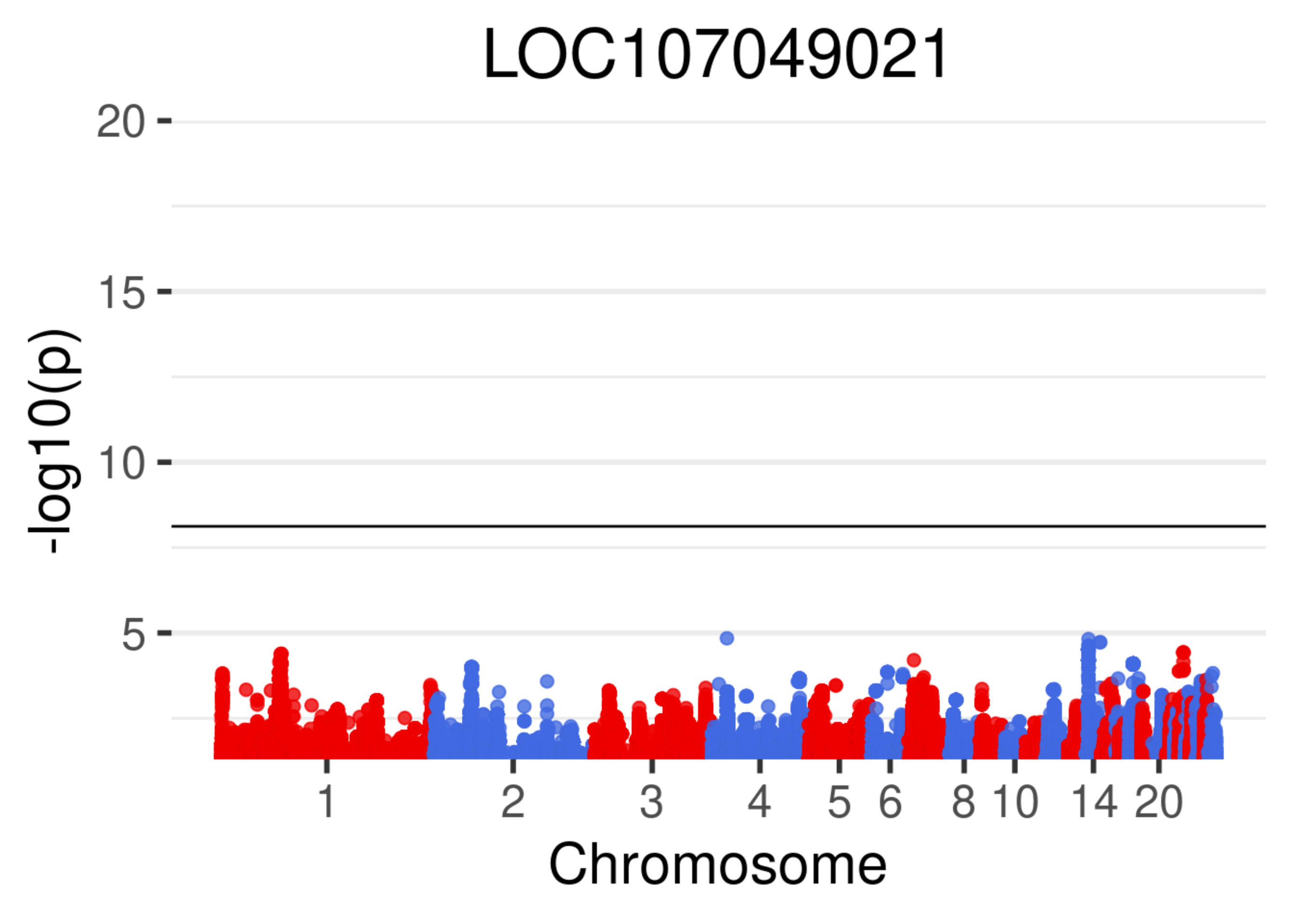
LOC107049021
20
15
10
5
-log10(p)
1
2
3
4
5
6
8
10
14
20
Chromosome

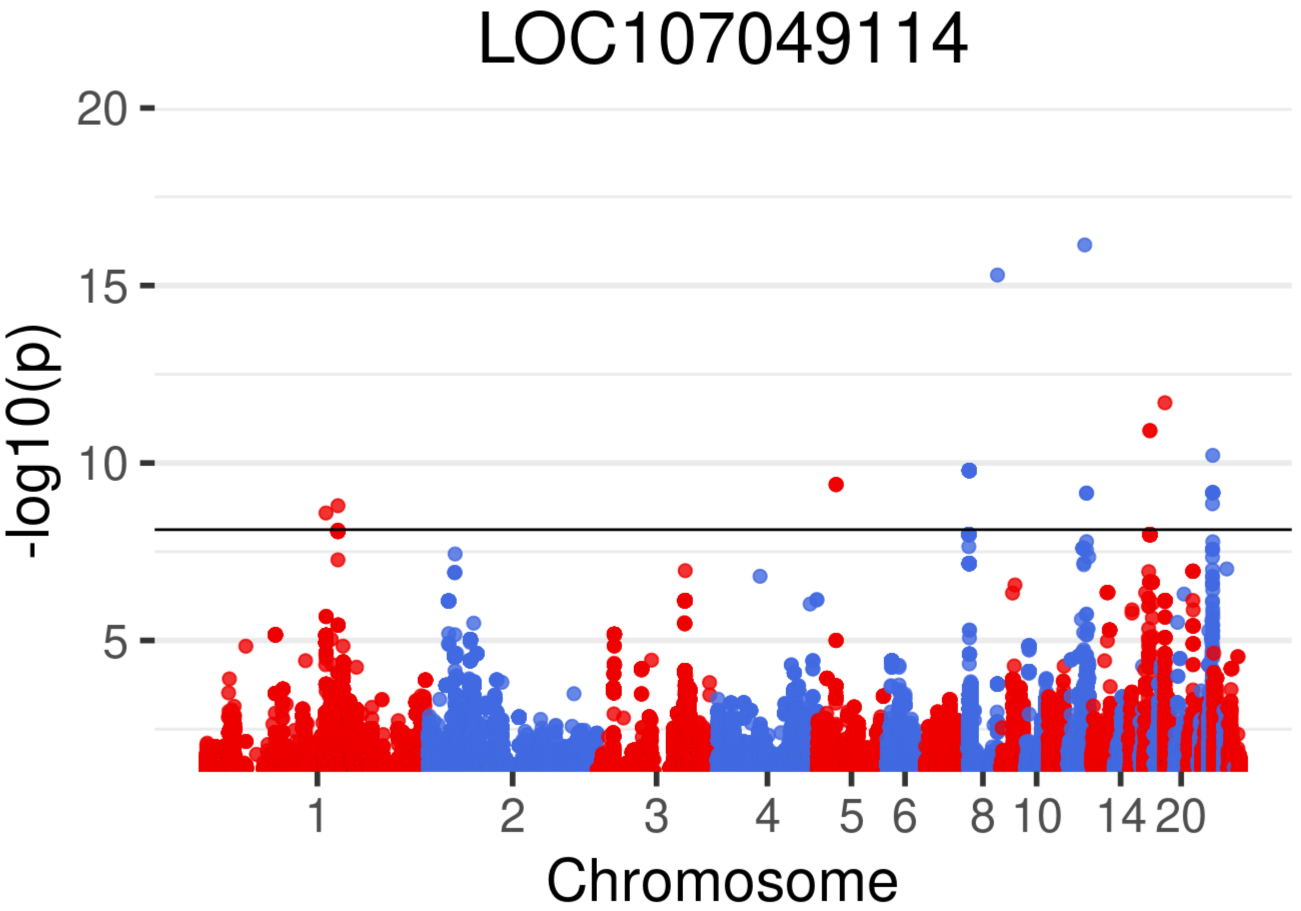
LOC107049114
20
15
10
5
-log10(p)
1
2
3
4
5
6
8
10
14
20
Chromosome

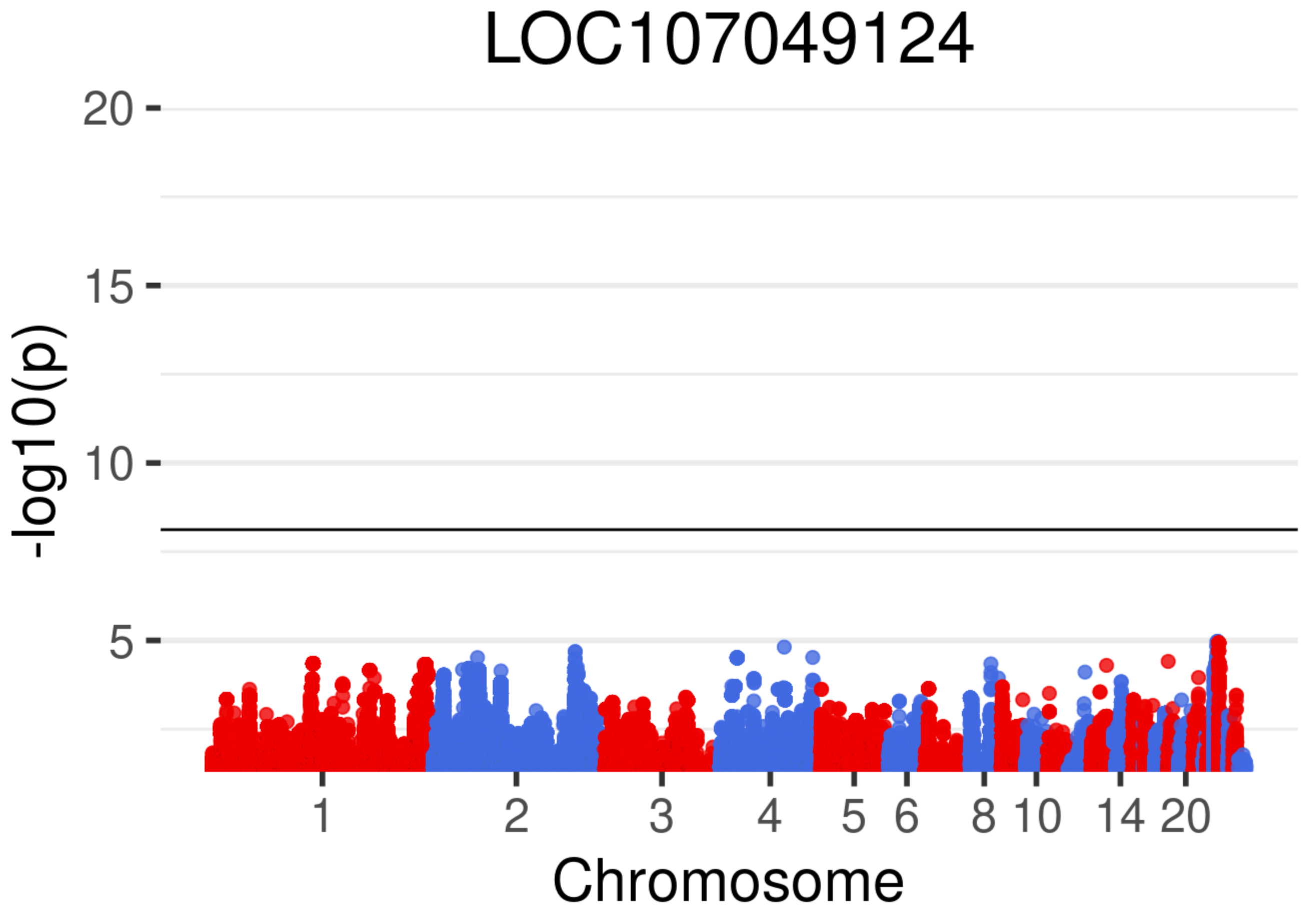
LOC107049124
20
15
10
5
-log10(p)
1
2
3
4
5
6
8
10
14
20
Chromosome

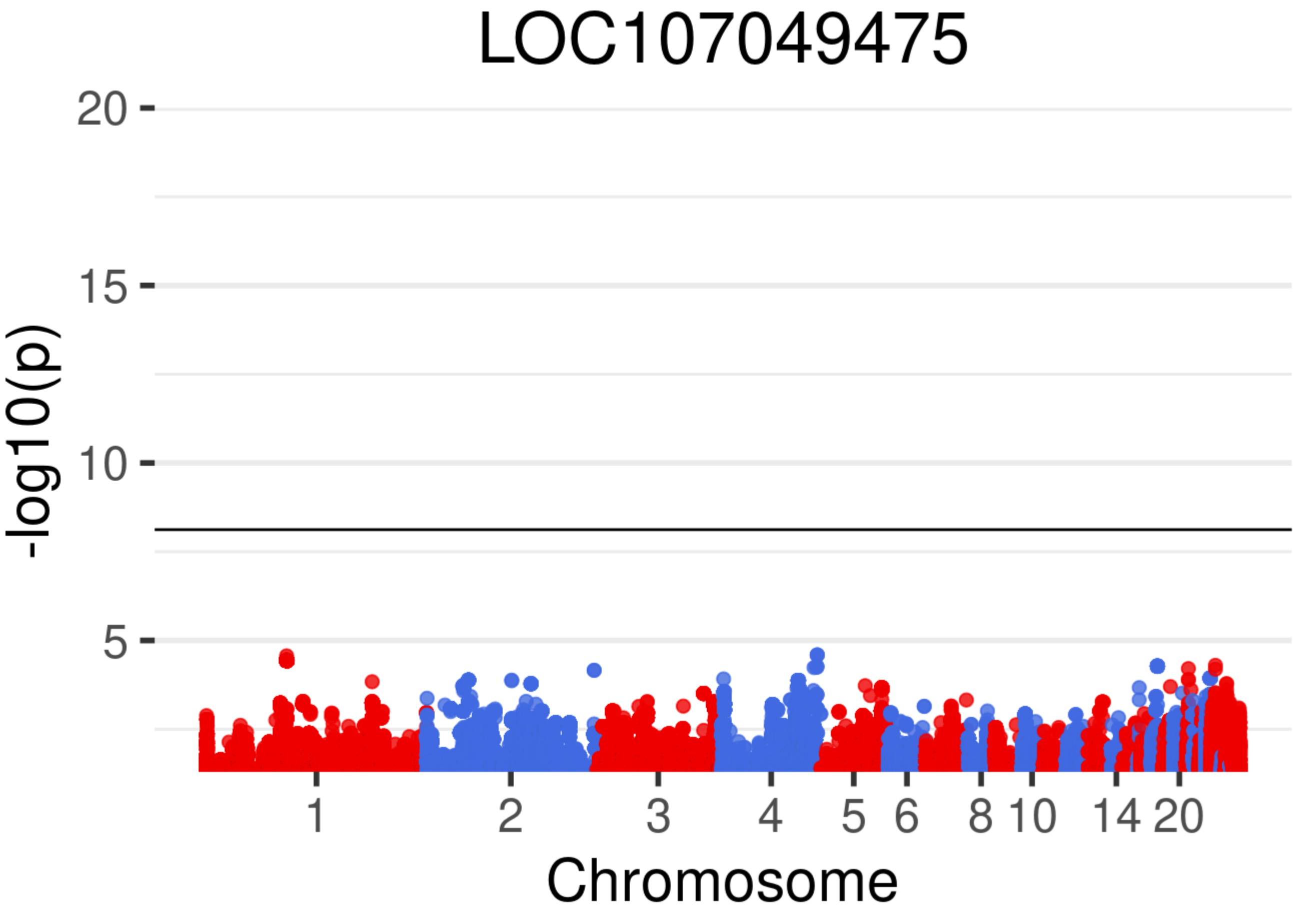
LOC107049475
20
15
10
5
-log10(p)
1
2
3
4
5
6
8
10
14
20
Chromosome

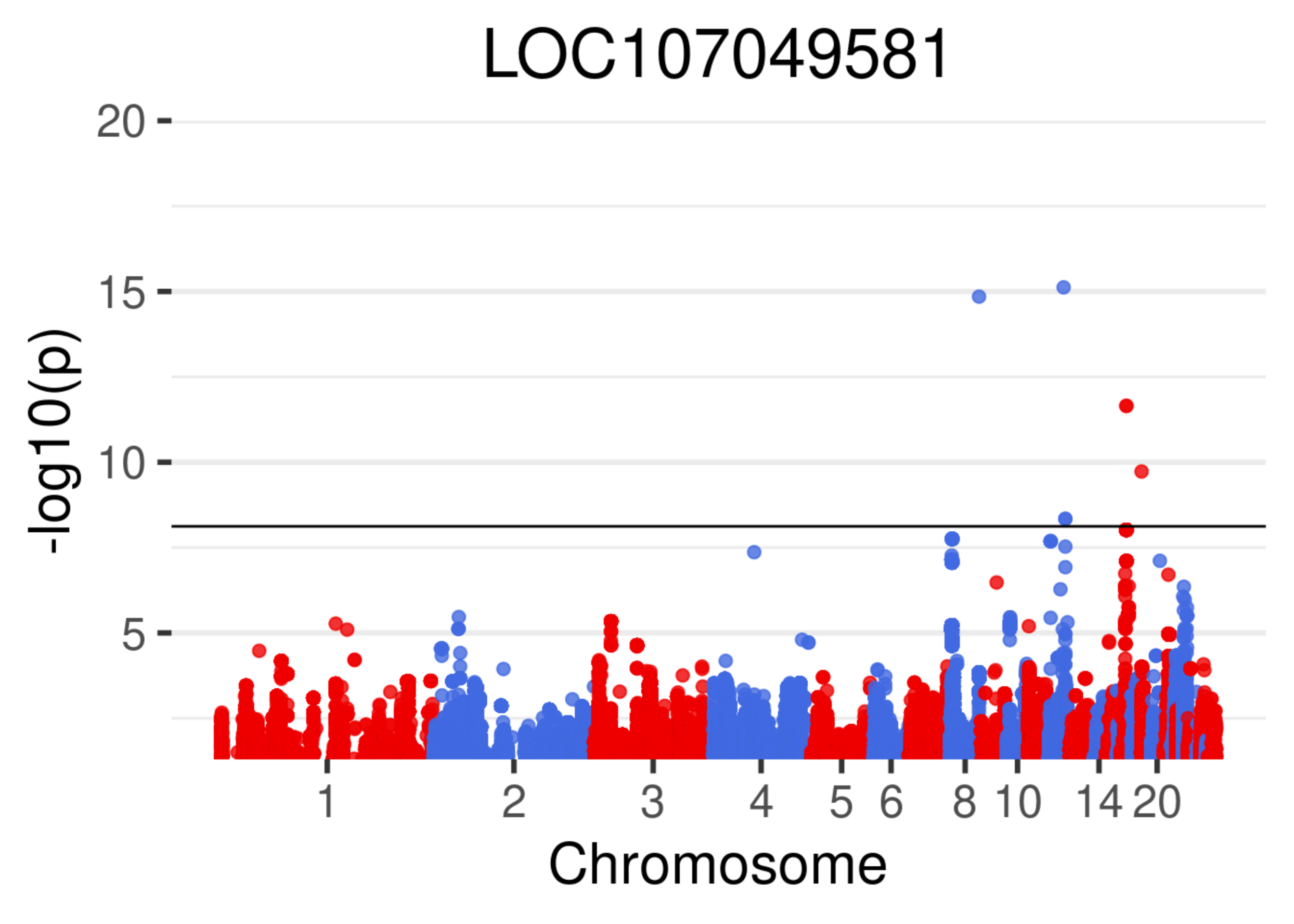

LOC107049581
-log10(p)
20
15
10
5
1
2
3
4
5
6
8
10
14
20
Chromosome

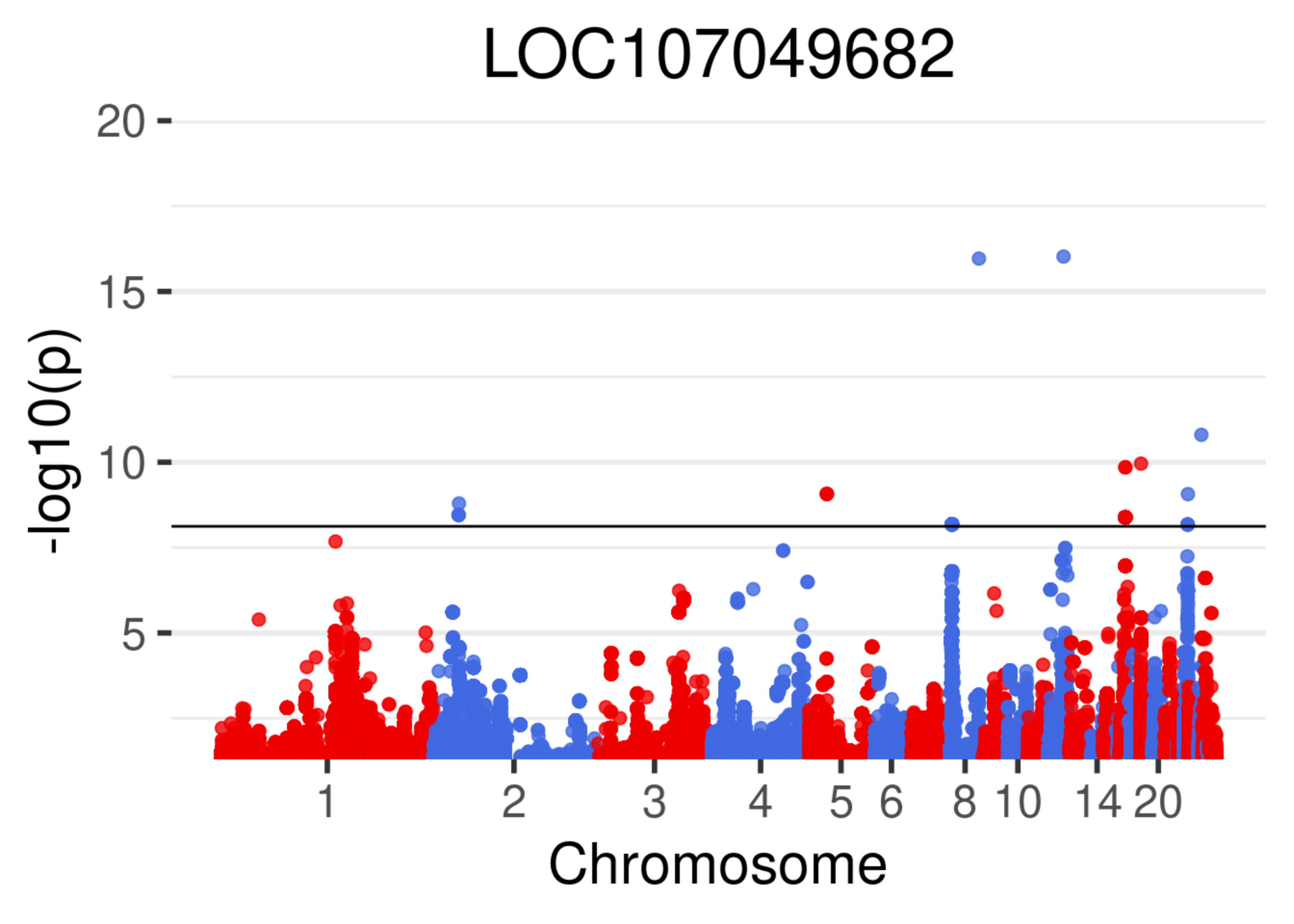

LOC107049682
20
15
10
5
-log10(p)
1
2
3
4
5
6
8
10
14
20
Chromosome

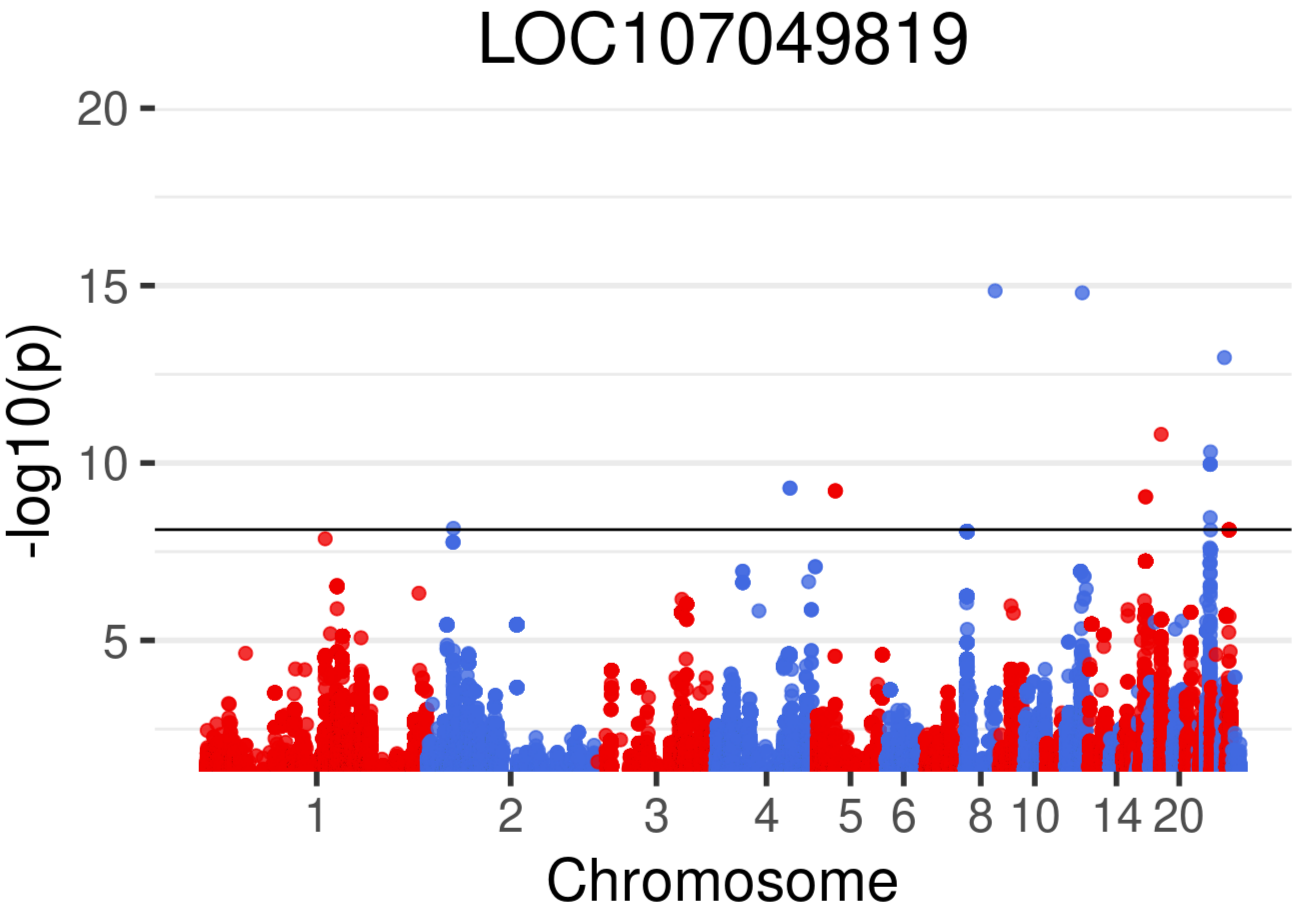
LOC107049819
-log10(p)
20
15
10
5
1
2
3
4
5
6
8
10
14
20
Chromosome

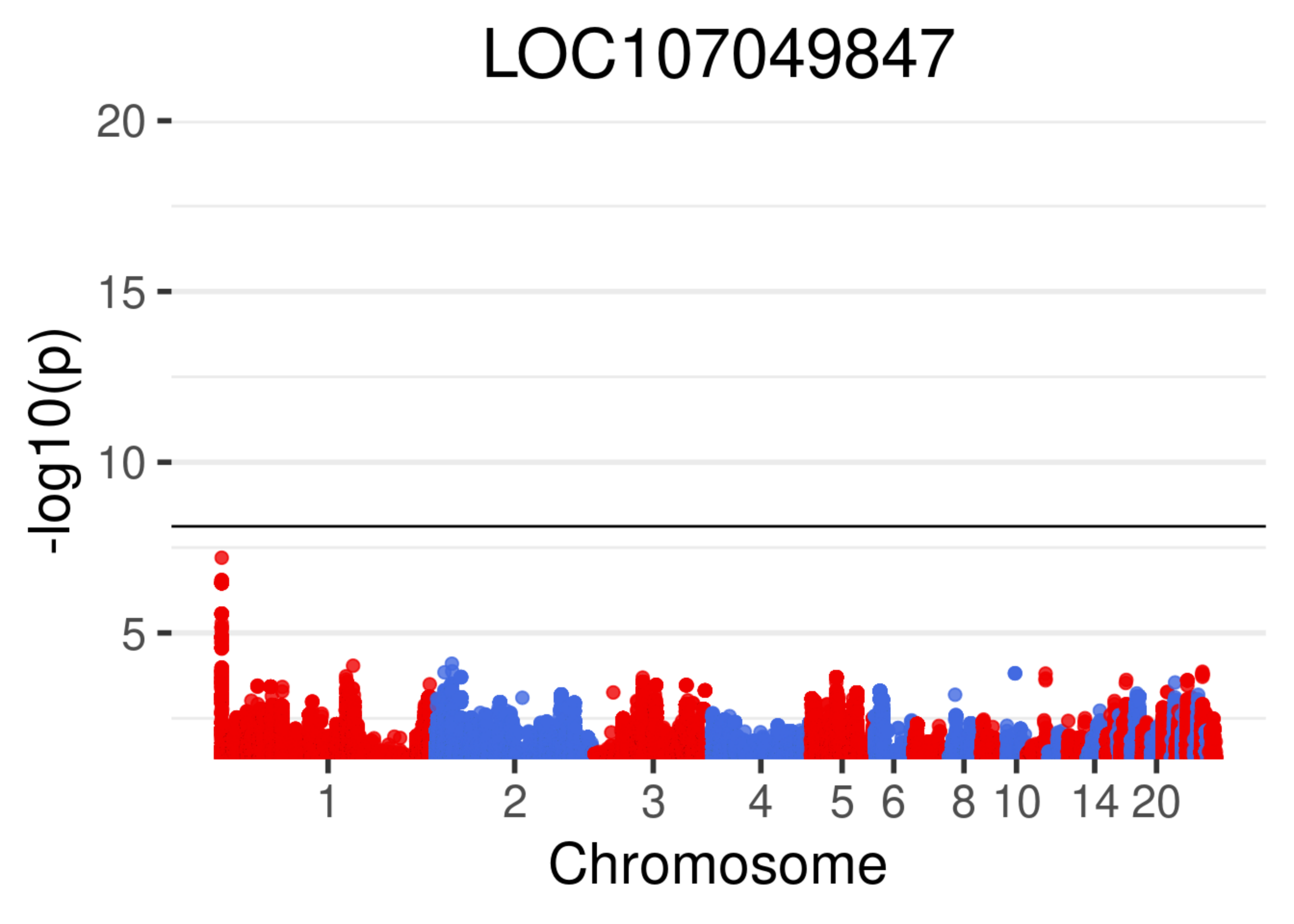

LOC107049847
-log10(p)
20
15
10
5
1
2
3
4
5
6
8
10
14
20
Chromosome

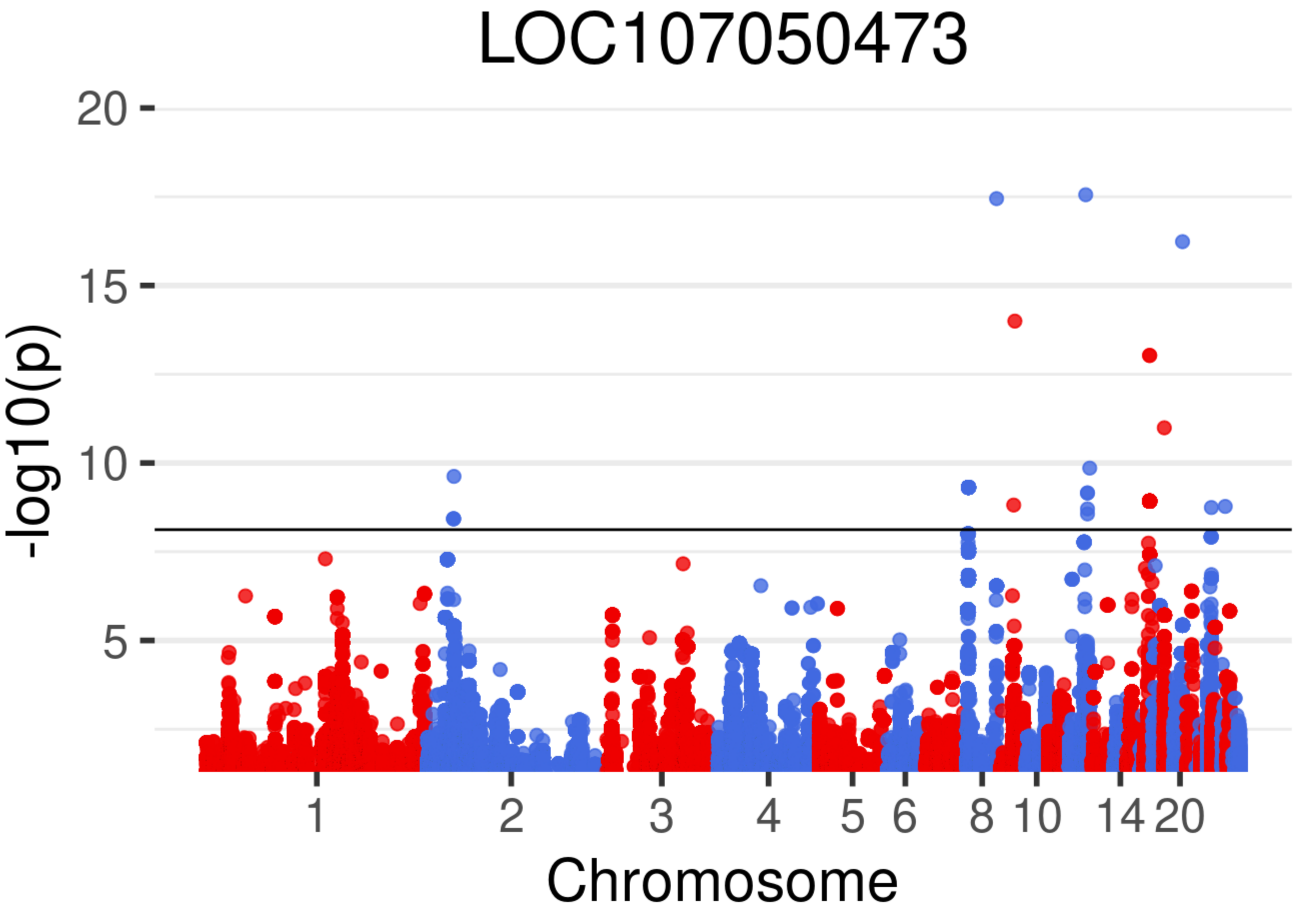
LOC107050473
20
15
10
5
-log10(p)
1
2
3
4
5
6
8
10
14
20
Chromosome

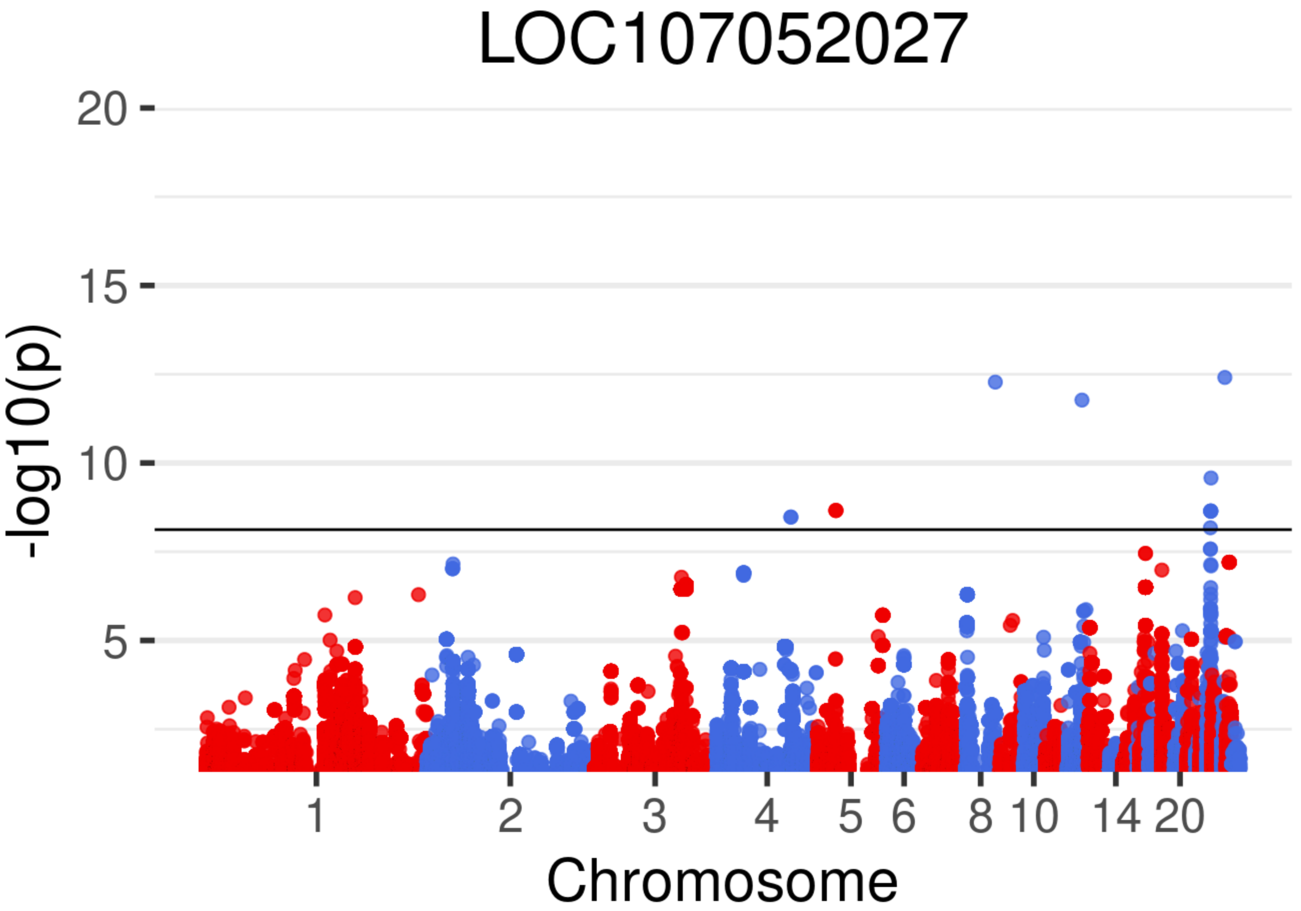
LOC107052027
-log10(p)
20
15
10
5
1
2
3
4
5
6
8
10
14
20
Chromosome

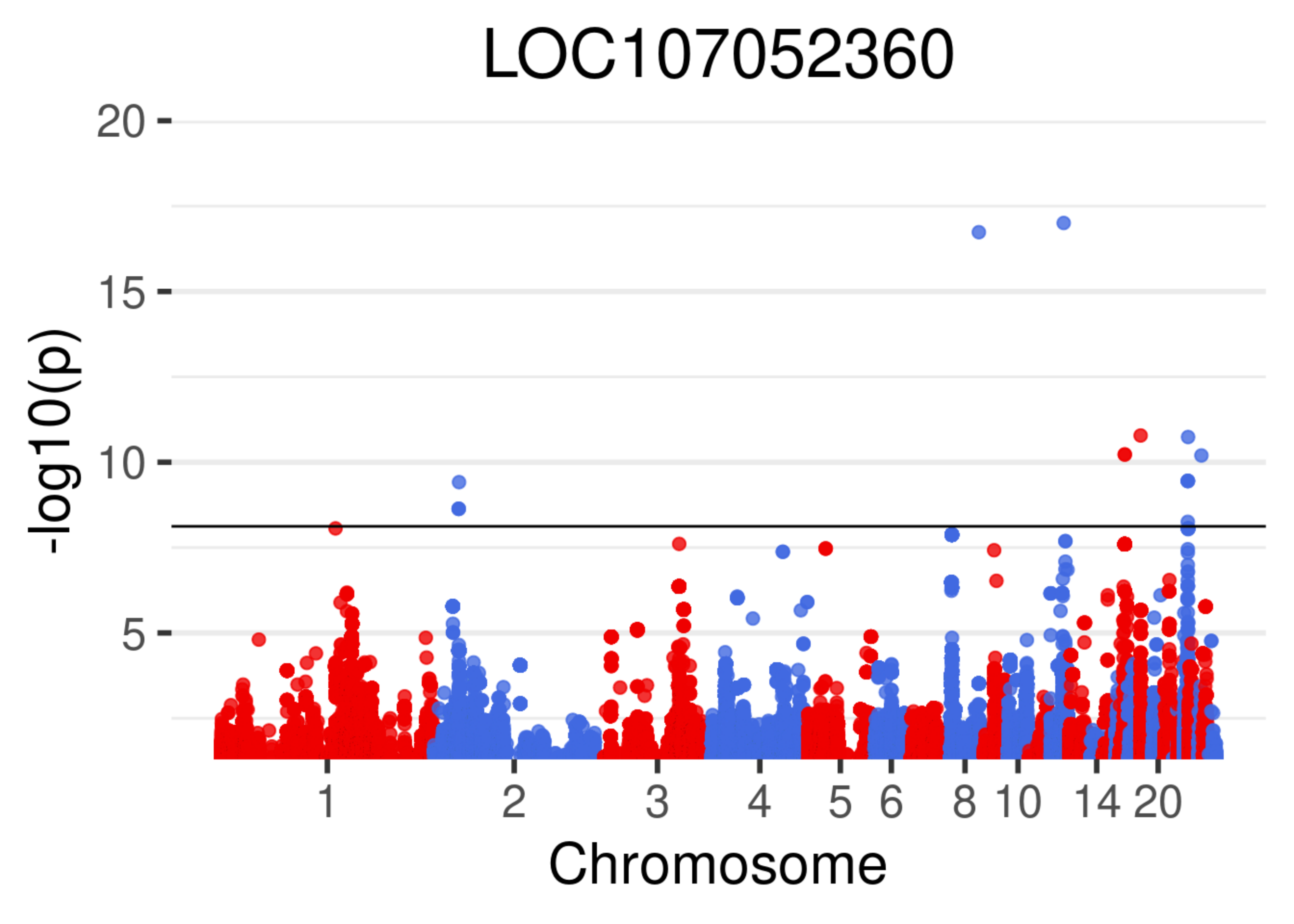
LOC107052360
20
15
10
5
-log10(p)
1
2
3
4
5
6
8
10
14
20
Chromosome

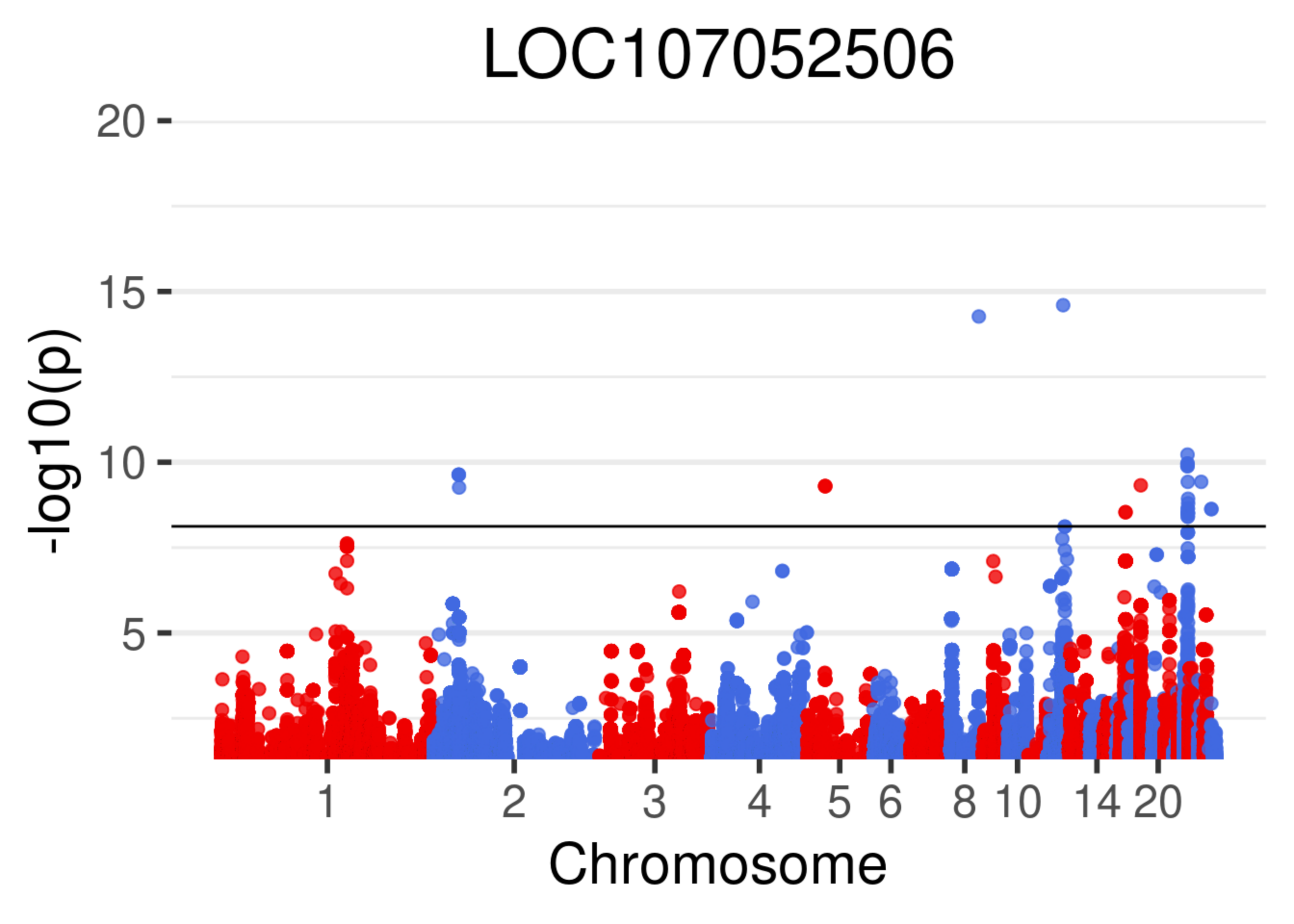

LOC107052506
20
15
10
5
-log10(p)
1
2
3
4
5
6
8
10
14
20
Chromosome

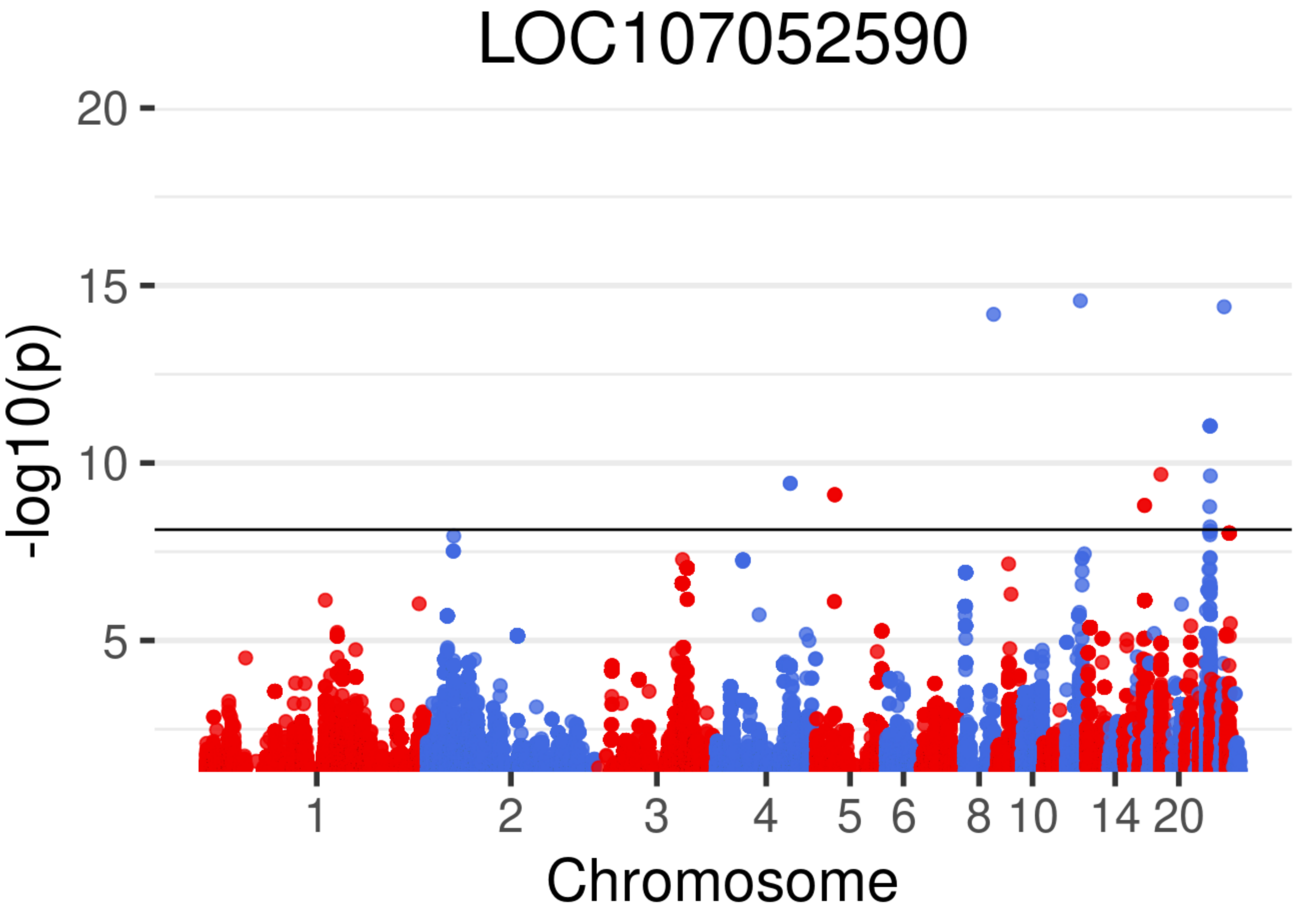
LOC107052590
-log10(p)
20
15
10
5
1
2
3
4
5
6
8
10
14
20
Chromosome

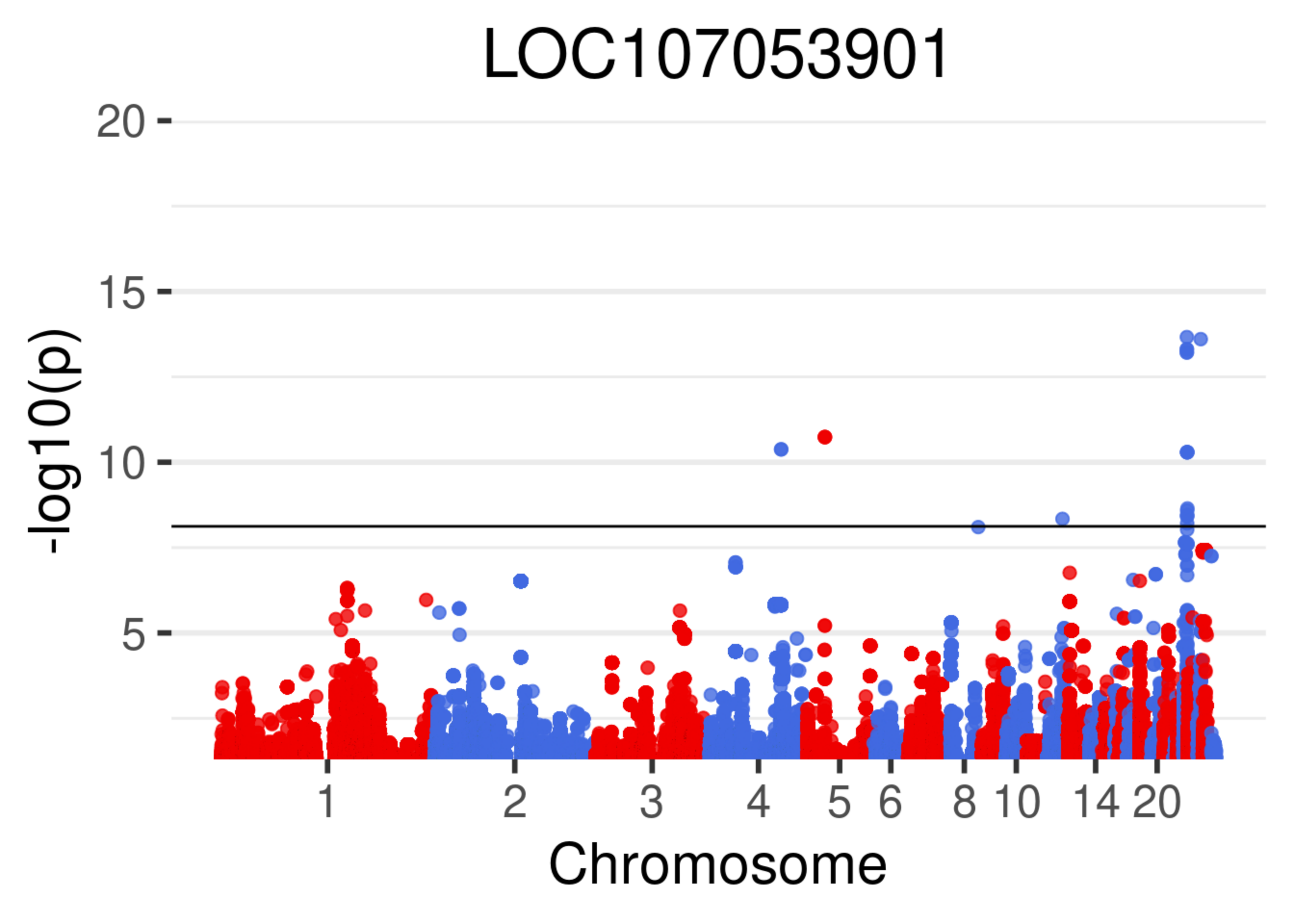

LOC107053901
-log10(p)
20
15
10
5
1
2
3
4
5
6
8
10
14
20
Chromosome

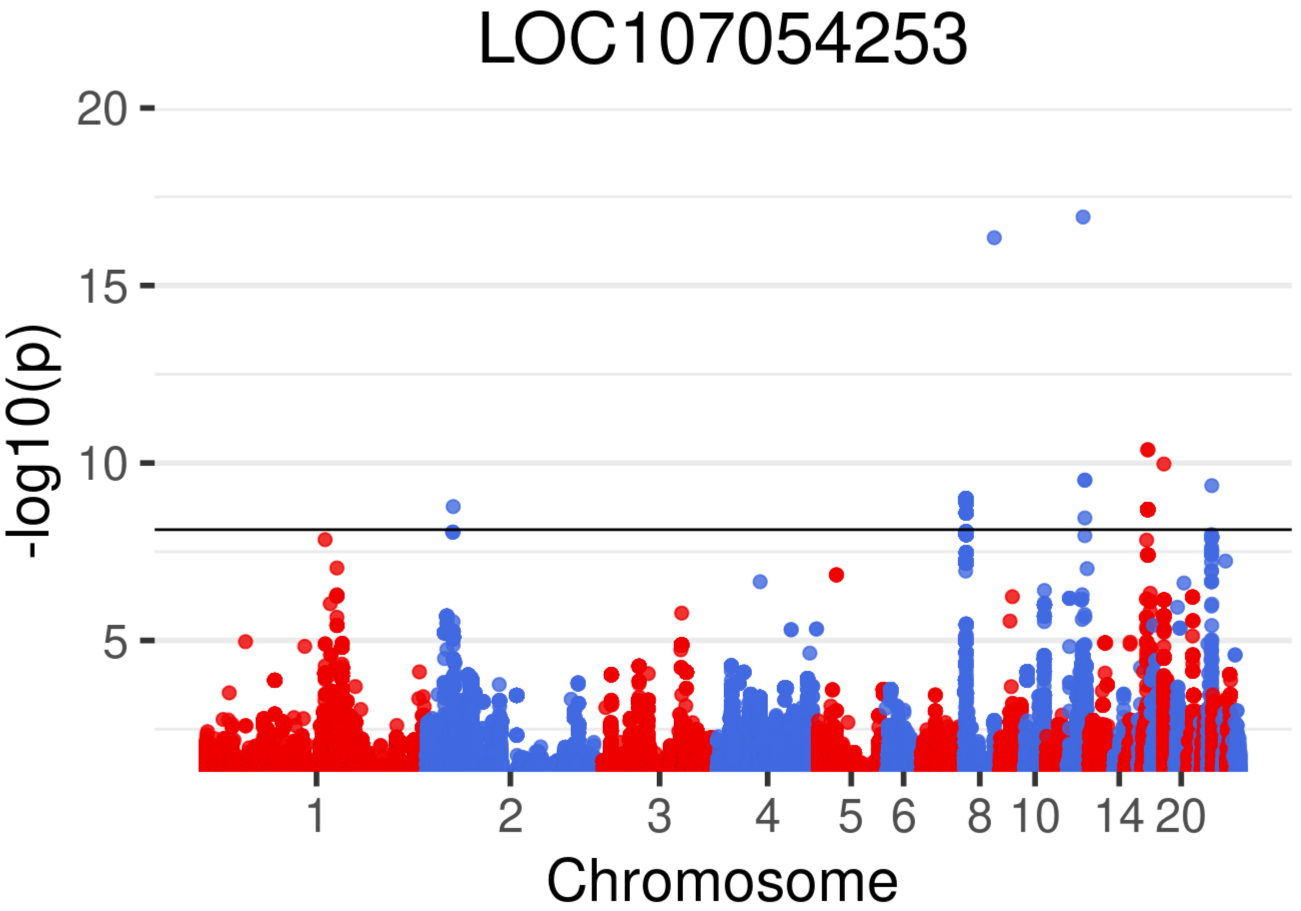
LOC107054253
-log10(p)
20
15
10
5
1
2
3
4
5
6
8
10
14
20
Chromosome

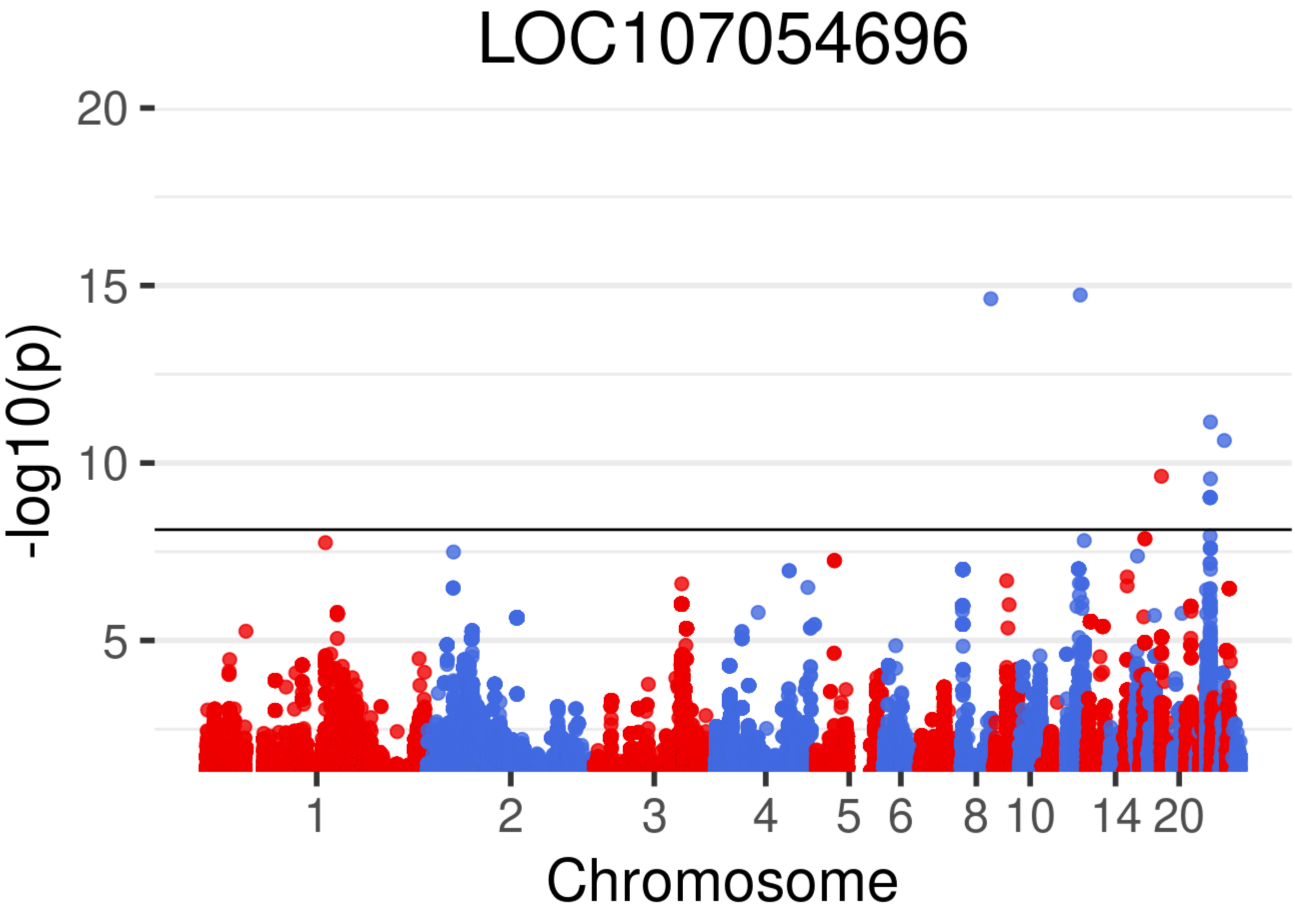
LOC107054696
20
15
10
5
-log10(p)
1
2
3
4
5
6
8
10
14
20
Chromosome

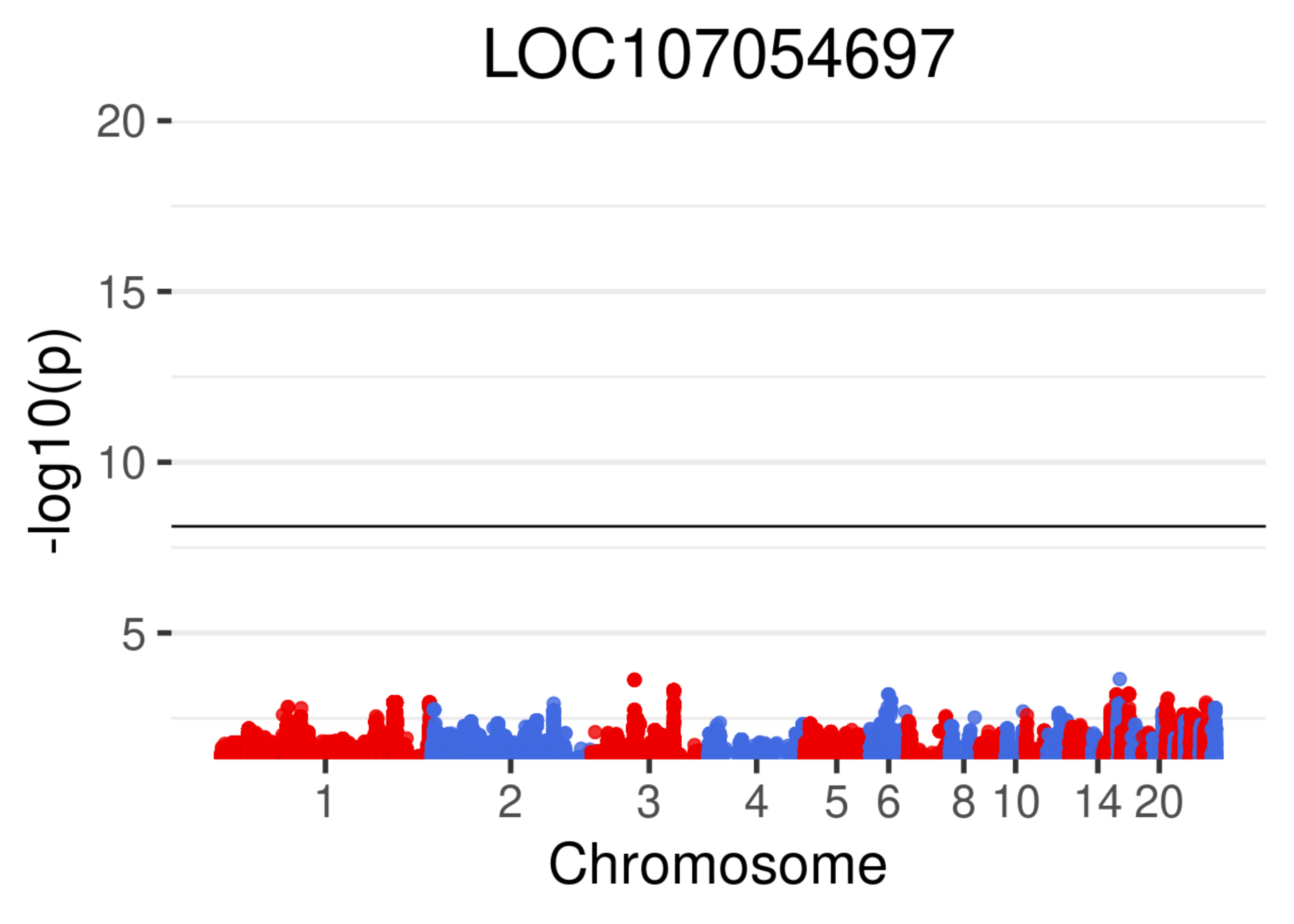
LOC107054697
20
15
10
5
-log10(p)
1
2
3
4
5
6
8
10
14
20
Chromosome

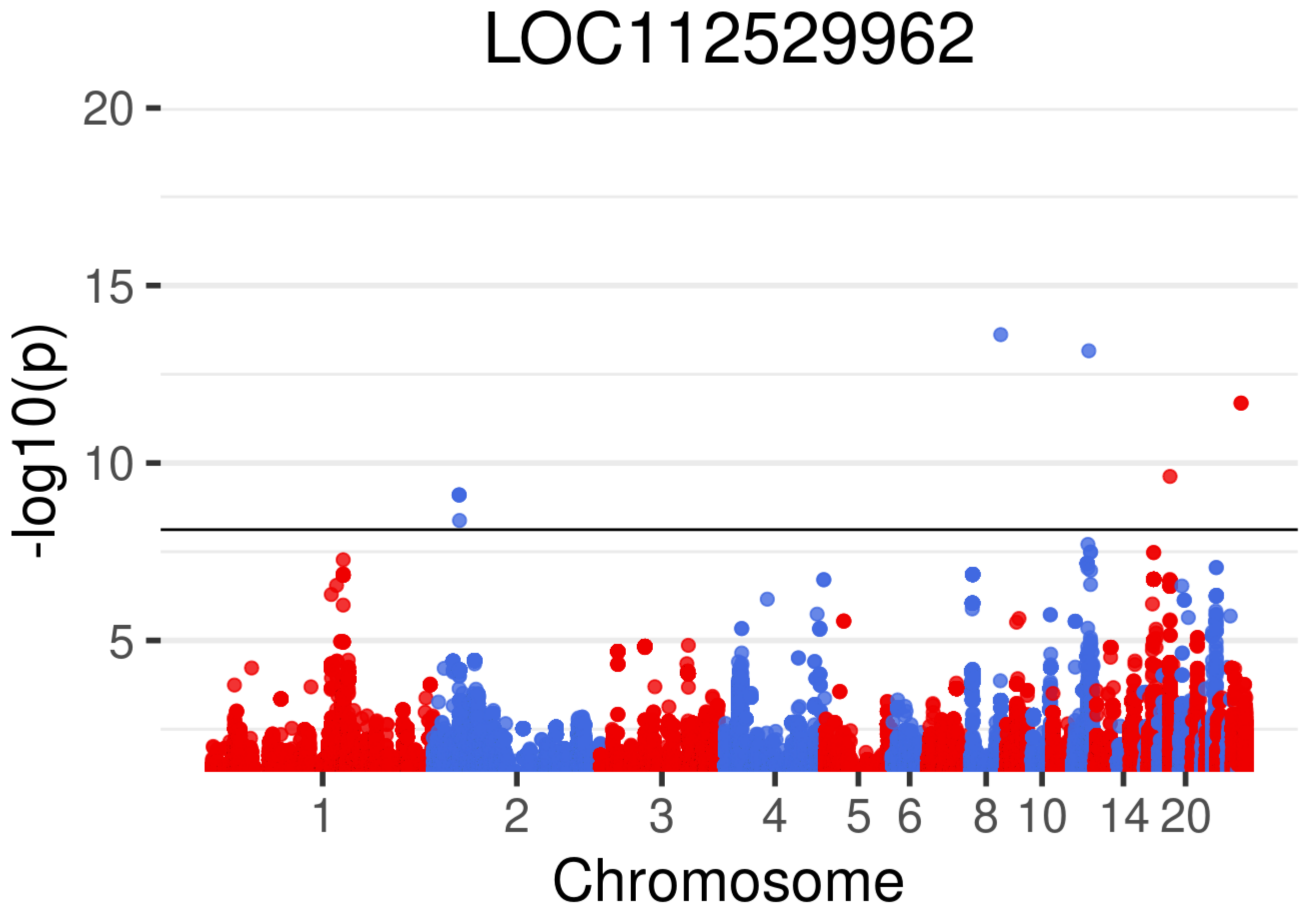

LOC112529962
-log10(p)
20
15
10
5
1
2
3
4
5
6
8
10
14
20
Chromosome

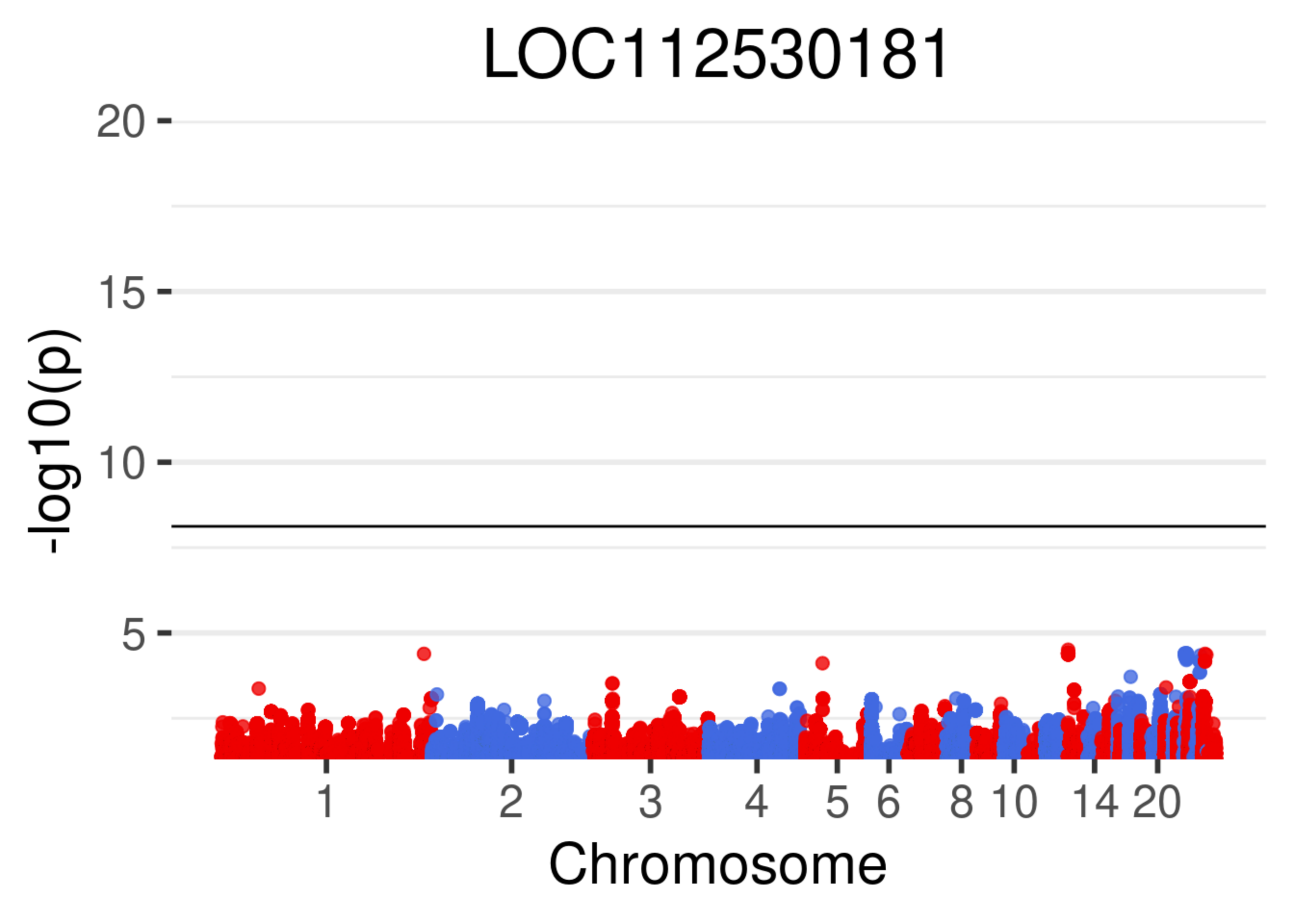
LOC112530181
20
15
10
5
-log10(p)
1
2
3
4
5
6
8
10
14
20
Chromosome

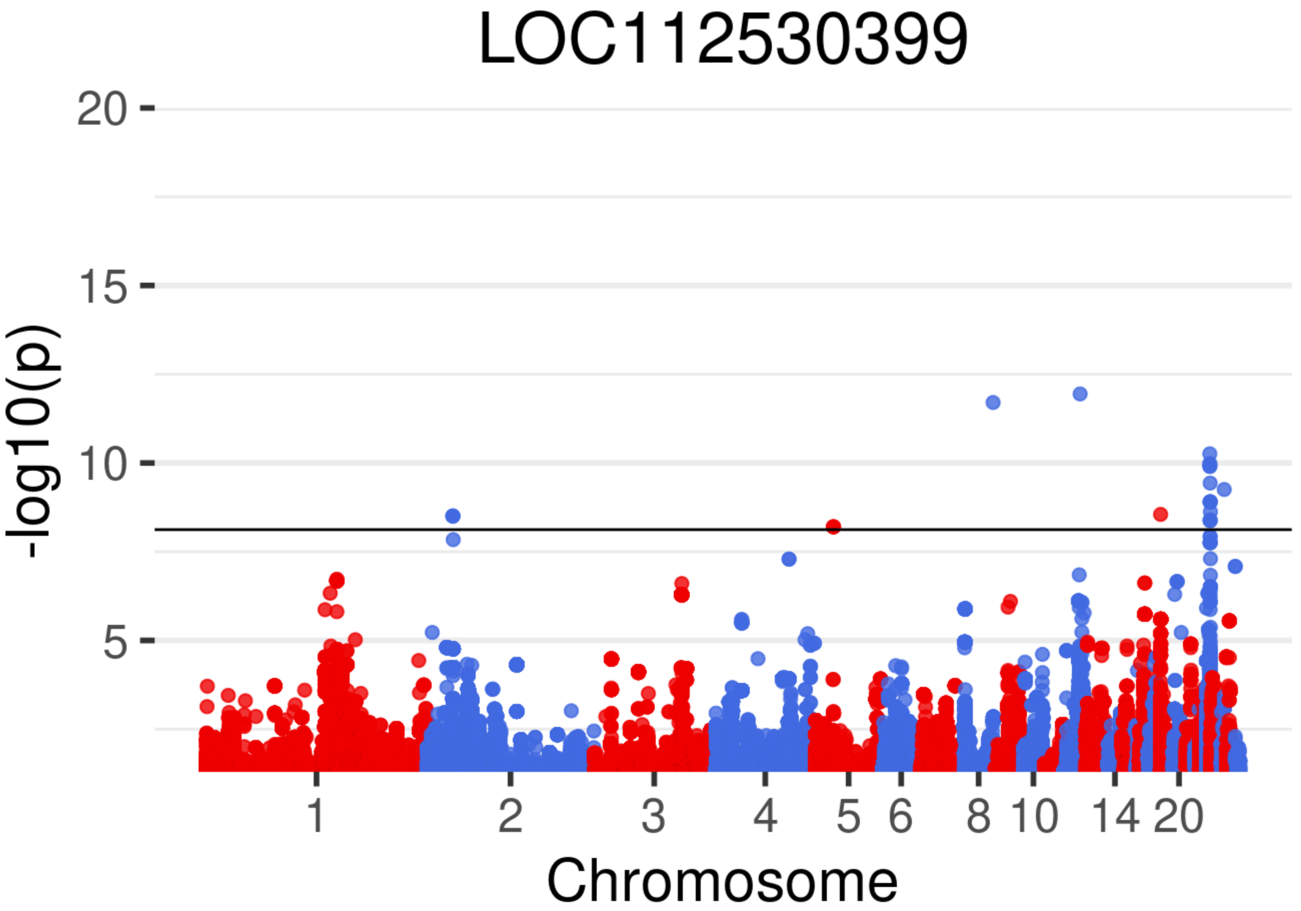

LOC112530399
-log10(p)
20
15
10
5
1
2
3
4
5
6
8
10
14
20
Chromosome

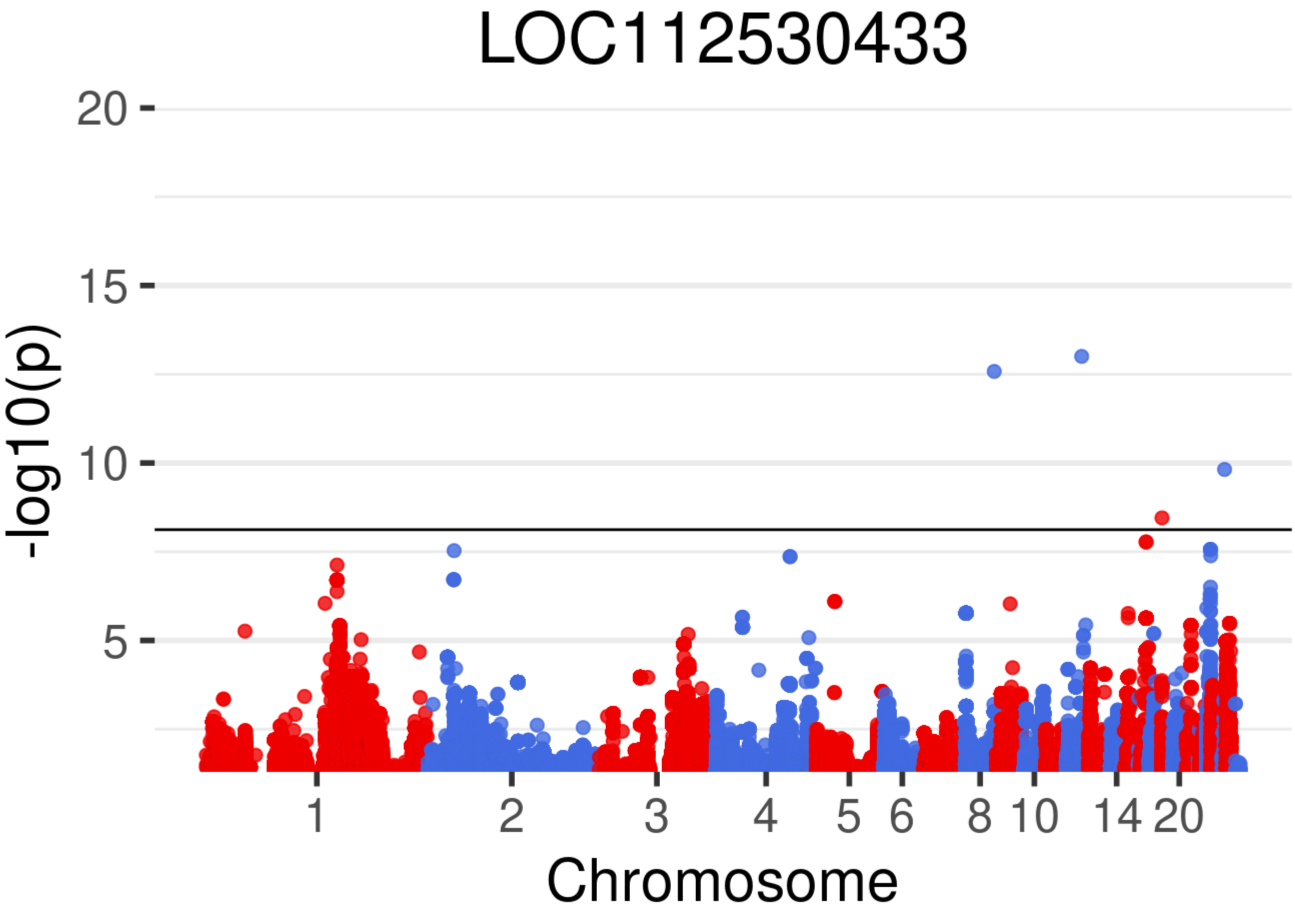
LOC112530433
20
15
10
5
-log10(p)
1
2
3
4
5
6
8
10
14
20
Chromosome

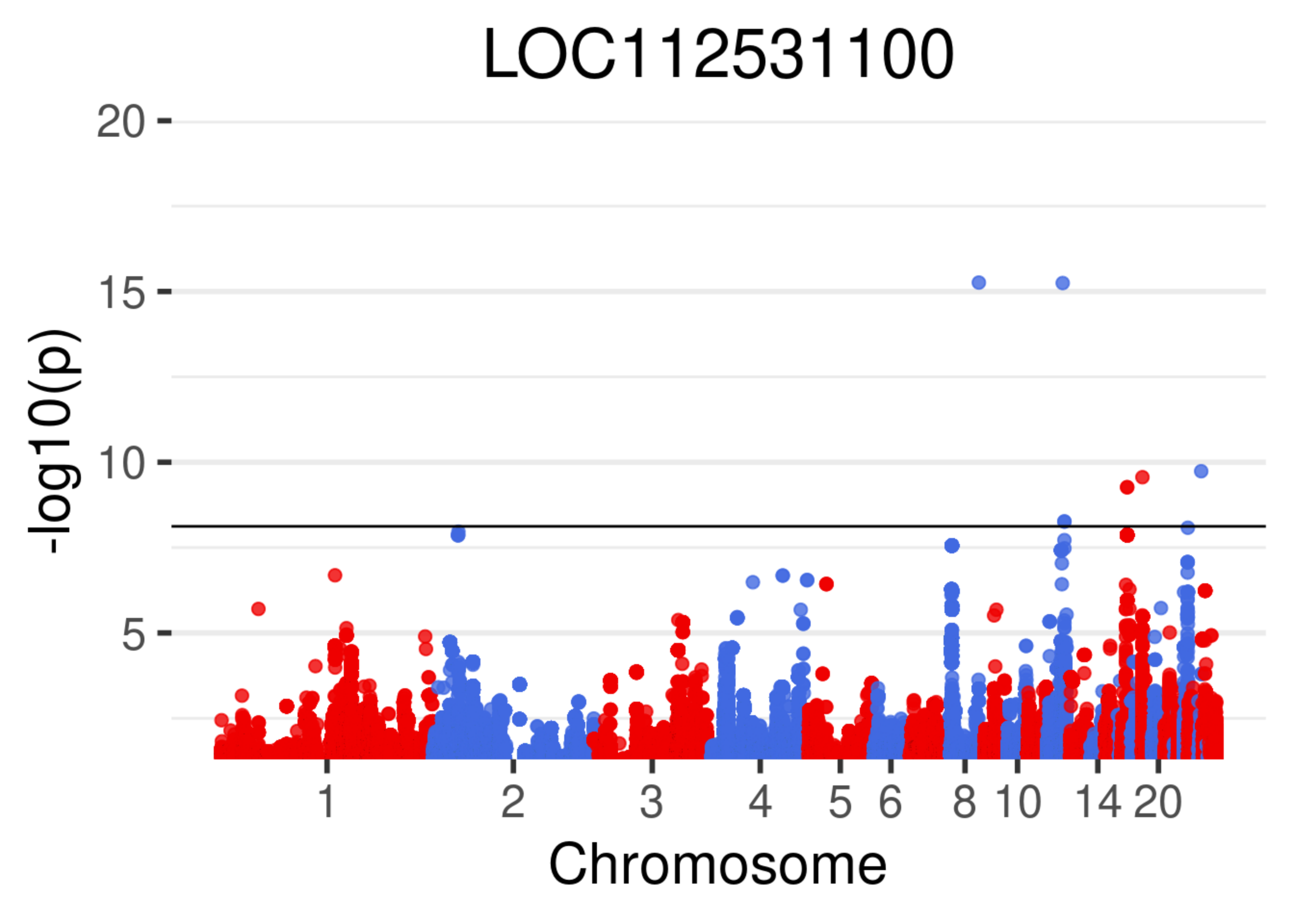
LOC112531100
20
15
10
5
-log10(p)
1
2
3
4
5
6
8
10
14
20
Chromosome

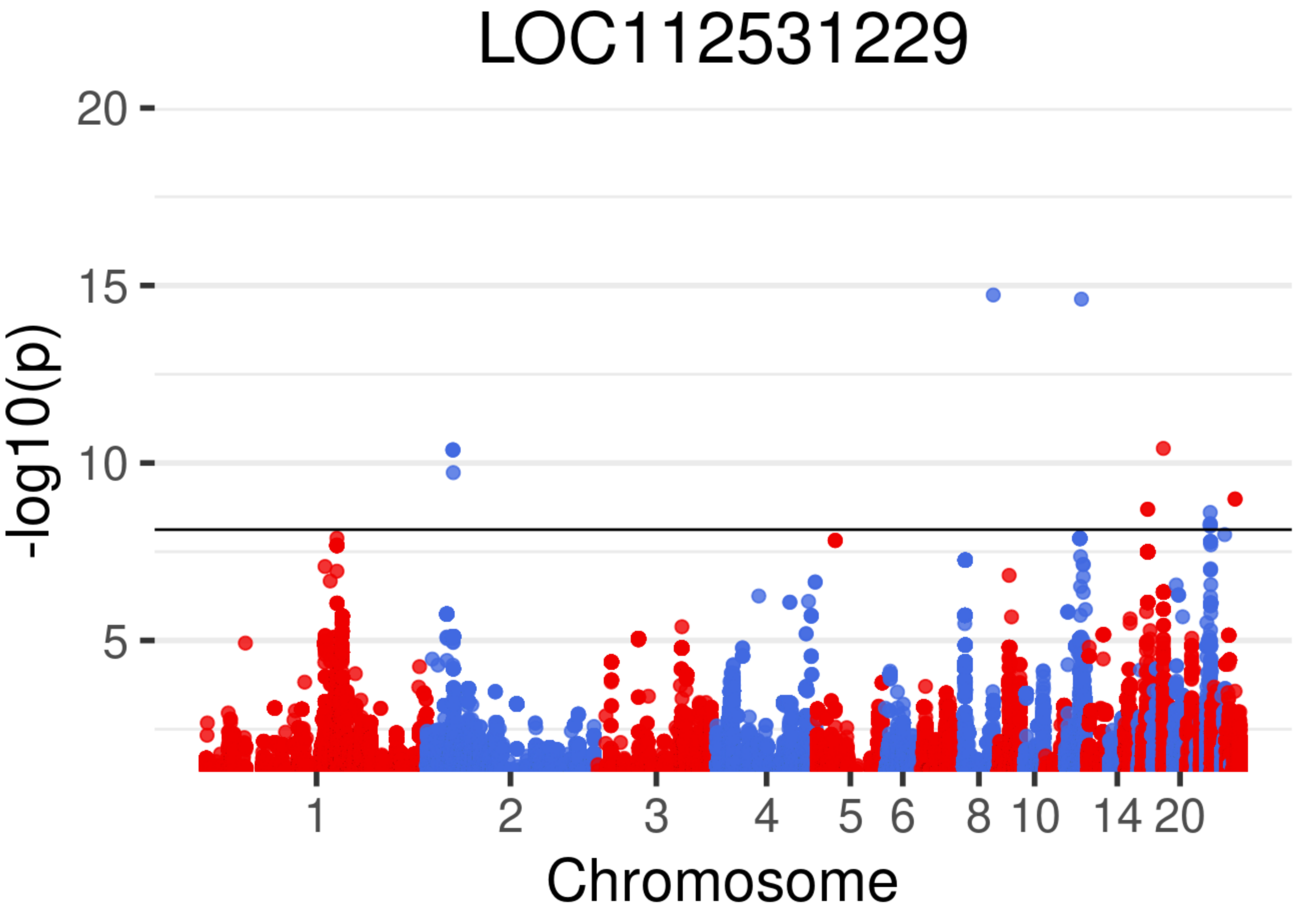
LOC112531229
-log10(p)
20
15
10
5
1
2
3
4
5
6
8
10
14
20
Chromosome

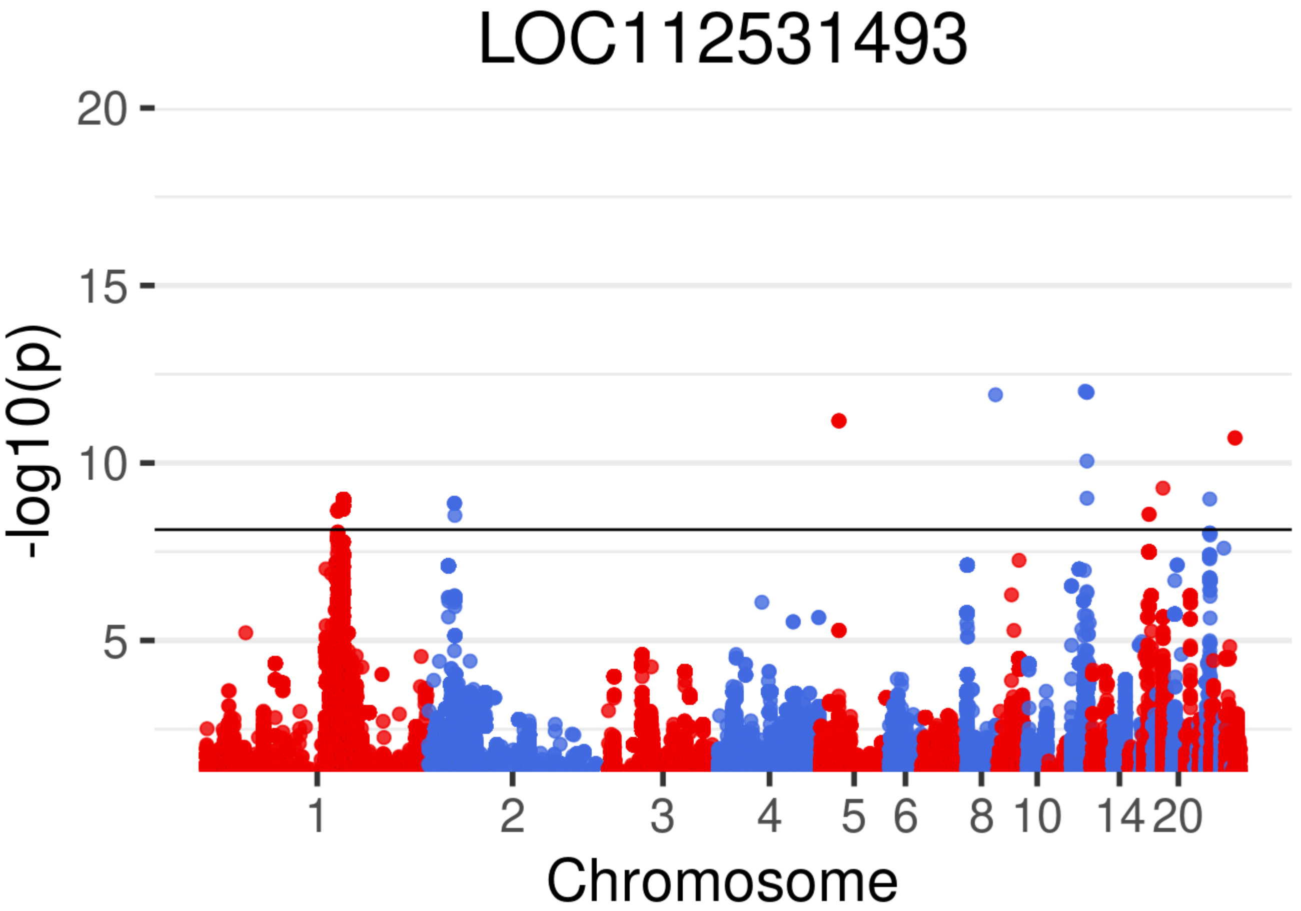
LOC112531493
-log10(p)
20
15
10
5
1
2
3
4
5
6
8
10
14
20
Chromosome

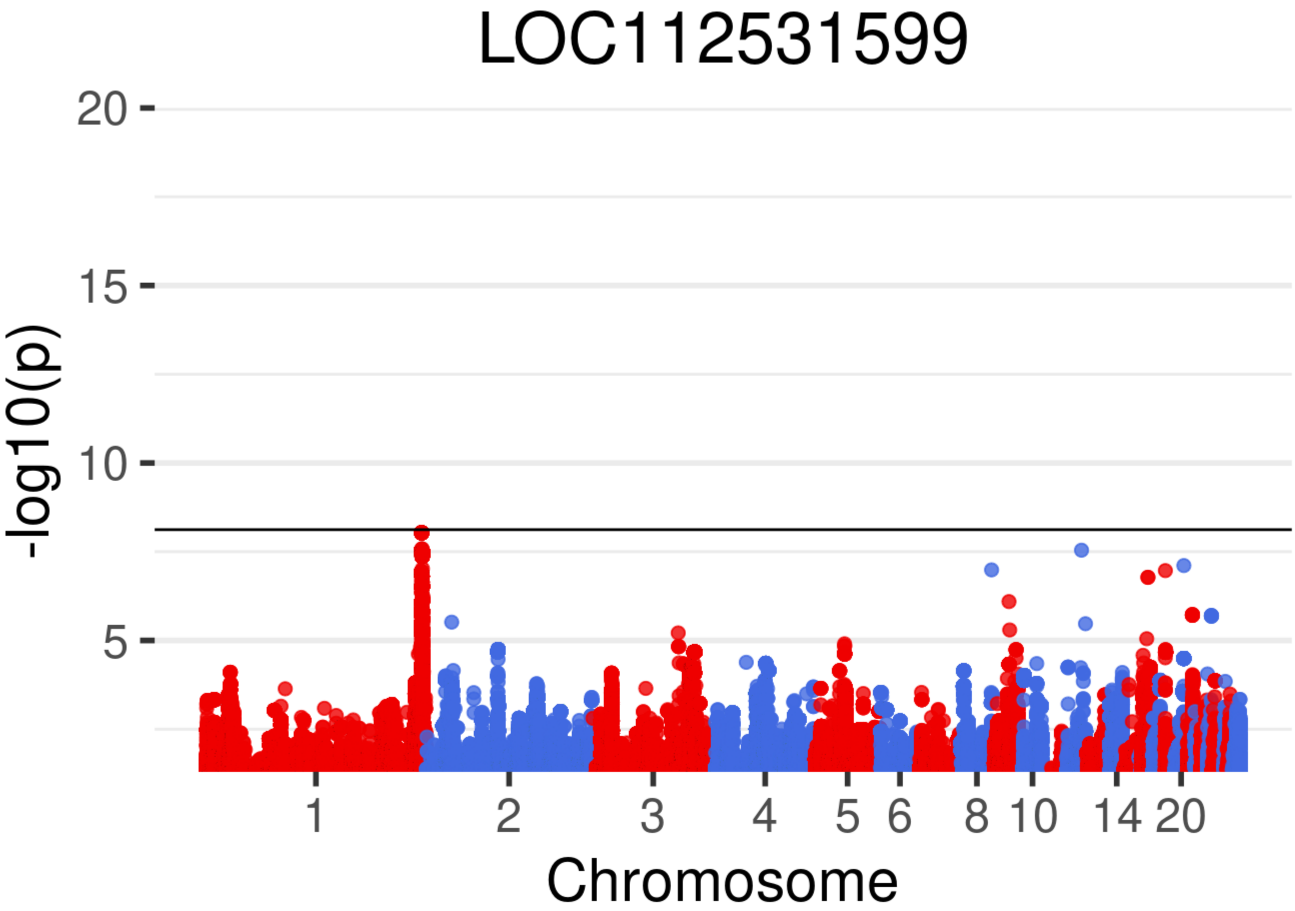

LOC112531599
20
15
10
5
-log10(p)
1
2
3
4
5
6
8
10
14
20
Chromosome

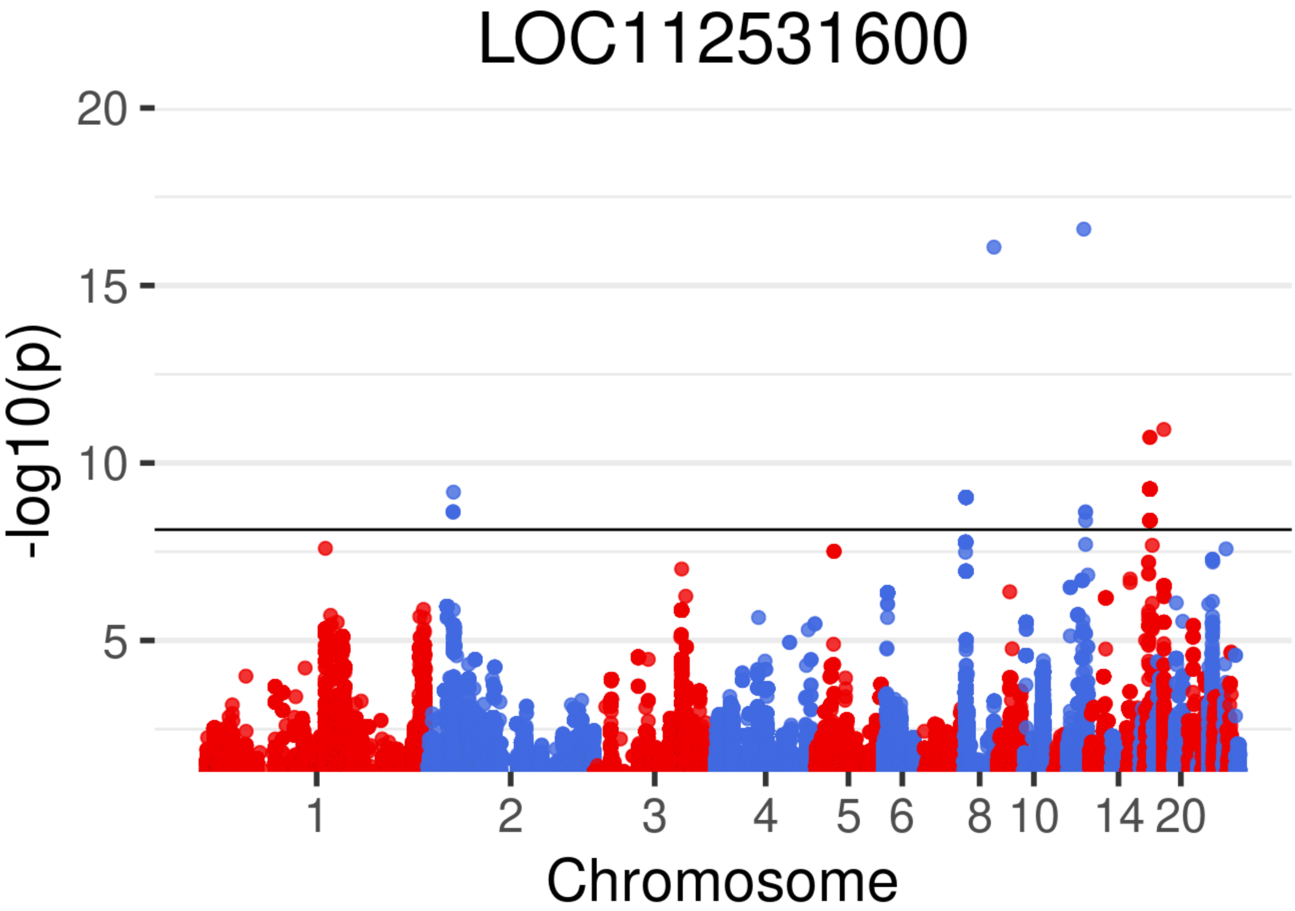
LOC112531600
20
15
10
5
-log10(p)
1
2
3
4
5
6
8
10
14
20
Chromosome

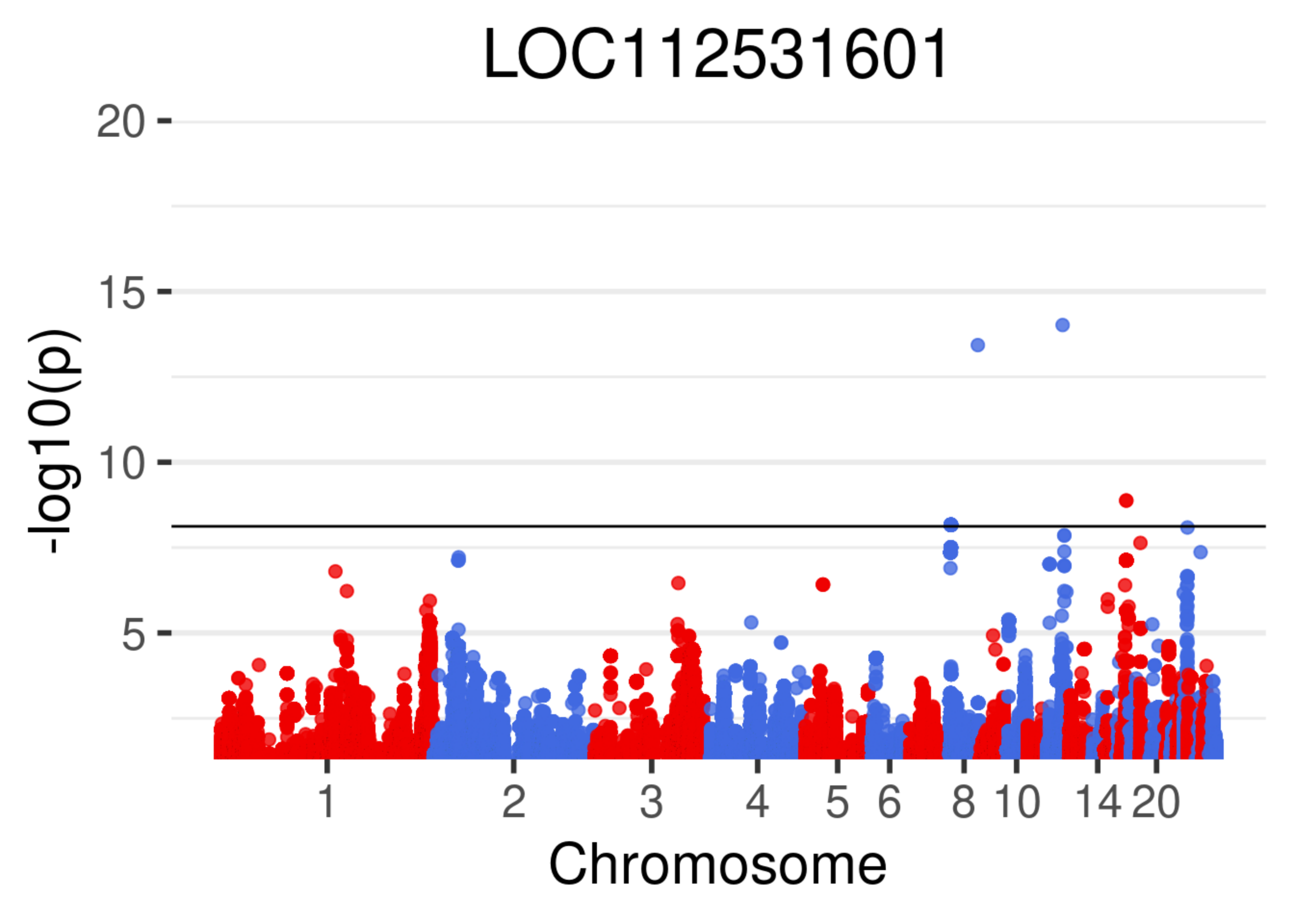
LOC112531601
-log10(p)
20
15
10
5
1
2
3
4
5
6
8
10
14
20
Chromosome

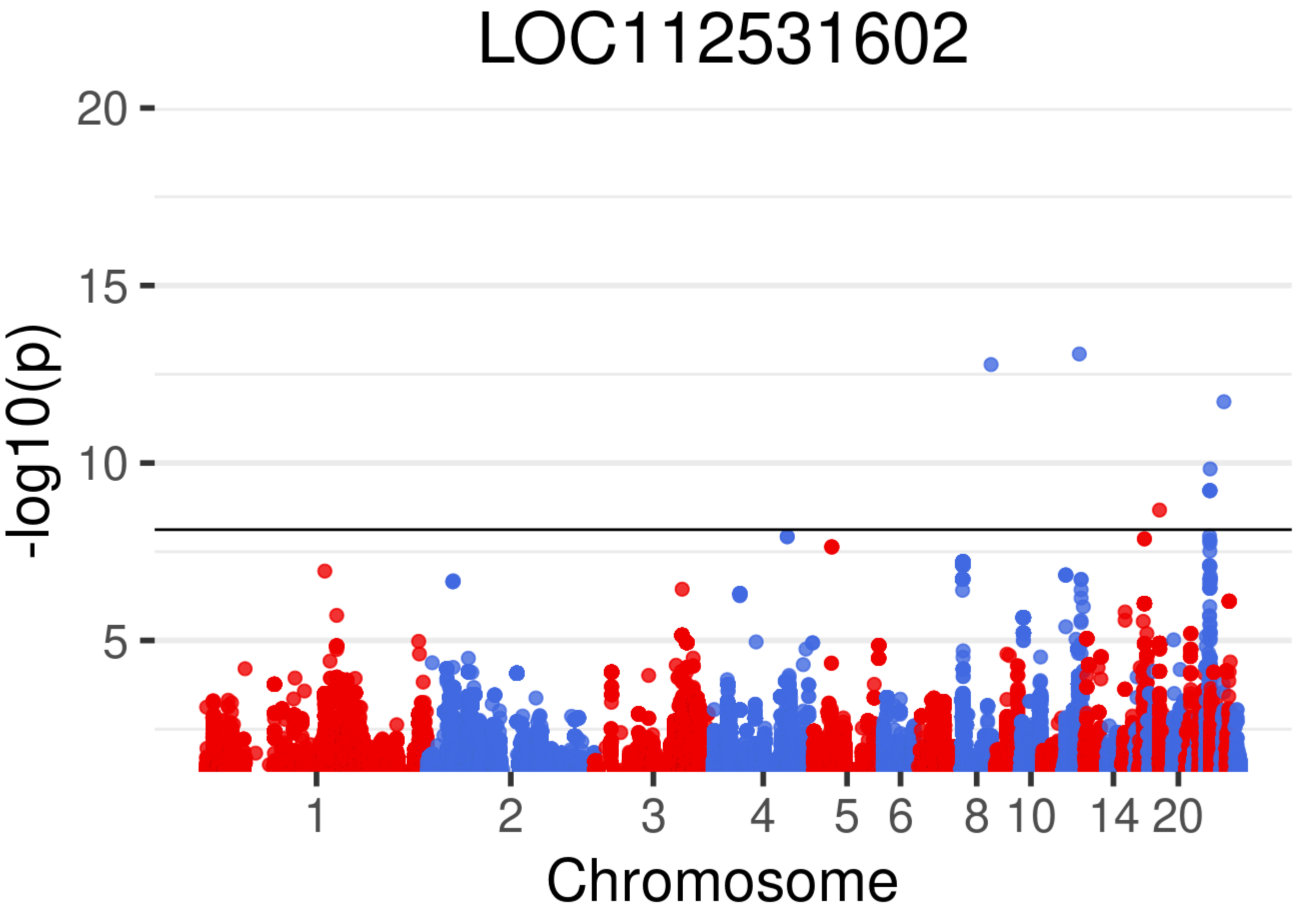

LOC112531602
-log10(p)
20
15
10
5
1
2
3
4
5
6
8
10
14
20
Chromosome

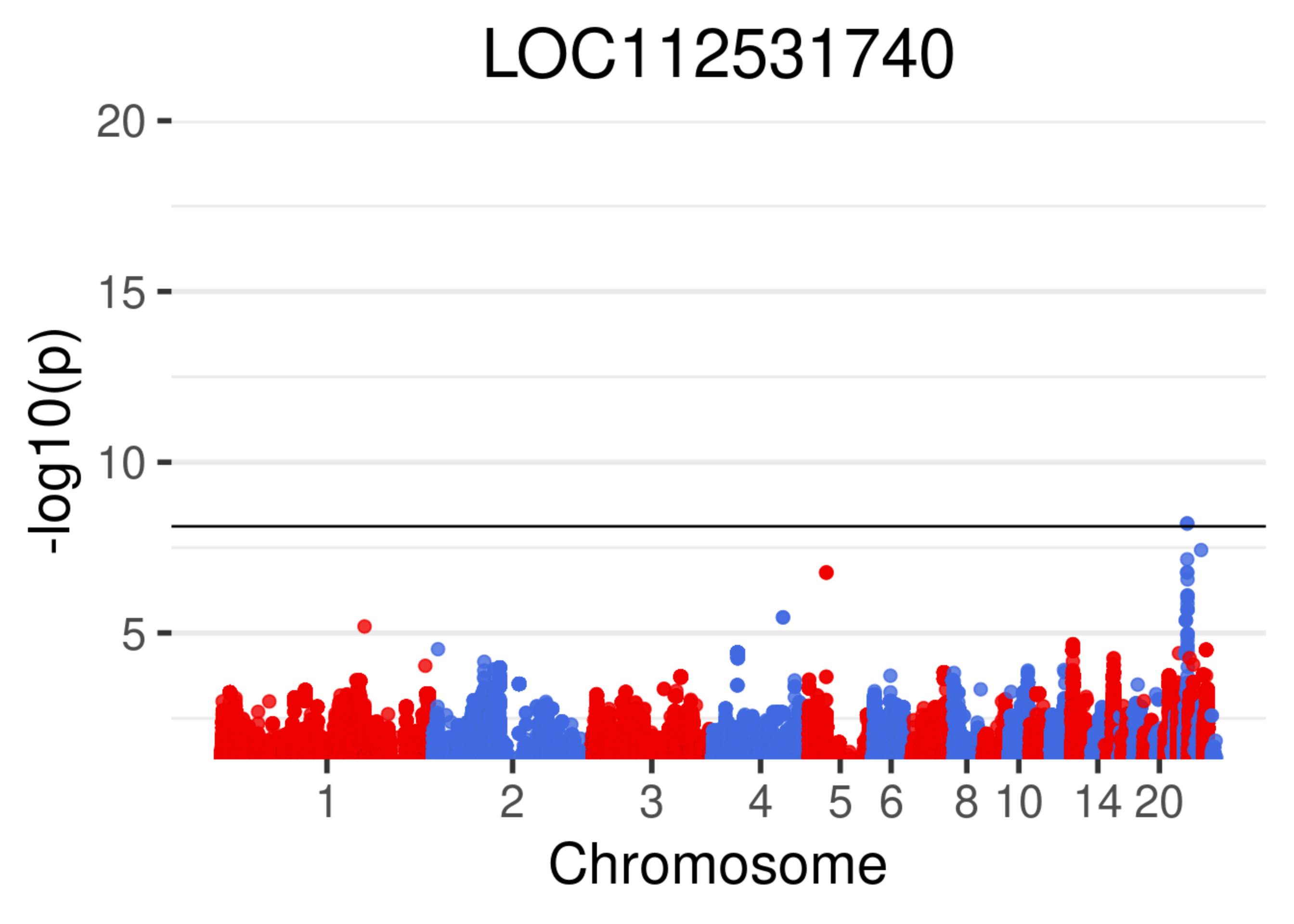

LOC112531740
20
15
10
5
-log10(p)
1
2
3
4
5
6
8
10
14
20
Chromosome

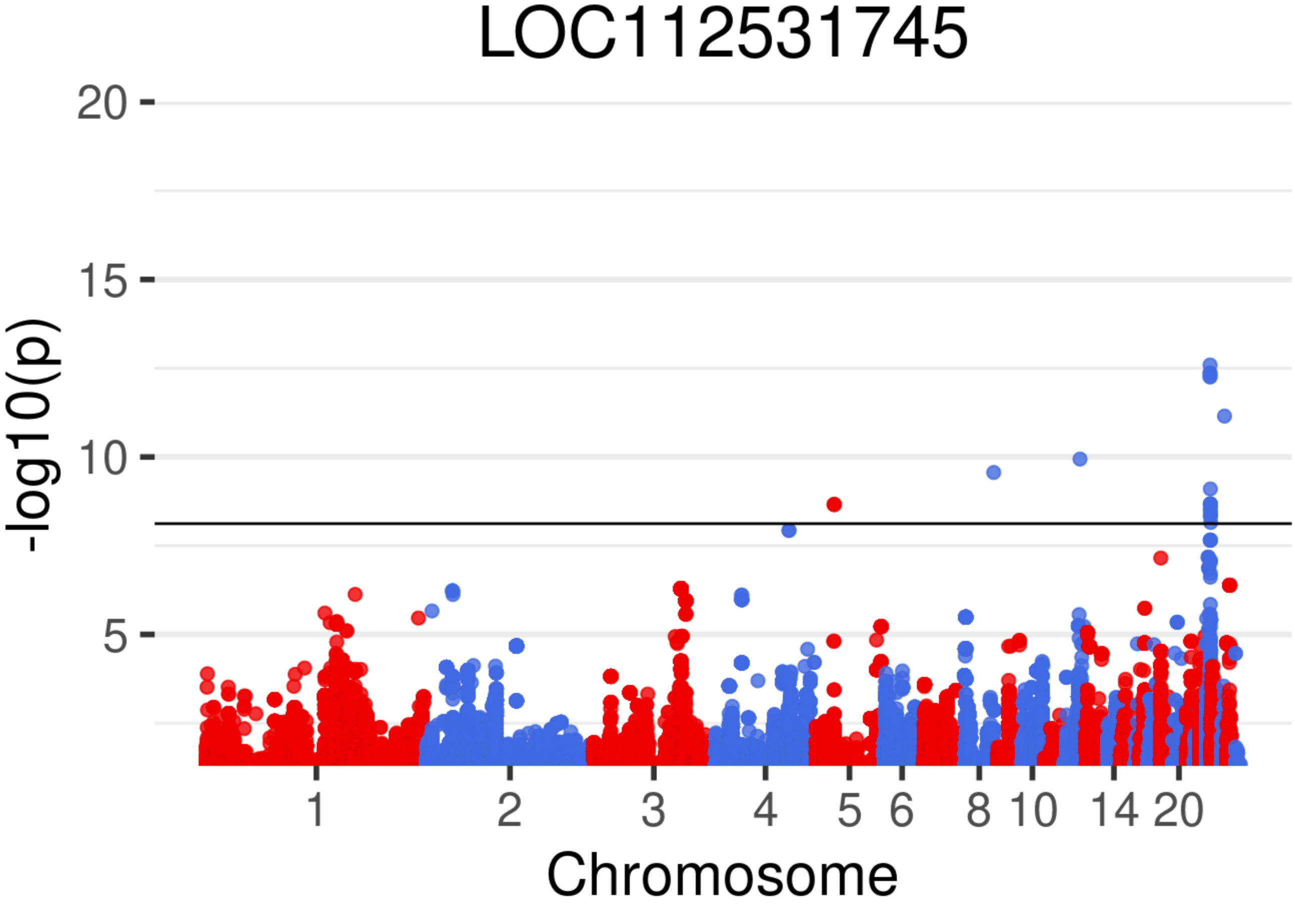

LOC112531745
-log10(p)
20
15
10
5
1
2
3
4
5
6
8
10
14
20
Chromosome

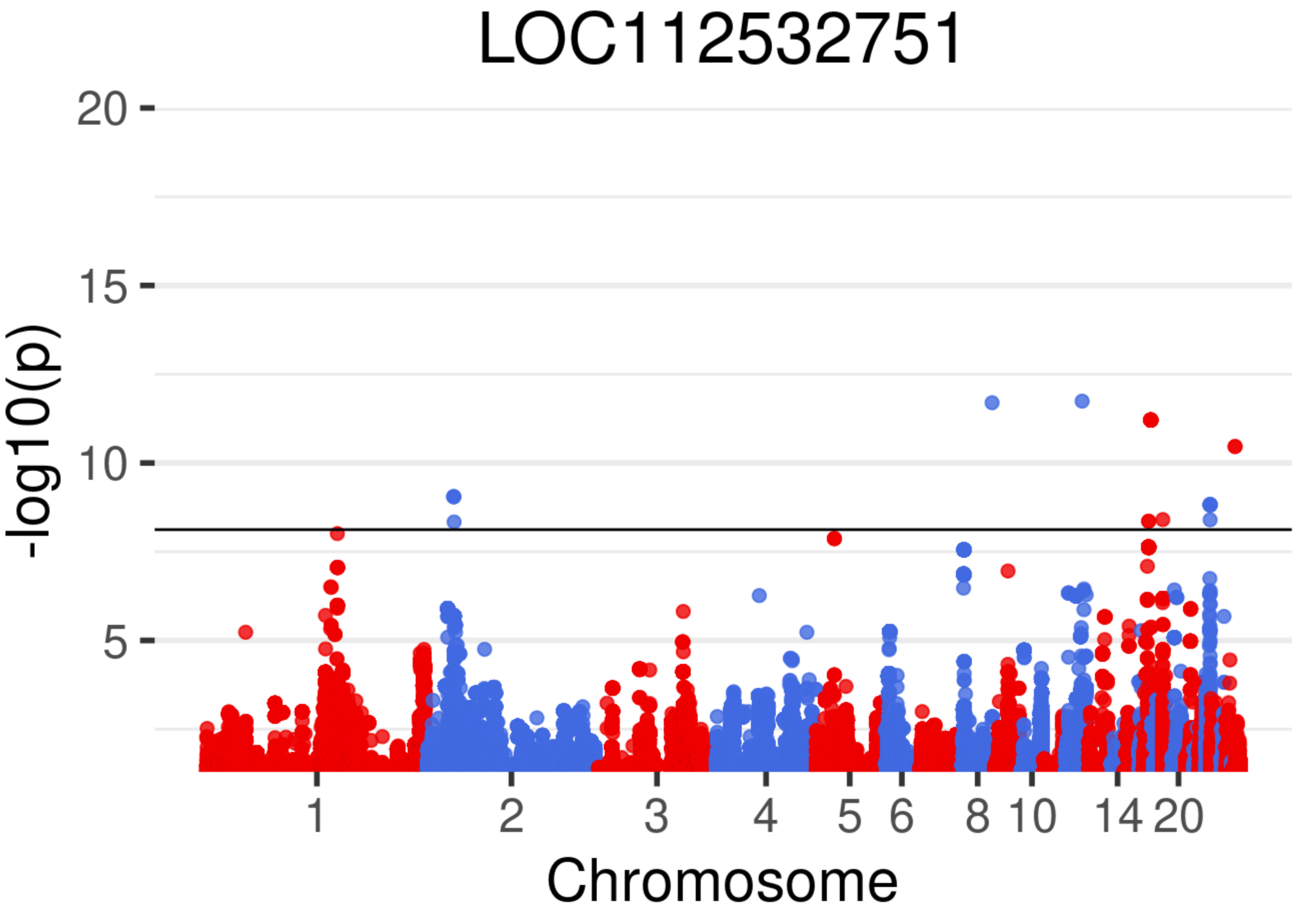
LOC112532751
20
15
10
5
-log10(p)
1
2
3
4
5
6
8
10
14
20
Chromosome

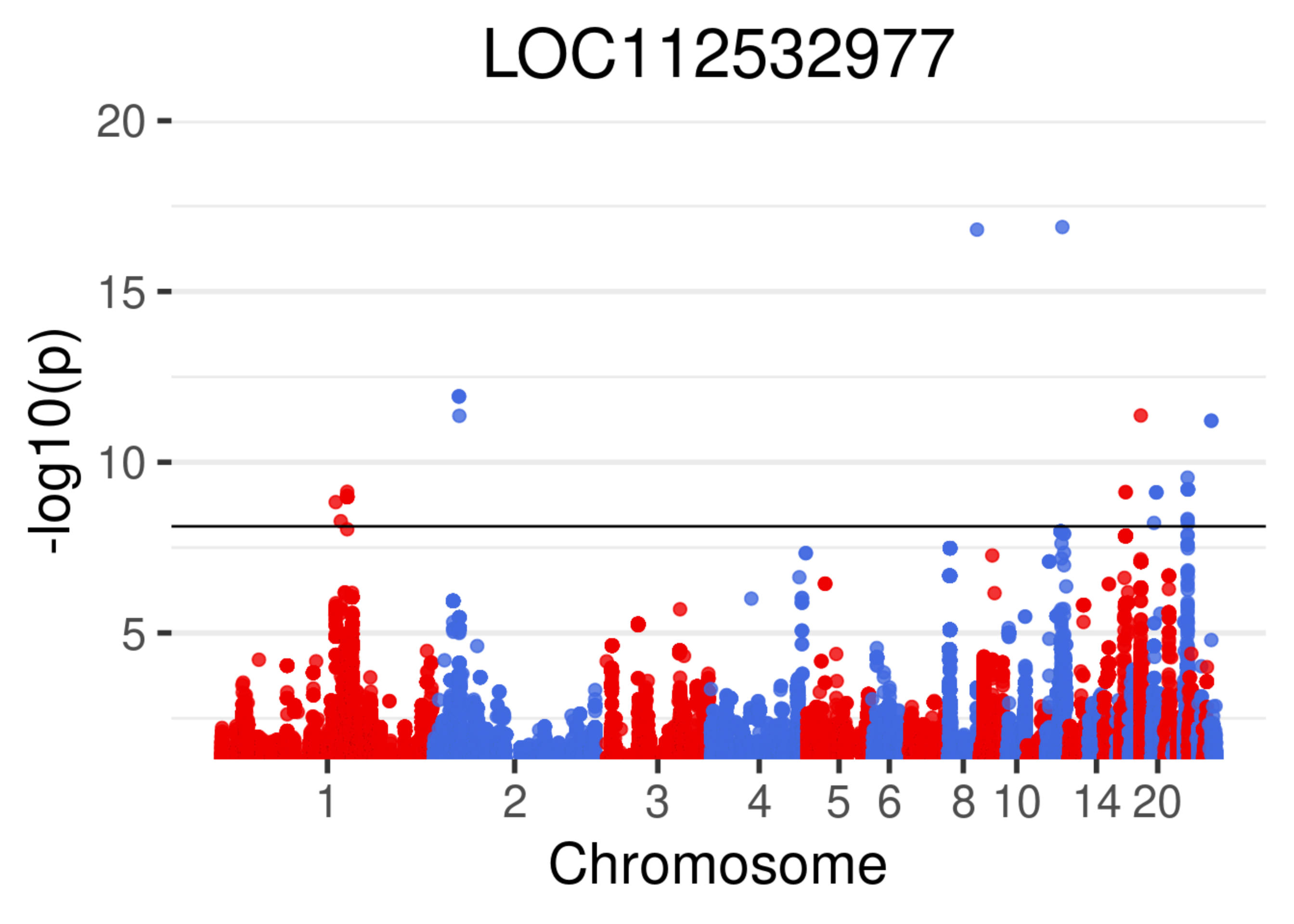
LOC112532977
20
15
10
5
-log10(p)
1
2
3
4
5
6
8
10
14
20
Chromosome

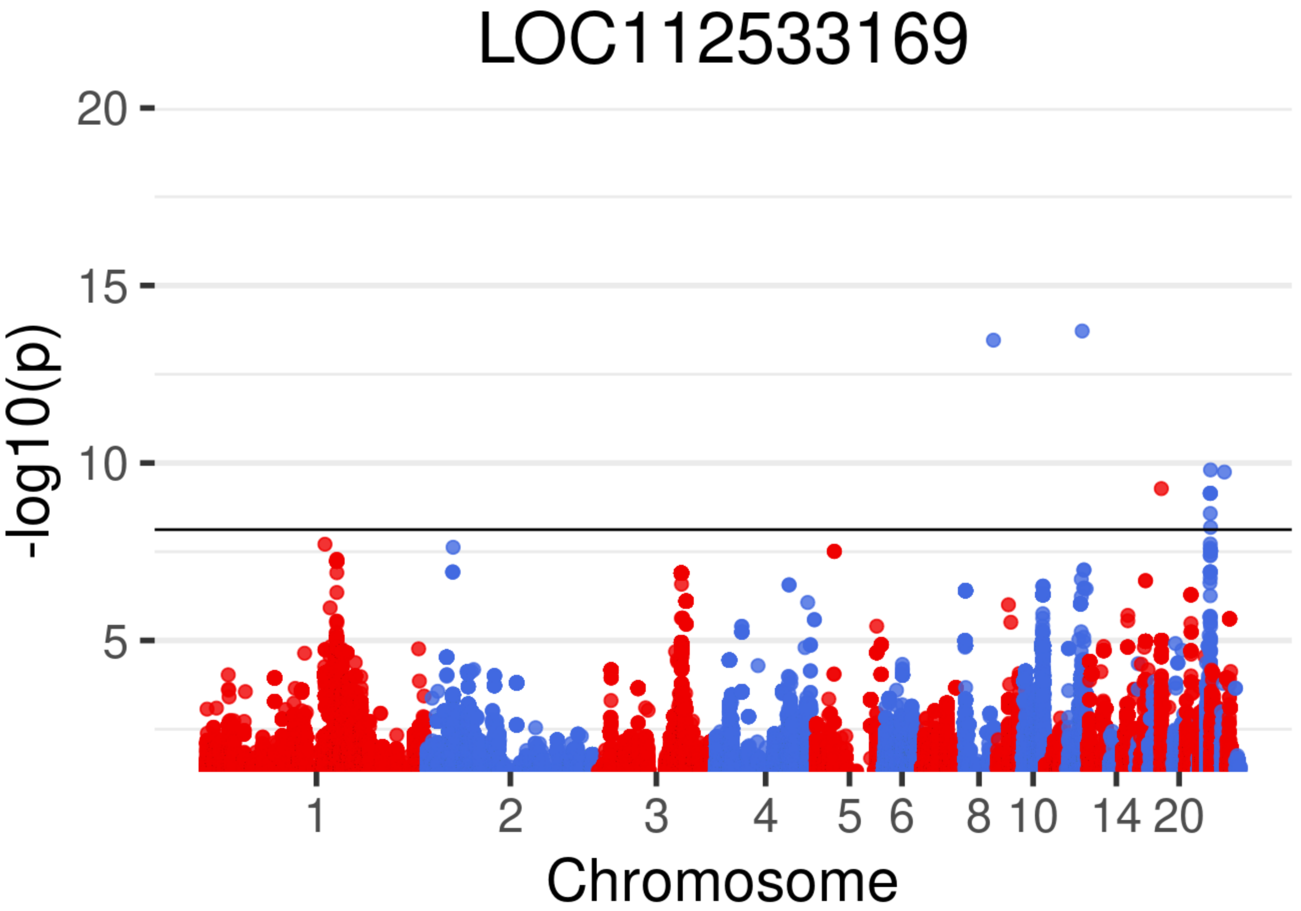
LOC112533169
-log10(p)
20
15
10
5
1
2
3
4
5
6
8
10
14
20
Chromosome

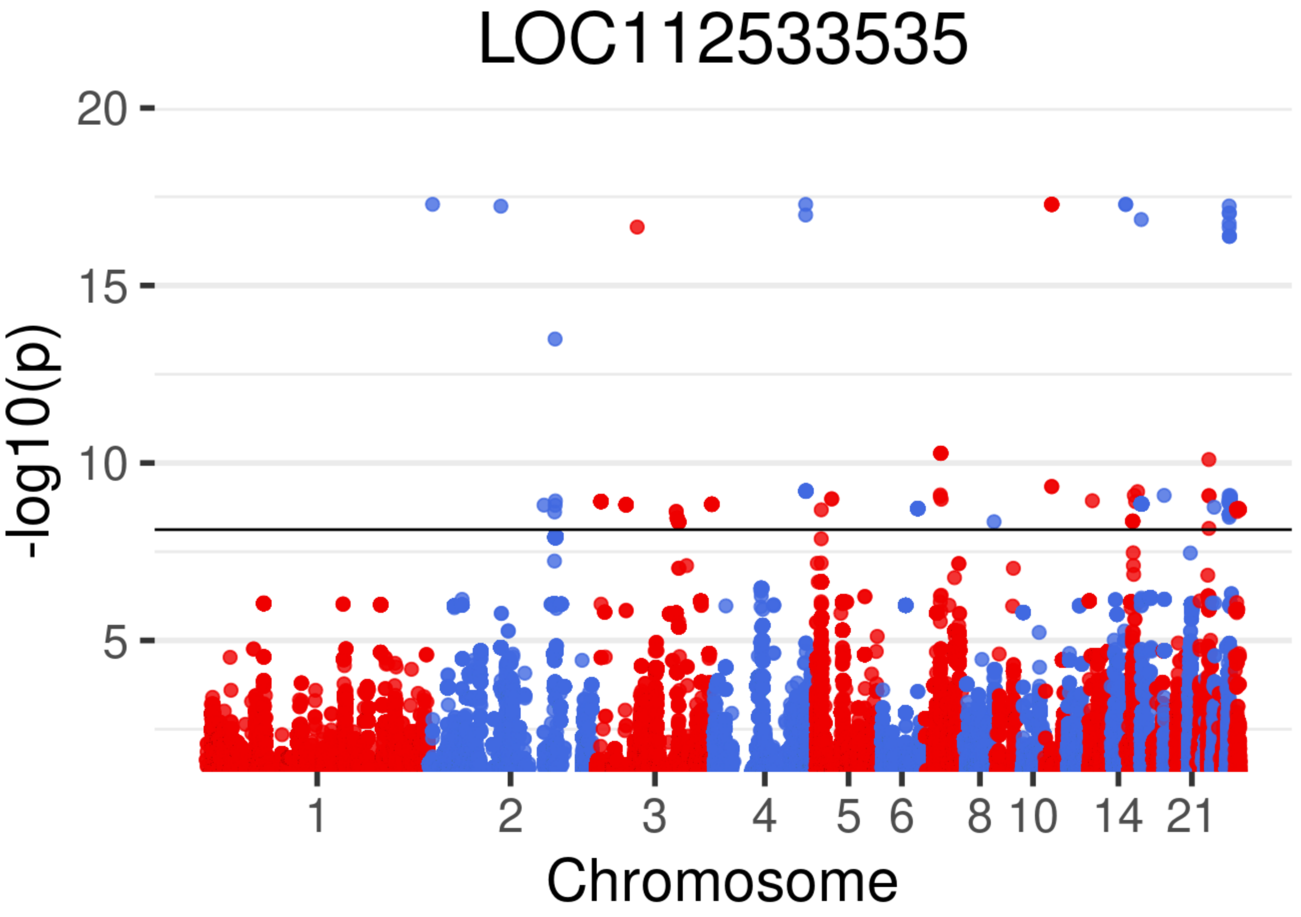
LOC112533535
20
15
10
5
-log10(p)
1
2
3
4
5
6
8
10
14
21
Chromosome

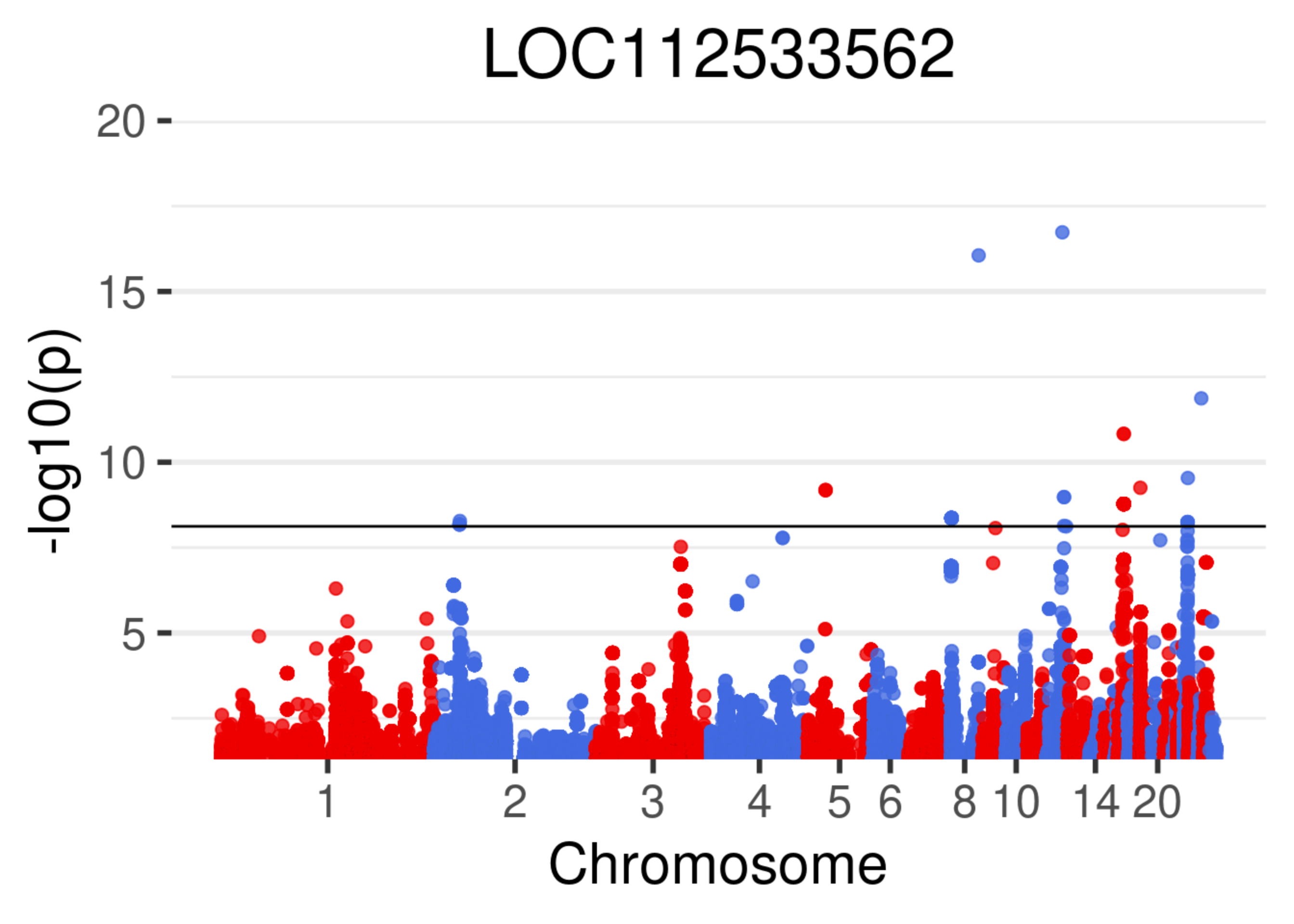

LOC112533562
20
15
10
5
-log10(p)
1
2
3
4
5
6
8
10
14
20
Chromosome

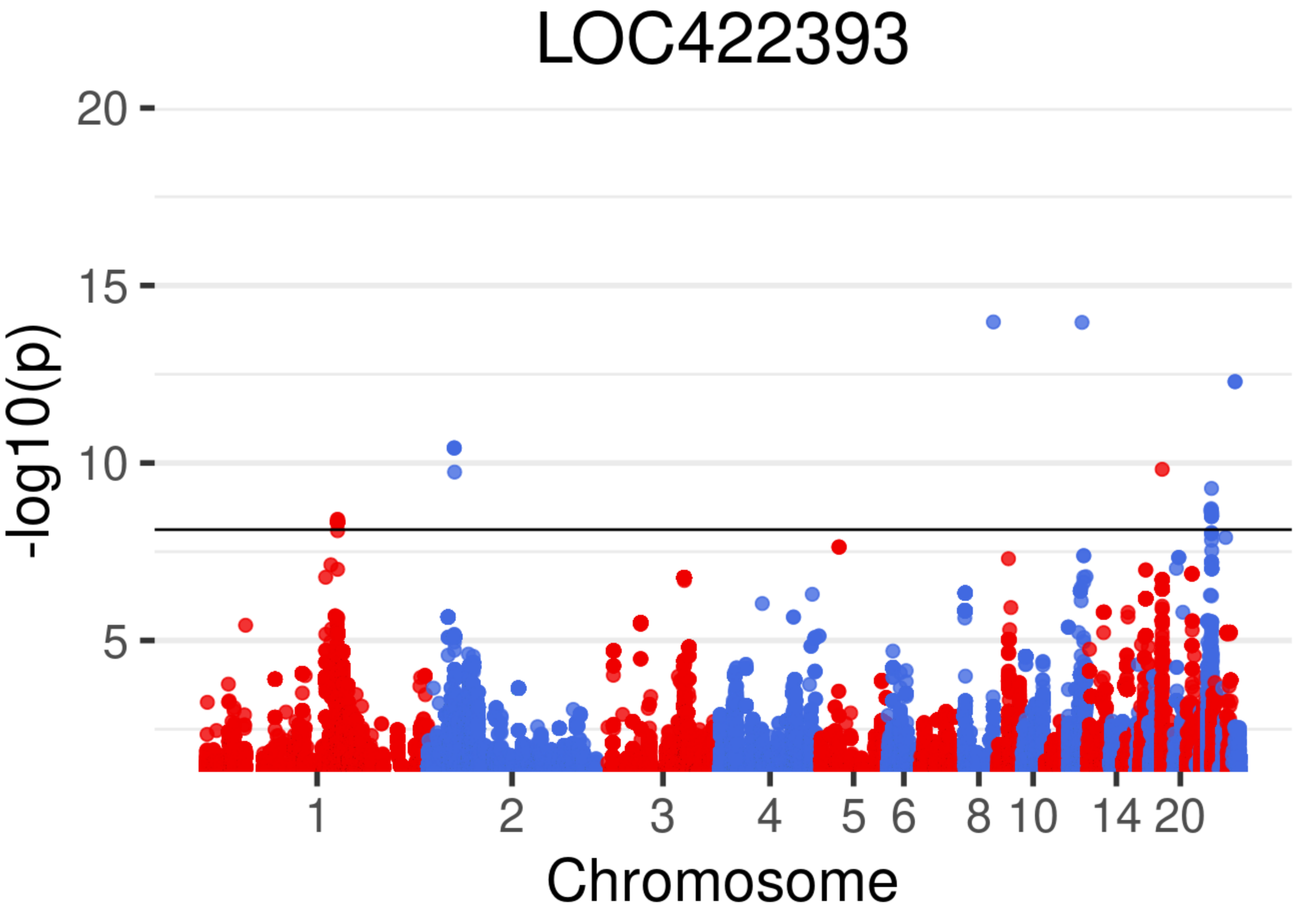
LOC422393
20
15
10
5
-log10(p)
1
2
3
4
5
6
8
10
14
20
Chromosome

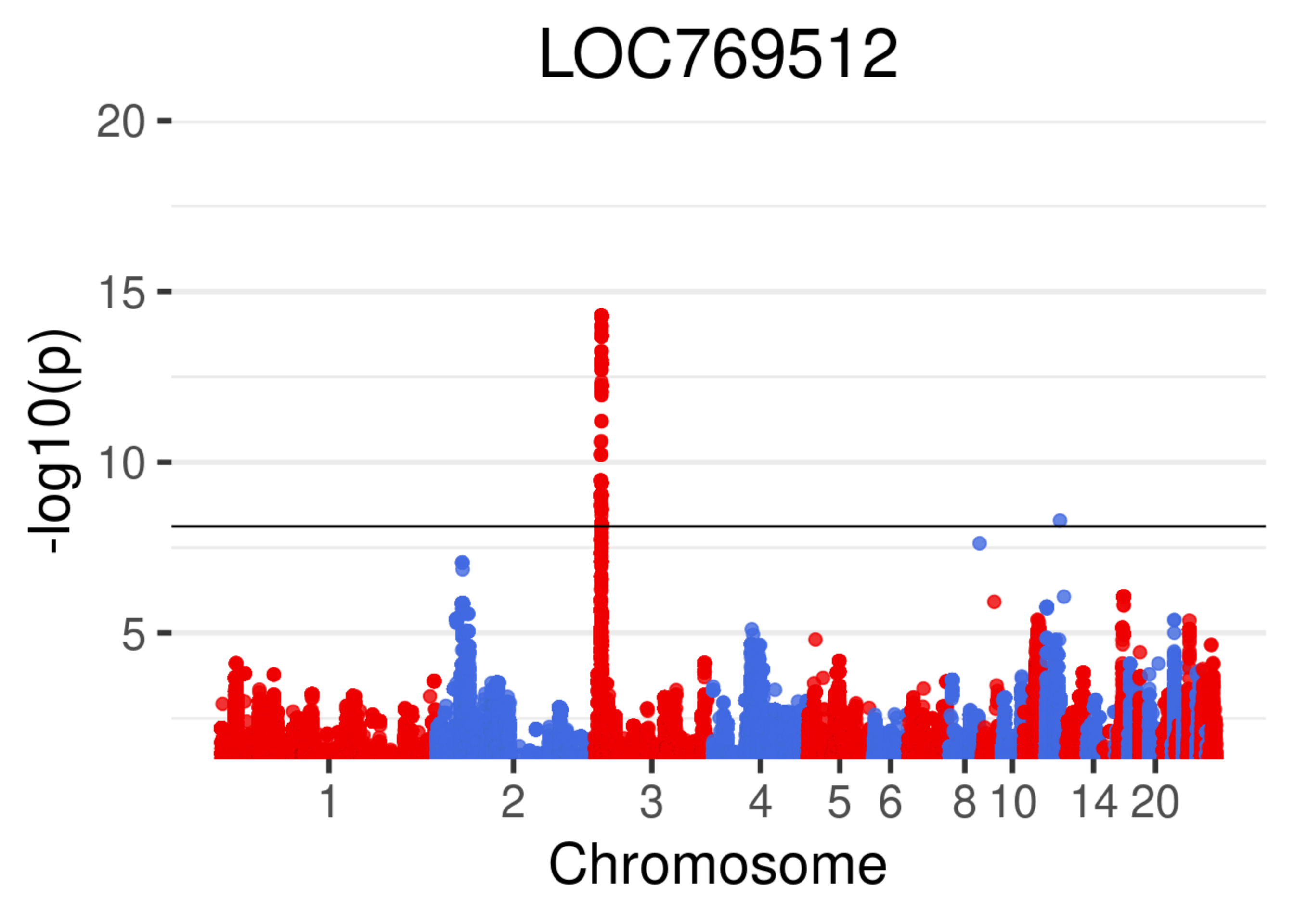
LOC769512
20
15
10
5
-log10(p)
1
2
3
4
5
6
8
10
14
20
Chromosome

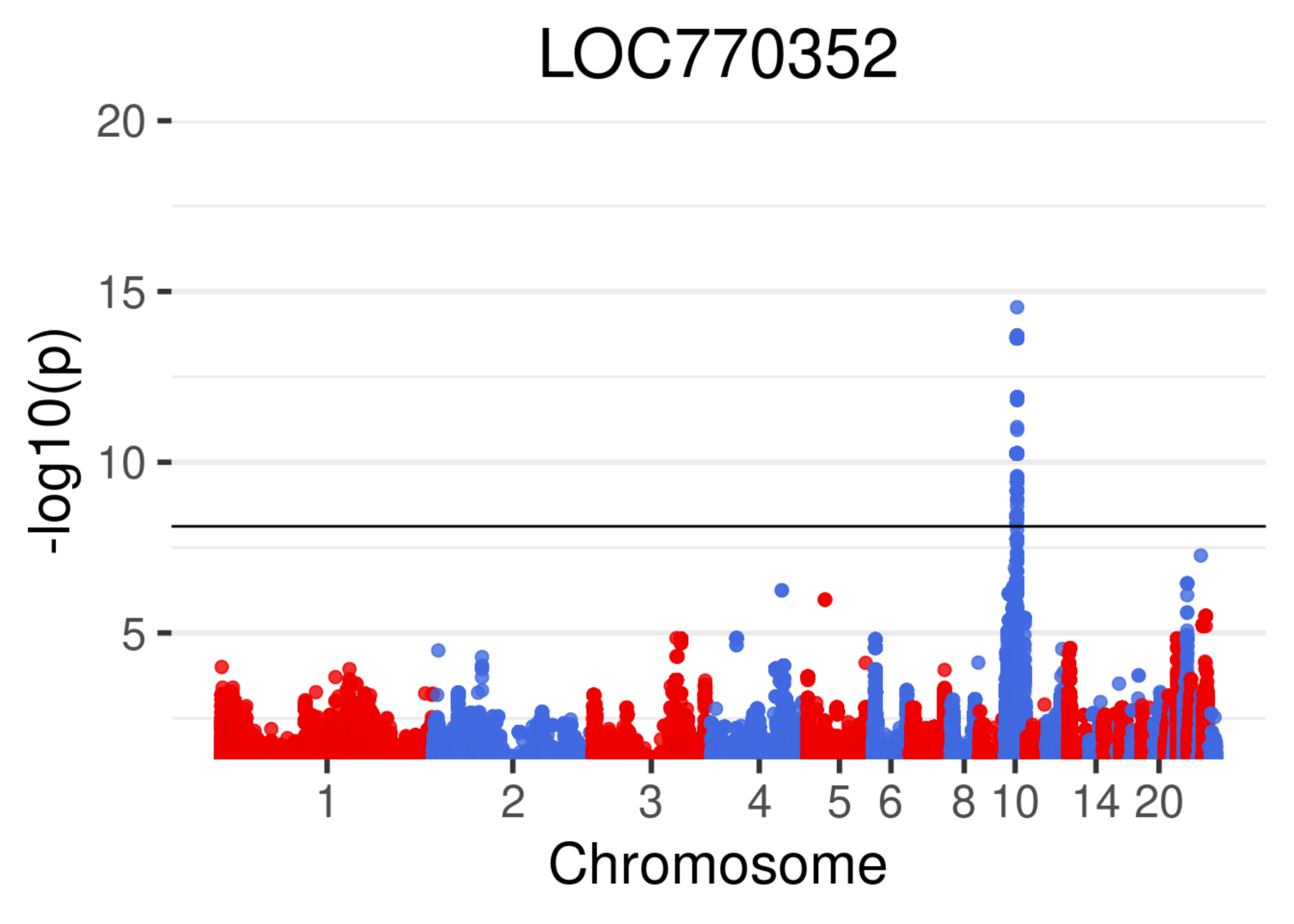
LOC770352
20
15
10
5
-log10(p)
1
2
3
4
5
6
8
10
14
20
Chromosome

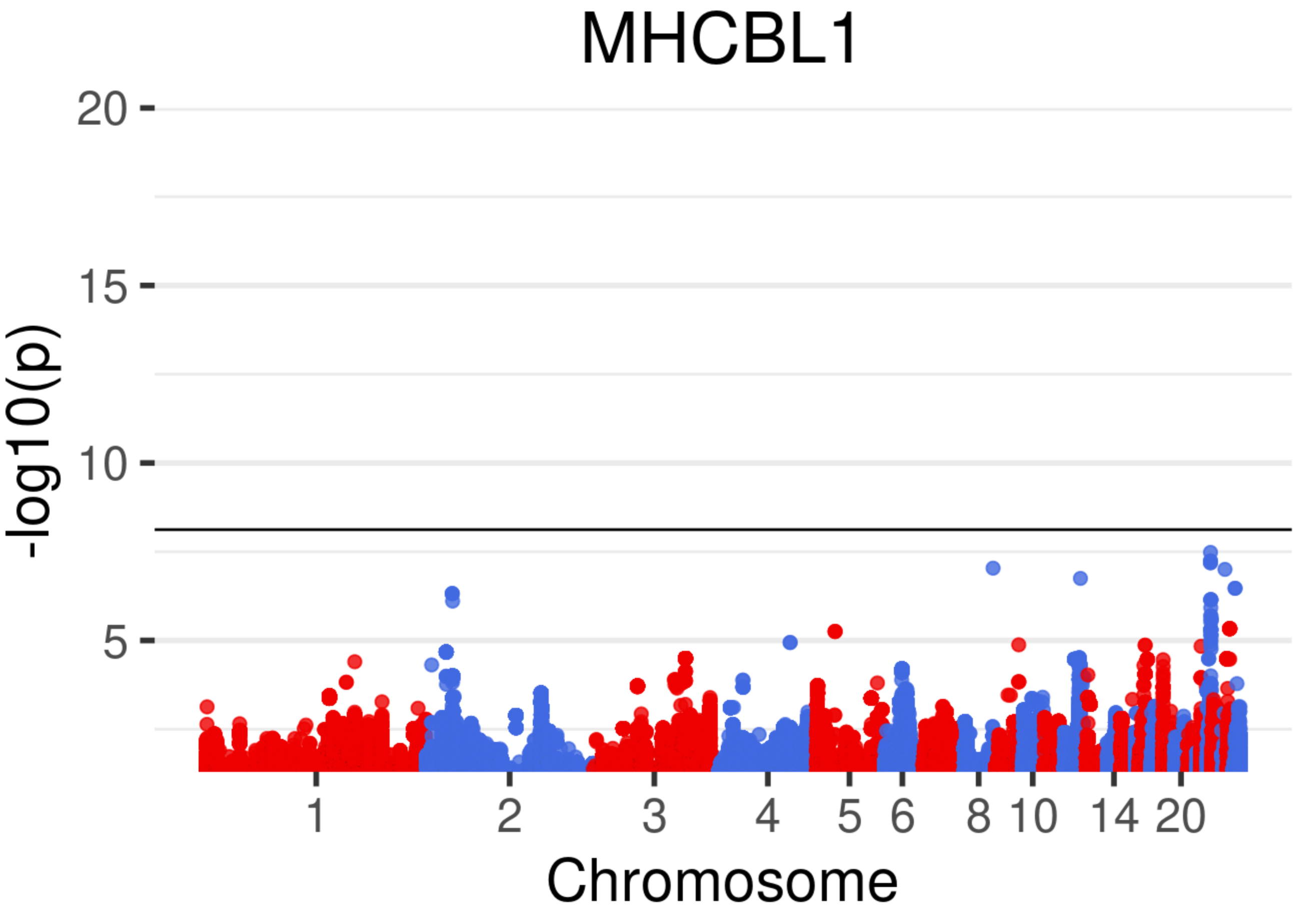
MHCBL1
20
15
10
5
-log10(p)
1
2
3
4
5
6
8
10
14
20
Chromosome

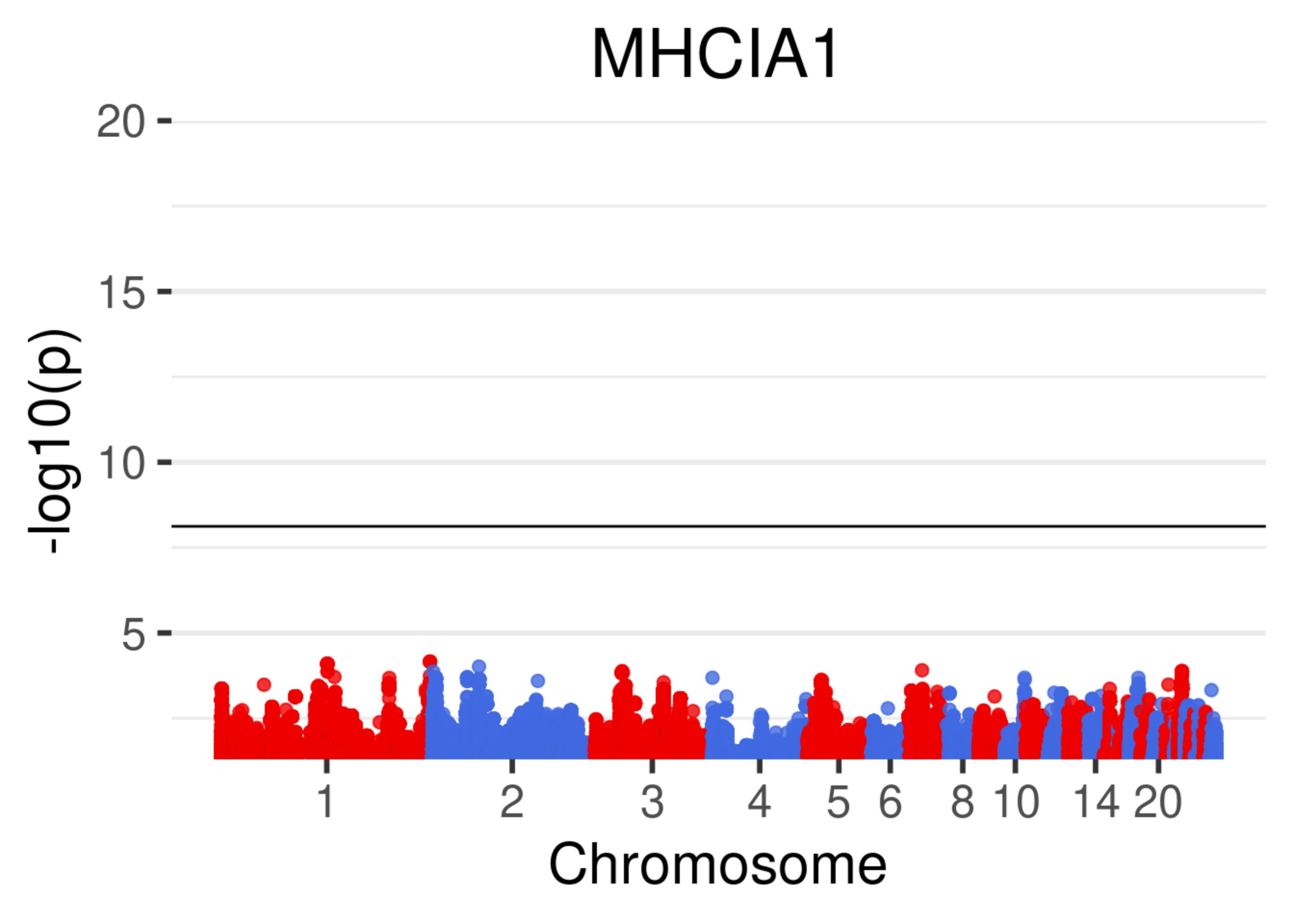
MHCIA1
20
15
10
5
-log10(p)
1
2
3
4
5
6
8
10
14
20
Chromosome

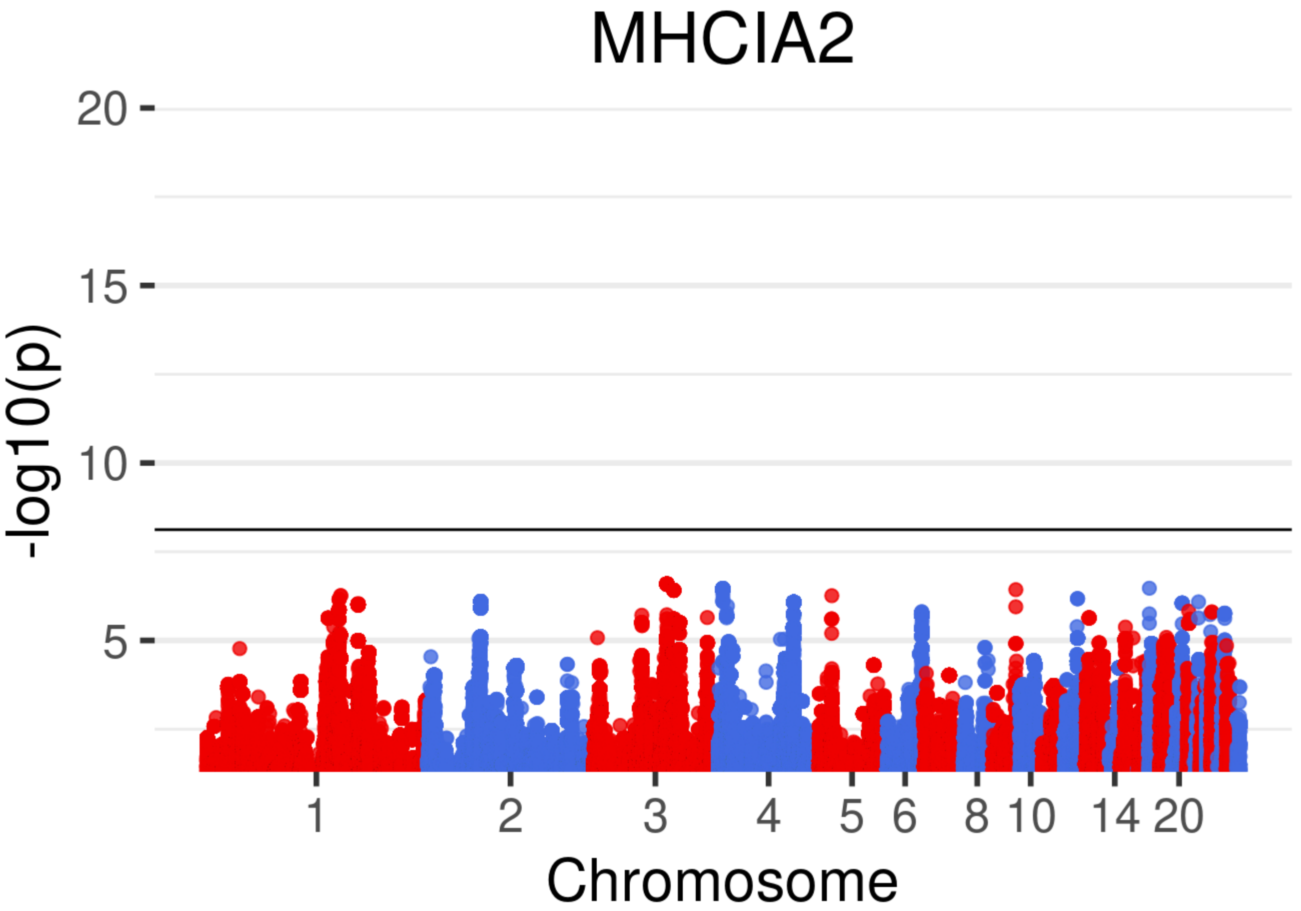
MHCIA2
20
15
10
5
-log10(p)
1
2
3
4
5
6
8
10
14
20
Chromosome

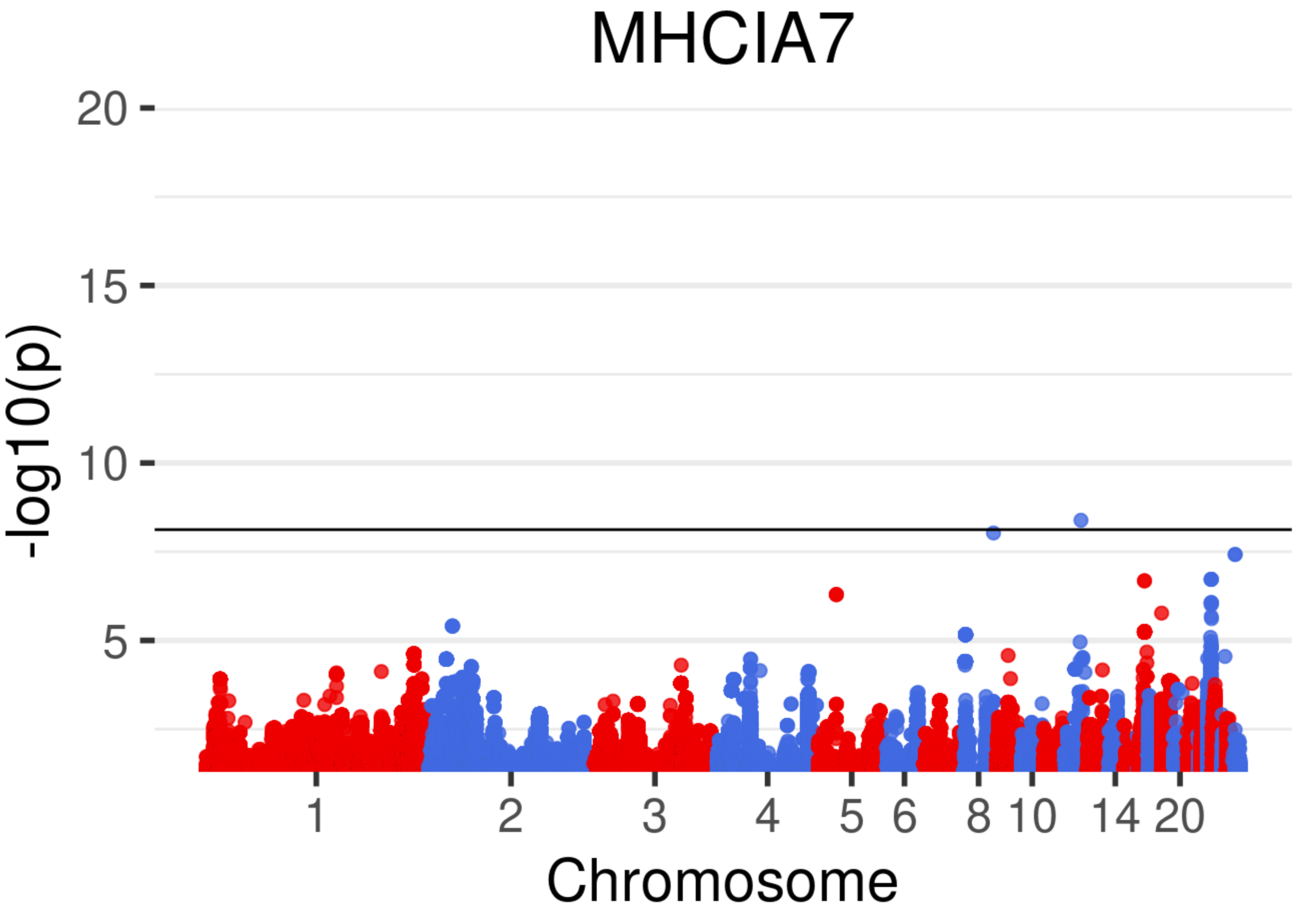
MHCIA7
20
15
10
5
-log10(p)
1
2
3
4
5
6
8
10
14
20
Chromosome

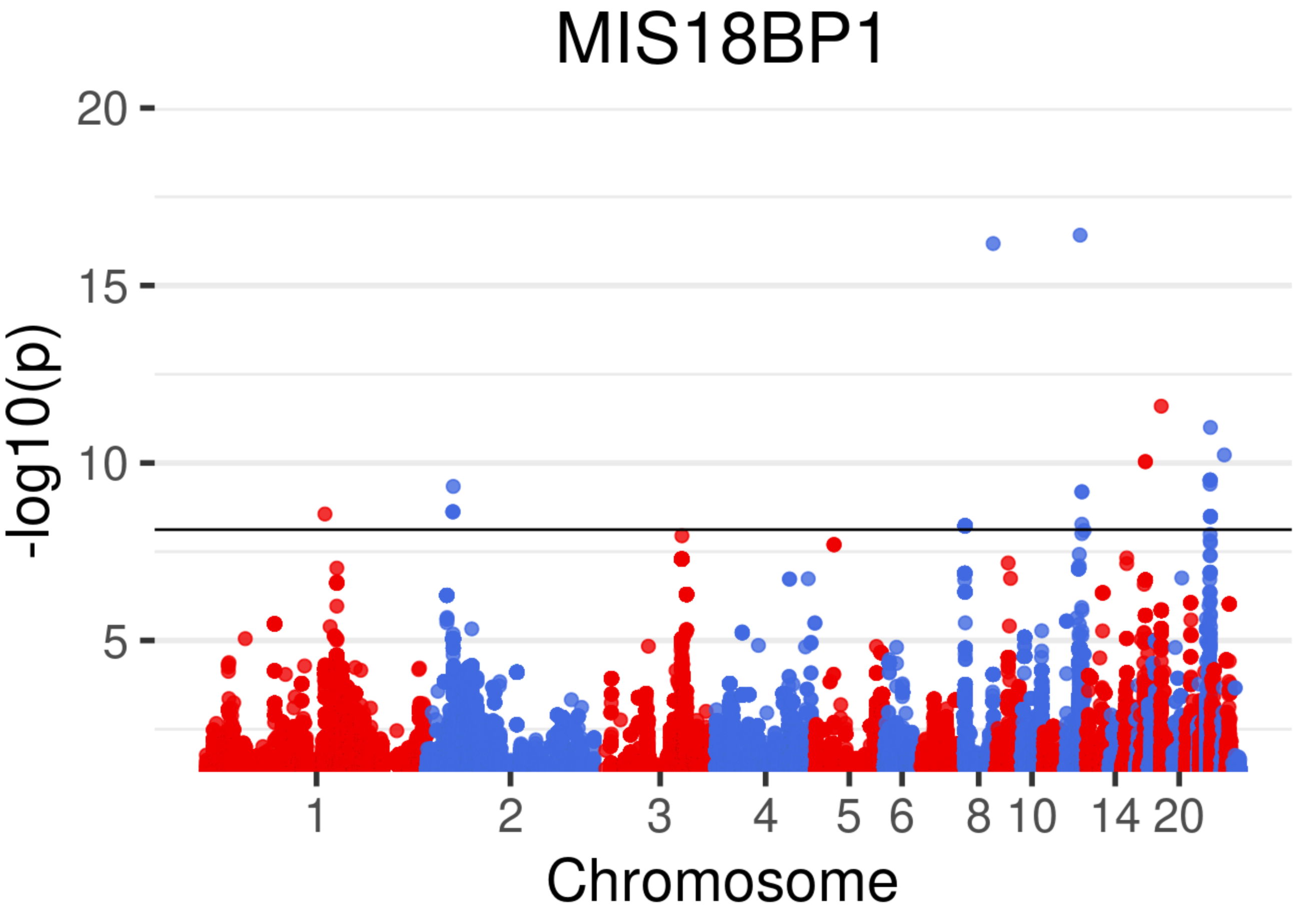
MIS18BP1
20
15
10
5
-log10(p)
1
2
3
4
5
6
8
10
14
20
Chromosome

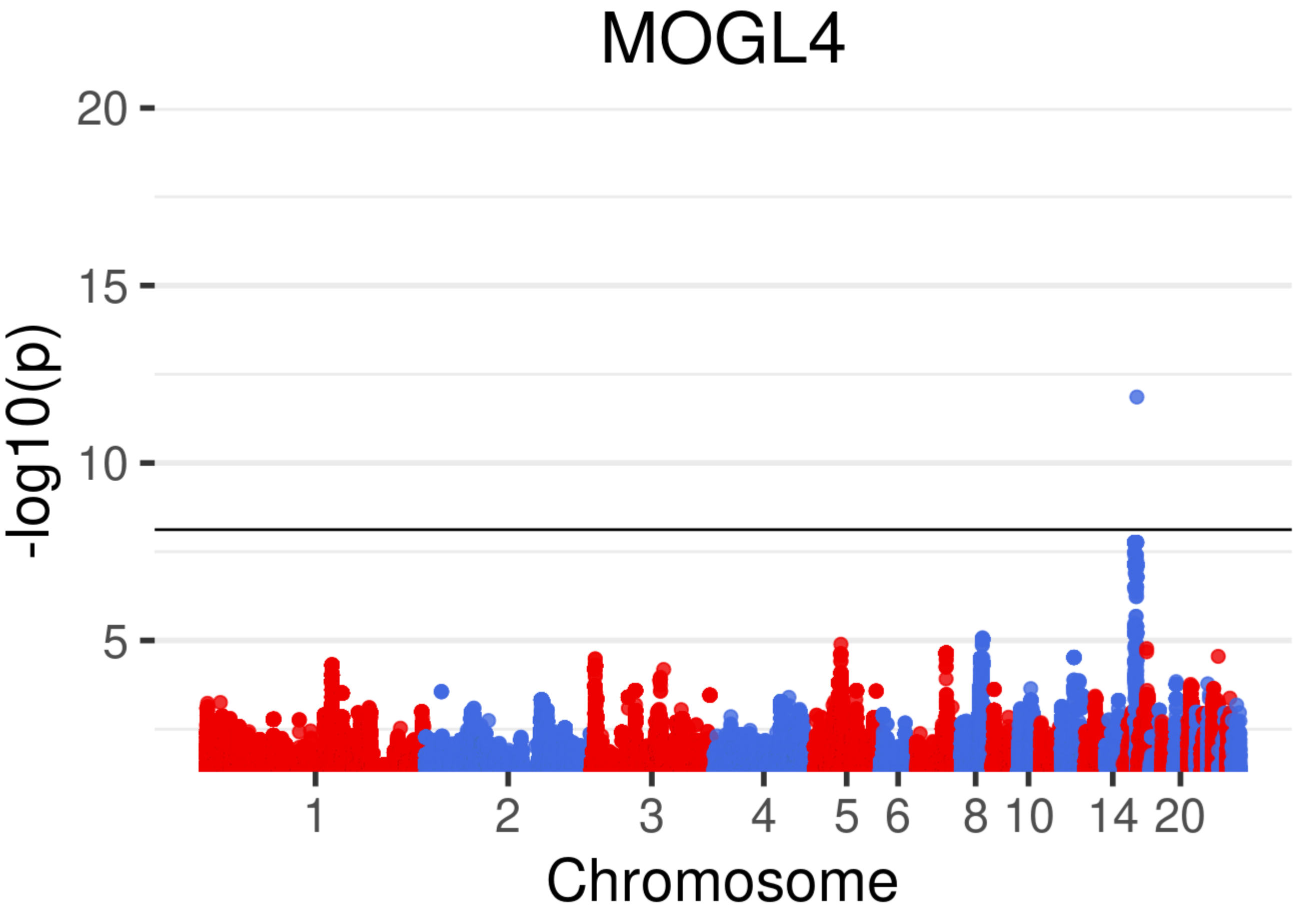
MOGL4
20
15
10
5
-log10(p)
1
2
3
4
5
6
8
10
14
20
Chromosome

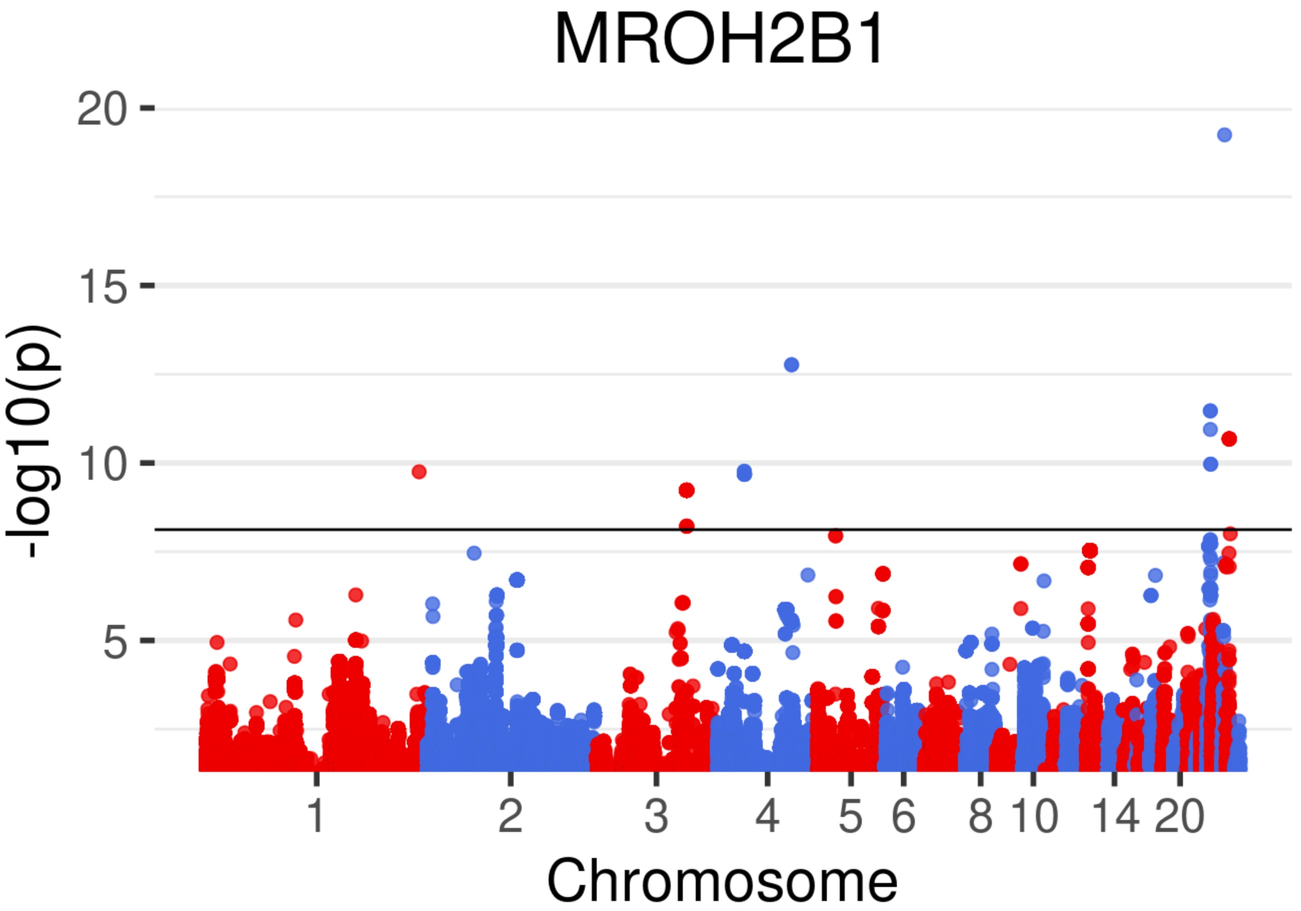

MROH2B1
20
15
10
5
-log10(p)
1
2
3
4
5
6
8
10
14
20
Chromosome

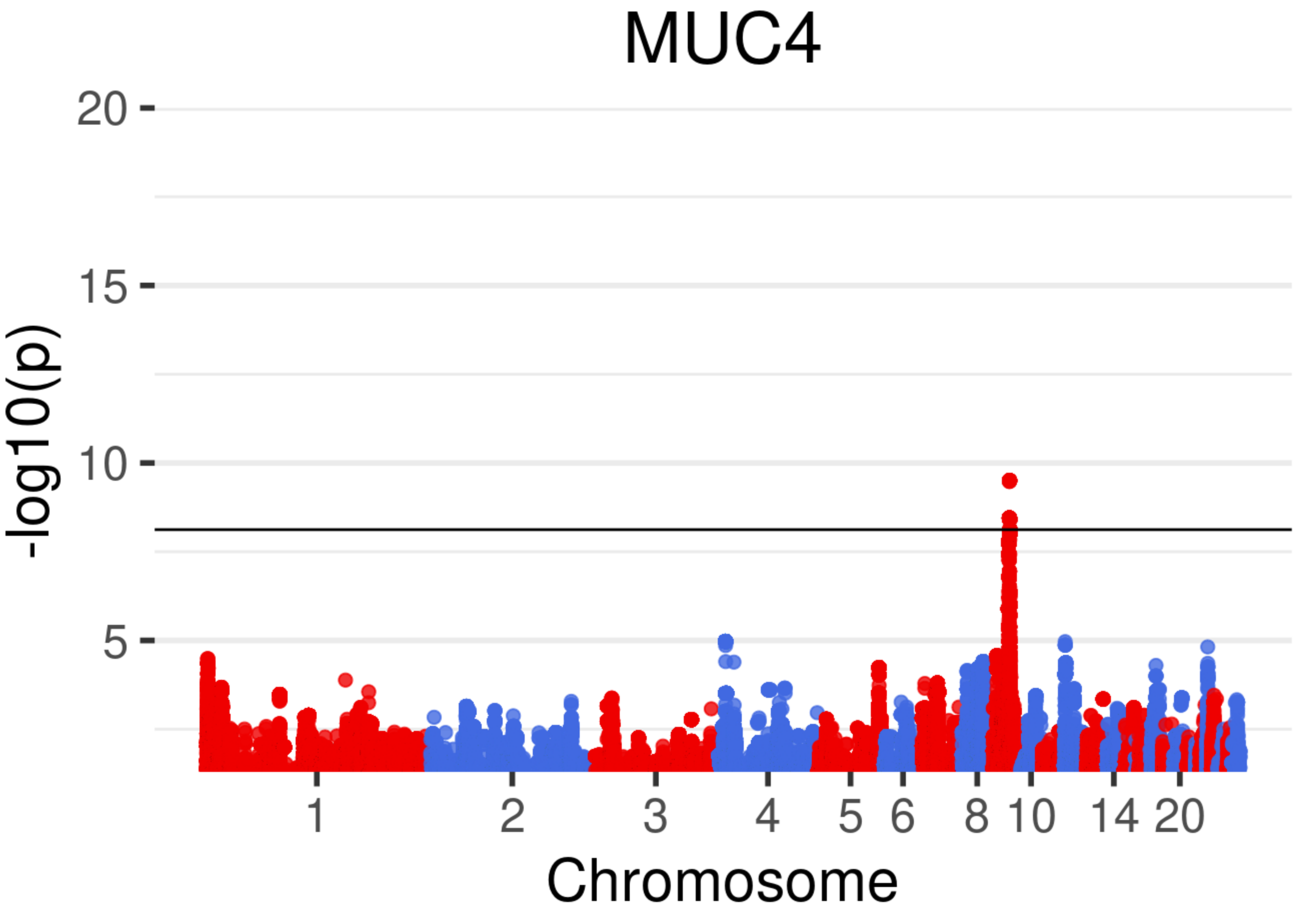
MUC4
20
15
10
5
-log10(p)
1
2
3
4
5
6
8
10
14
20
Chromosome

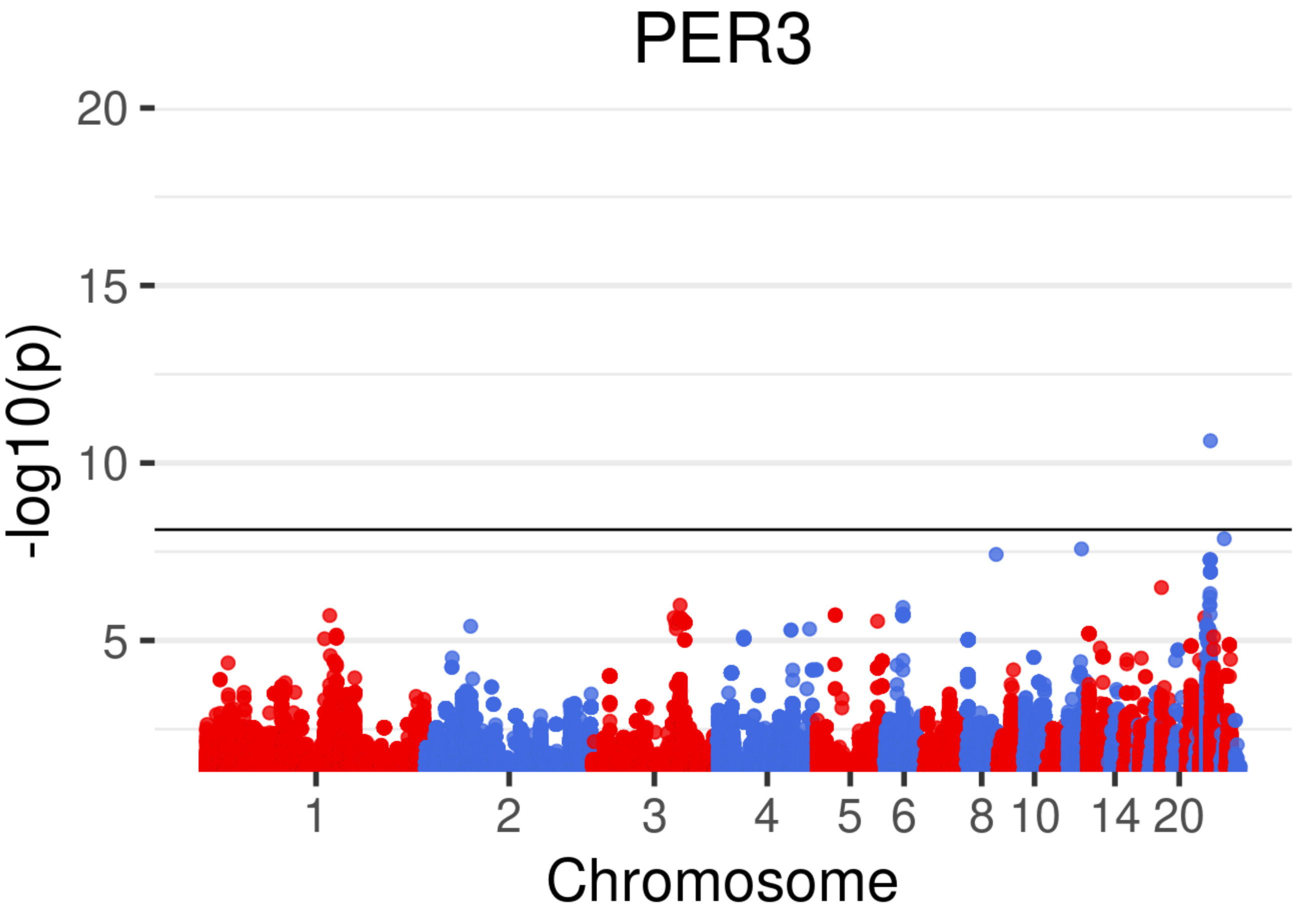
PER3
20
15
10
5
-log10(p)
1
2
3
4
5
6
8
10
14
20
Chromosome

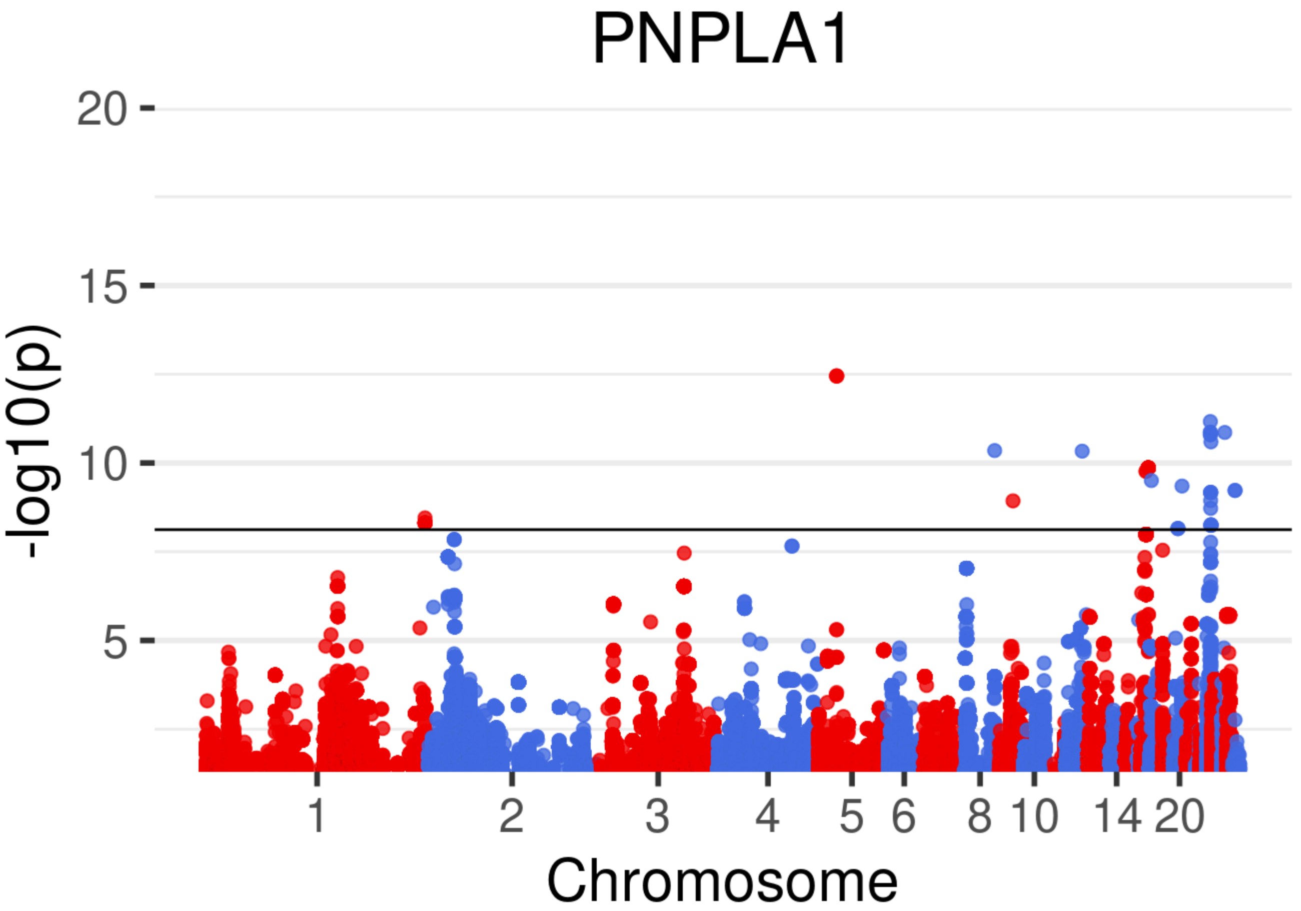

PNPLA1
20
15
10
5
-log10(p)
1
2
3
4
5
6
8
10
14
20
Chromosome

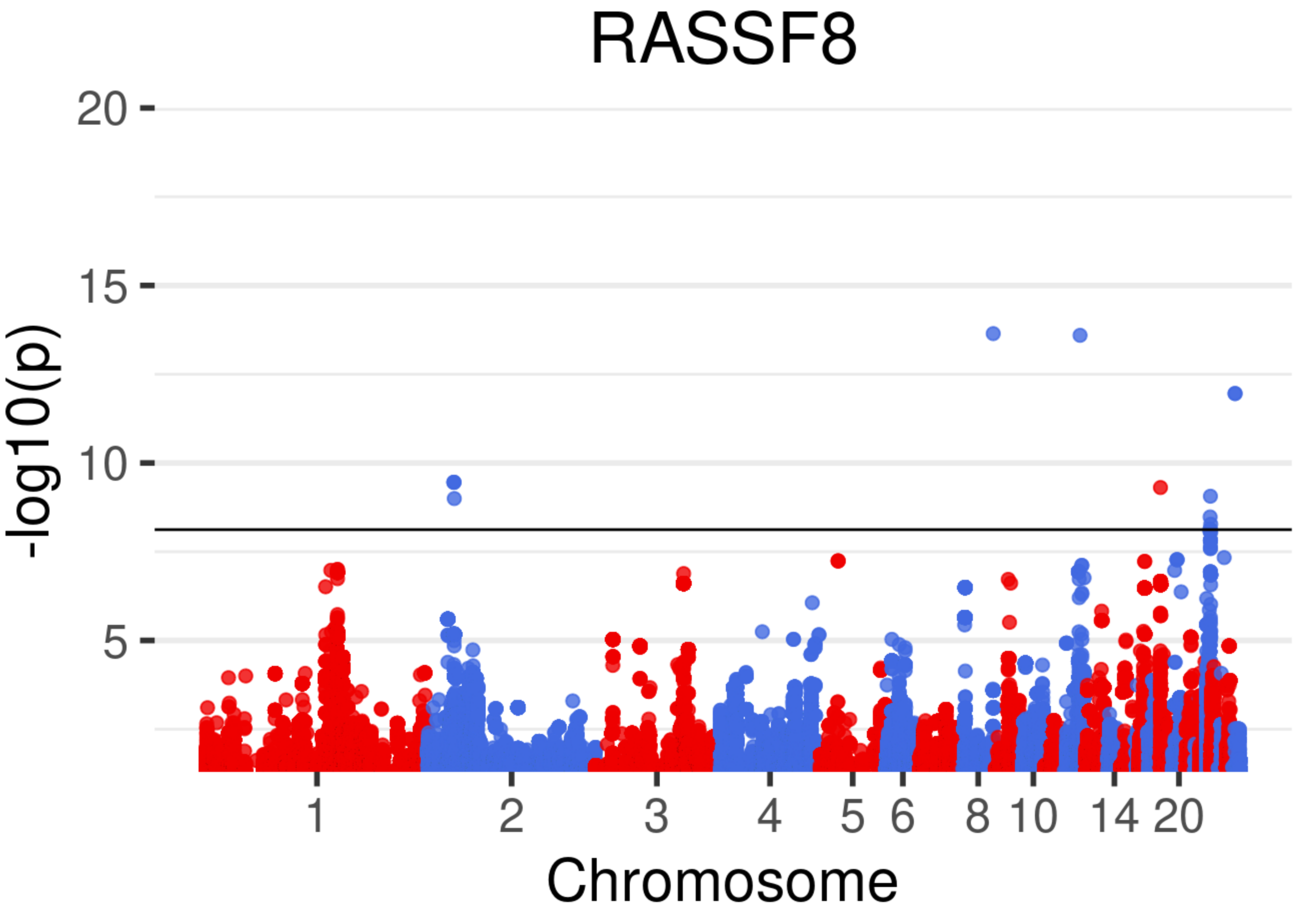
RASSF8
20
15
10
5
-log10(p)
1
2
3
4
5
6
8
10
14
20
Chromosome

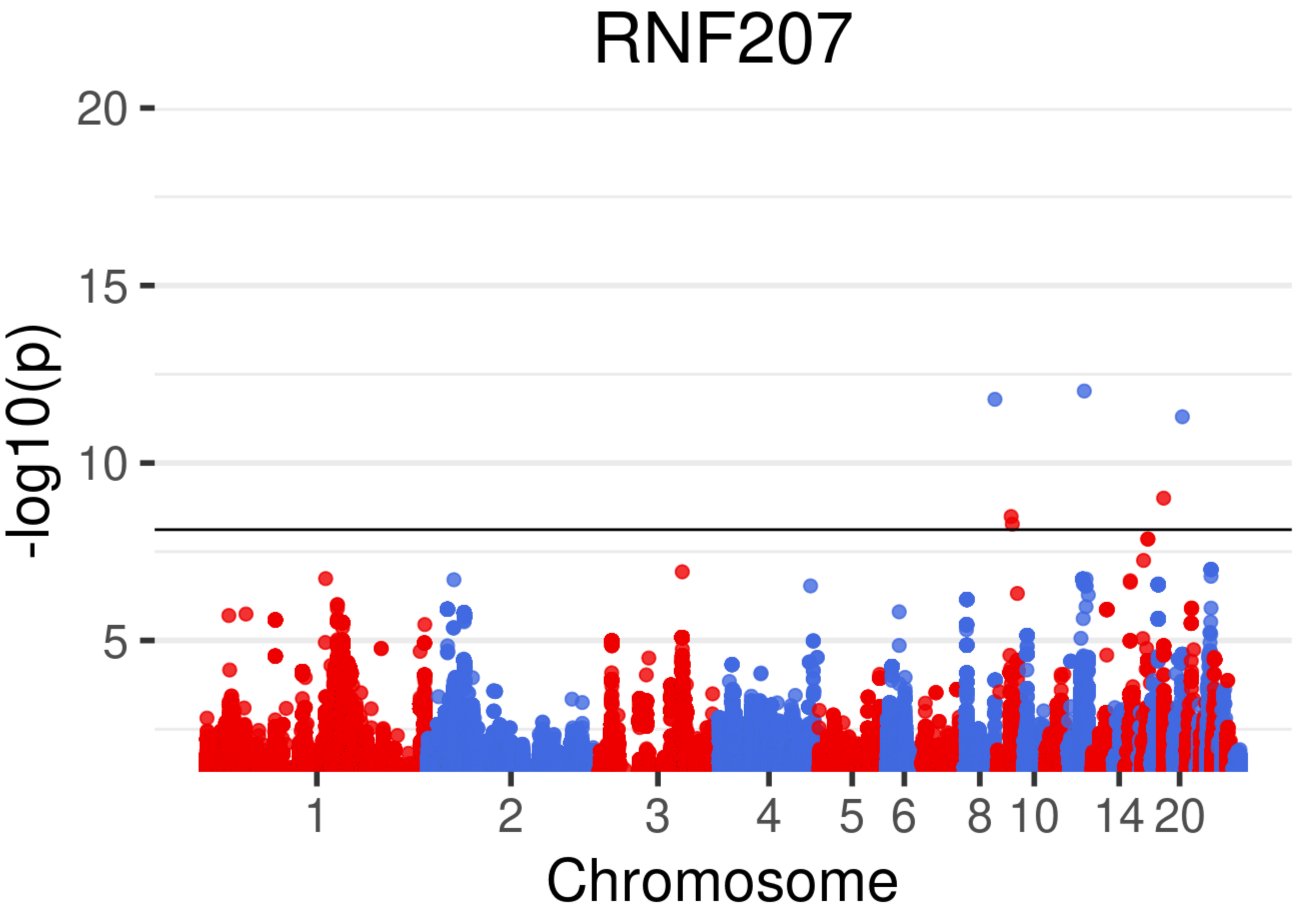

RNF207
20
15
10
5
-log10(p)
1
2
3
4
5
6
8
10
14
20
Chromosome

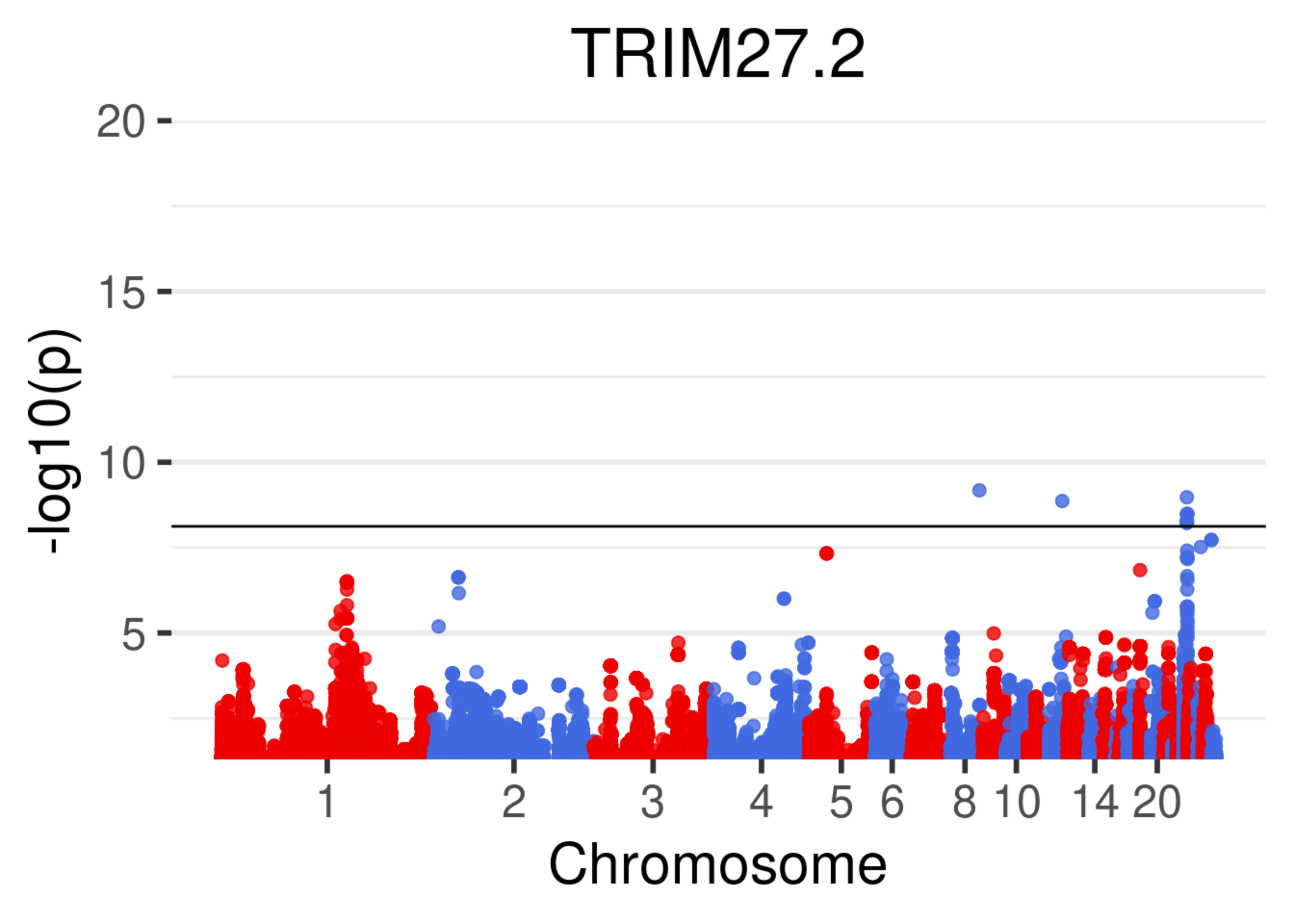

TRIM27.2
20
15
10
5
-log10(p)
1
2
3
4
5
6
8
10
14
20
Chromosome

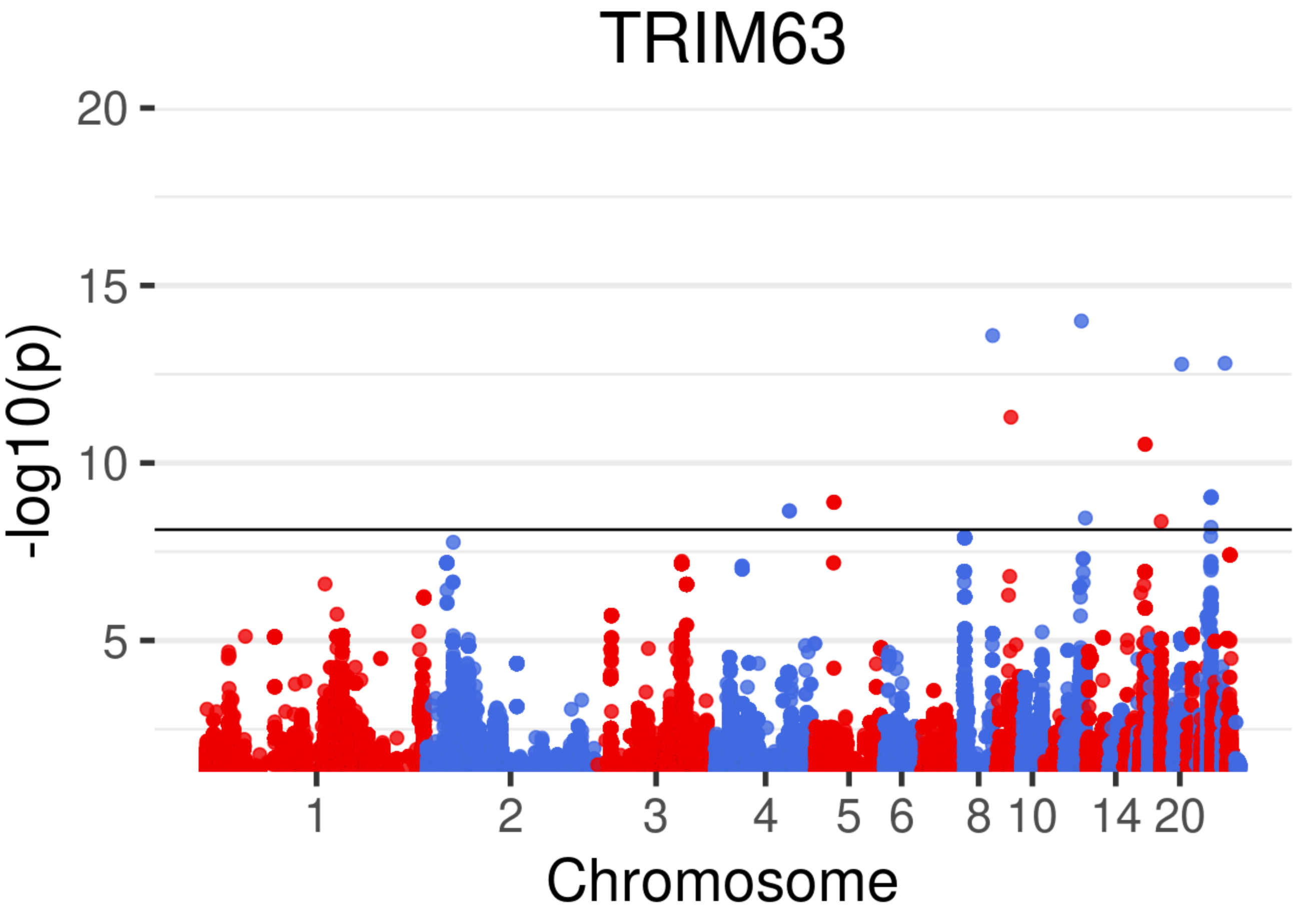

TRIM63
-log10(p)
20
15
10
5
1
2
3
4
5
6
8
10
14
20
Chromosome
